# TREM2‐Mediated Cholesterol Efflux in Macrophages Inhibits Anti‐Tumor Immunity via Limitation of CD4^+^ T and NK Cells

**DOI:** 10.1002/advs.202506995

**Published:** 2025-10-20

**Authors:** Yunhan Wang, Weina Yu, Xinxin Wang, Qitai Zhao, Qingyang Lei, Aitian Li, Shasha Liu, Tian Wang, Li Yang, Yi Zhang

**Affiliations:** ^1^ Biotherapy Center and Cancer Center The First Affiliated Hospital of Zhengzhou University Zhengzhou Henan 450052 China; ^2^ Zhongyuan Cell Therapy and Immunotherapy Laboratory Henan Academy of Innovations in Medical Science Zhengzhou Henan 450052 China; ^3^ School of Life Sciences Zhengzhou University Zhengzhou Henan 450052 China; ^4^ State Key Laboratory of Metabolic Dysregulation & Prevention and Treatment of Esophageal Cancer Tianjian Laboratory of Advanced Biomedical Sciences Academy of Medical Sciences Zhengzhou University Zhengzhou Henan 450052 China; ^5^ School of Public Health Zhengzhou University Zhengzhou Henan 450052 China

**Keywords:** anti‐tumor immunity, cholesterol, CX3CL1, TREM2, tumor microenvironment, tumor‐associated macrophages

## Abstract

Tumor‐associated macrophages (TAMs) predominantly exert functions that facilitate tumor progression. Triggering receptor expressed on myeloid cell 2 (TREM2) is expressed in TAMs, playing a crucial role in mediating the immunosuppressive function of TAMs. The mechanisms by which TREM2^+^ TAMs promote tumor growth and inhibit anti‐tumor immunity remain unclear. Through single‐cell sequencing of tumor tissues derived from wild‐type and *Trem2* knockout mice bearing subcutaneous lung cancer, it is found that TREM2 deletion hindered tumor growth, with a notable increase in and improved functionality of CD4^+^ T and natural killer (NK) cells in the tumor microenvironment. TREM2 deficiency led to ATP‐binding cassette transporter A1 (ABCA1) downregulation, causing cholesterol accumulation in TAMs and promoting a pro‐inflammatory phenotype. This results in increased chemokine (C‐X3‐C motif) ligand 1 (CX3CL1) secretion of macrophages, recruiting more CD4^+^ T and NK cells to the tumor site, enhancing the anti‐tumor response. After screening food and drug administration (FDA)‐approved drugs, bortezomib and ataluren are found to effectively inhibit TREM2 expression in TAMs, indicating a potential therapeutic strategy against TREM2. This study elucidates the mechanism by which TREM2 shapes the immunosuppressive microenvironment and promotes tumorigenesis, highlighting TREM2 as a target for cancer immunotherapy.

## Graphical abstract

Impaired anti‐tumor immunity is mediated by TREM2 + macrophages in non‐small cell lung cancer. TREM2 + macrophages prevent anti‐tumor immunity depending on the limitation of CD4 + T and NK cells. TREM2 promotes cholesterol efflux via ABCA1 to limit the production of CX3CL1 in macrophages. Bortezomib and ataluren enhance anti‐tumor efficacy by inhibiting the expression of TREM2.

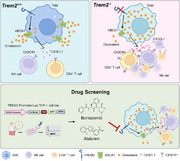

## 1 Introduction

The role of the immune system in tumorigenesis and progression has garnered significant attention, with tumor‐associated macrophages (TAMs), one of the main tumor‐infiltrating immune cell types, emerging as key regulators of the tumor microenvironment (TME) and playing a role in both anti‐tumor and pro‐tumor immune responses.^[^ ^1^, ^2^ ^]^ Macrophages can be roughly divided into pro‐inflammatory and alternatively activated types, which interconvert under different conditions and enable rapid adaptation to tumor development owing to their plasticity.^[^ ^3^, ^4^ ^]^ In this aggregate, TAMs predominantly exert functions that facilitate tumor progression.^[^ ^4^ ^]^ Therefore, identifying specific molecular markers and functional molecules of TAMs is crucial for understanding their role in the TME and for developing targeted immunotherapies.

The triggering receptor expressed on myeloid cells 2 (TREM2), a member of the immunoglobulin superfamily, is expressed in multiple types of macrophages, including Kupffer cells, microglia, alveolar macrophages, peritoneal macrophages, and TAMs.^[^ ^5^ ^]^ In the TME, TREM2 is considered a negative regulatory factor in anti‐tumor immunity, capable of inhibiting the proliferation of T cells, and is closely related to the poor prognosis of patients with cancer.^[^ ^6^ ^]^ Blockade of TREM2 improves T‐cell responses and enhances the therapeutic effect of anti‐programmed death protein 1 (PD‐1) therapy.^[^ ^7^ ^]^ In colorectal cancer and triple‐negative breast cancer, the expression of TREM2 was negatively correlated with the prolonged survival of patients with cancer.^[^ ^8^ ^]^

Previous studies have proposed that the function of TAMs is related to cholesterol metabolism. Atorvastatin inhibits HMG‐CoA reductase activity, reduces cholesterol synthesis, and blocks the inflammatory response in murine macrophages.^[^ ^9^ ^]^ Cholesterol efflux is an important step in macrophage‐mediated metabolism. In a mouse model of ovarian cancer, increased infiltration of TAM‐expressing genes related to cholesterol efflux was observed, accompanied by a decrease in the number of cholesterol‐rich microdomains on the cell membranes of macrophages.^[^ ^10^ ^]^ However, whether TREM2 mediates cholesterol metabolism to reprogram macrophages and affects anti‐tumor immunity remains unclear.

In this study, TREM2 is highly expressed in intratumoral anti‐inflammatory macrophages by single‐cell RNA sequencing (scRNA‐seq). TREM2 deficiency enhances anti‐tumor immunity via cholesterol accumulation‐induced chemokine (C‐X3‐C motif) ligand 1 (CX3CL1) production, which mediates CD4^+^ T and natural killer (NK) cell infiltration and remodeling of the TME. Our study revealed that targeting TREM2 using bortezomib and ataluren is a potential therapeutic strategy for cancer.

## 2 Results

### 2.1 TREM2 Exhibits Specific High Expression in Anti‐Inflammatory Macrophages

**Figure 1:**
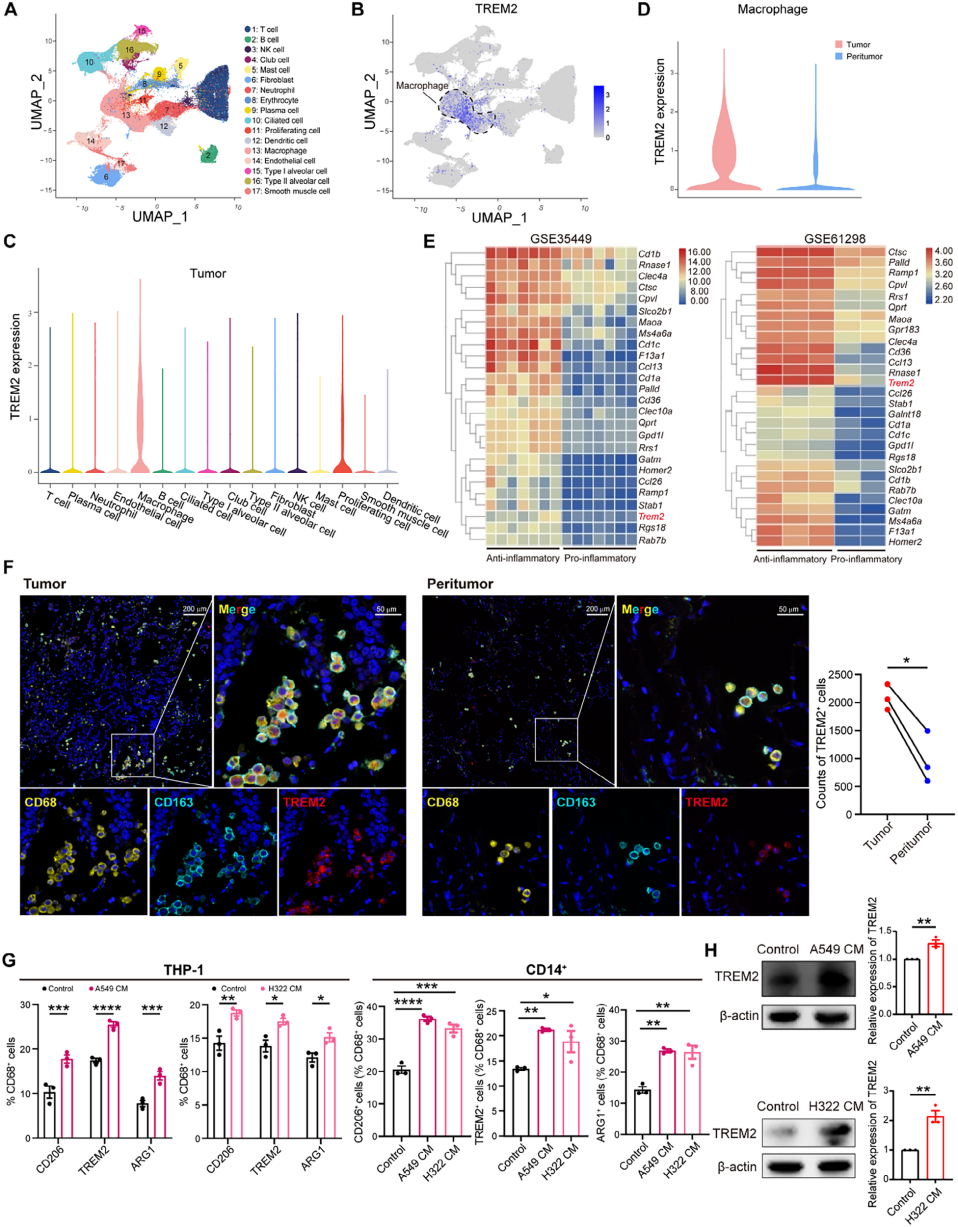
TREM2 is specifically highly expressed in anti‐inflammatory macrophage. A) UMAP visualization of 17 unique cell clusters from 10 paired tumor and peritumor tissues of NSCLC. Each dot represents a single cell. Colors denote the corresponding cell clusters. B) UMAP plot showing the expression of TREM2 in the main cell lineages by color. C) Violin plot showing the expression of TREM2 in various cell types from NSCLC tumor tissues. D) Violin plot showing the expression of TREM2 in macrophages in paired tumor and peritumor tissues of NSCLC. E) Heatmaps showing the comparison between 28 overlapping upregulated genes in anti‐inflammatory and pro‐inflammatory macrophages from the GSE35449 (left) and GSE61298 (right) datasets. Red, high expression; blue, low expression. The selected genes are highlighted. F) Immunofluorescence staining of CD68, CD163, TREM2, and DAPI (left) and corresponding analysis (right) in sections of tumor and peritumor tissues of NSCLC (n = 3 /group). Scale bars, 200 µm (overview), 50 µm (Enlarged view). G) Flow cytometry analysis of the percentages of CD206, TREM2, and ARG1 expressions on THP‐1 and CD14^+^ monocyte‐derived macrophages incubated with or without (control) A549 CM or H322 CM (n = 3 /group). H) Western blot analysis of TREM2 expression (left) and corresponding analysis (right) in THP‐1‐derived macrophages incubated with or without (control) A549 CM (up) or H322 CM (down) (n = 3 /group). Data represent mean ± SEM. Paired *t*‐test (F), two‐way analysis of variance (ANOVA) (G‐THP‐1), one‐way ANOVA (G‐CD14^+^), and unpaired *t*‐test (H) were performed. ^*^ *p* < 0.05; ^**^ *p* < 0.01; ^***^ *p* < 0.001; ^****^ *p* < 0.0001. UMAP, uniform manifold approximation and projection.

We first collected 10 surgically resected specimens from patients with non‐small cell lung cancer (NSCLC), including tumor and peritumoral tissues, and conducted in‐depth scRNA‐seq analysis. After stringent cell filtration, 161 123 cells were visualized using uniform manifold approximation and projection (UMAP), and 17 cell clusters were identified based on the expression of specific marker genes (**Figure** **1**A; Figure S1A, Supporting Information). During a detailed analysis of the gene expression profiles of these cell clusters, we focused on the expression of TREM2 and discovered that TREM2 was specifically and highly expressed in macrophages compared to other cell clusters (Figure 1B,C). We also performed an analysis using the Tumor Immune Single‐Cell Hub database, further verifying that TREM2 is specifically expressed in tumor‐infiltrating macrophages (Figure S1B, Supporting Information). In addition, the expression of TREM2 in tumor tissue‐derived macrophages was significantly higher than that in peritumoral macrophages (Figure 1D; Figure S2A, Supporting Information), emphasizing the potentially important role of intratumoral TREM2^+^ macrophages in NSCLC progression.

To further clarify whether TREM2 expression was related to pro‐ or anti‐inflammatory macrophages, we obtained the RNA‐seq datasets GSE35449 and GSE61298 from the GEO database and systematically analyzed the differentially expressed genes (DEGs) between induced anti‐inflammatory and pro‐inflammatory macrophages (Figure S2B, Supporting Information). We identified 28 genes that were upregulated in both datasets (Figure S2C, Supporting Information). Among the upregulated genes, TREM2 was predominantly expressed in anti‐inflammatory macrophages (Figure 1E). The immunofluorescence results showed that TREM2 colocalized with CD68^+^CD163^+^ anti‐inflammatory macrophages and that intratumoral TREM2^+^ macrophages were more highly infiltrated than those in peritumoral tissues (Figure 1F). Analysis of the TSIDB database revealed a positive correlation between TREM2 and multiple immunosuppressive molecules (Figure S2D, Supporting Information). To further evaluate the relationship between TREM2 and macrophage function, we used A549 cell‐conditioned medium (A549 CM) and H322 CM to induce THP‐1 cells and CD14^+^ monocytes to differentiate into M2‐like anti‐inflammatory macrophages in vitro. Notably, the expression levels of CD206, TREM2, and arginase 1 (ARG1) in A549 CM and H322 CM‐induced macrophages were significantly increased (Figure 1G,H). These results reveal a close correlation between TREM2 and anti‐inflammatory macrophages.

TREM2 showed a positive correlation with stromal, immune, and Estimate scores, which reflect the extent of stromal cell infiltration, extent of immune cell infiltration, and the sum of the stromal score and the immune score, which represents the overall non‐tumor cell components in the tumor microenvironment, respectively, thus indicating poor prognosis^[^ ^11^ ^]^ (Figure S3A, Supporting Information). According to the xCell algorithm, TREM2 was negatively correlated with tumor purity, which reflects the proportion of tumor cells in a tumor sample, and positively correlated with the level of anti‐inflammatory macrophage infiltration^[^ ^12^ ^]^ (Figure S3B, Supporting Information). Collectively, these findings suggest that TREM2 is associated with poor prognosis in patients with lung adenocarcinoma (LUAD). Through further analysis of the TCGA database, TREM2 levels in patients with metastatic LUAD were significantly higher than in those without metastasis (Figure S3C, Supporting Information). In addition, patients with high TREM2 levels had poor overall survival (OS) and relapse‐free survival (RFS) compared with those with low TREM2 levels (Figure S3D, Supporting Information). These findings suggest that TREM2 is specifically expressed in anti‐inflammatory macrophages and may serve as a potential biomarker for NSCLC prognosis.

### 2.2 TREM2 Deficiency Inhibits Tumor Growth by Remodeling the Immune Microenvironment

**Figure 2:**
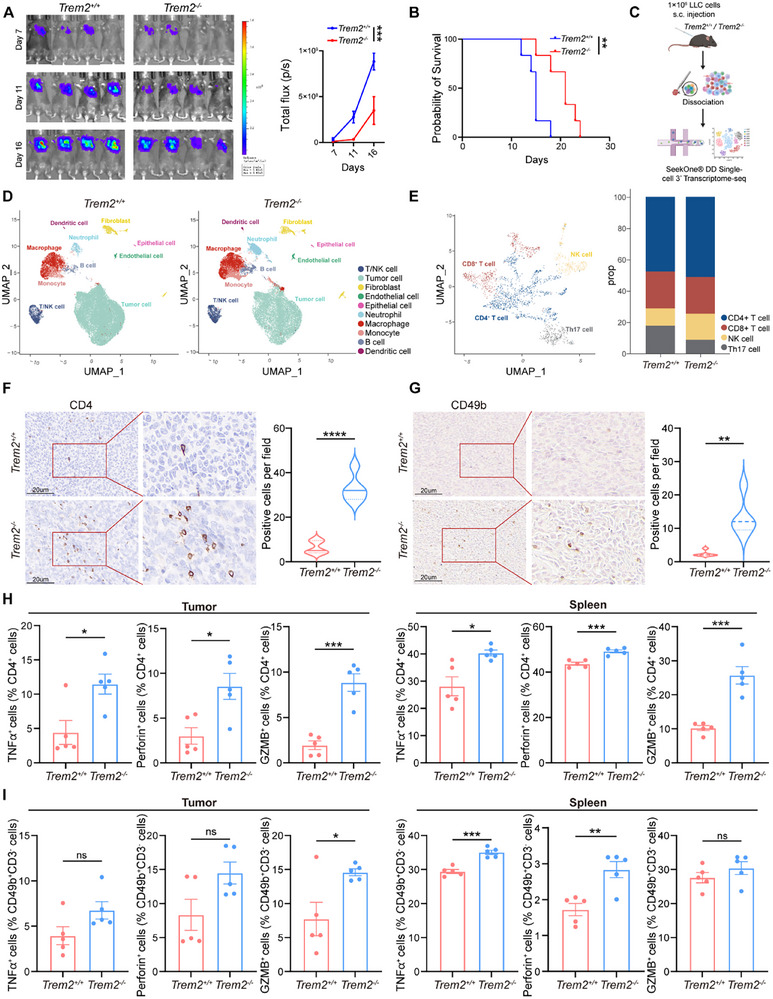
The deficiency of TREM2 remodels the tumor microenvironment and thereby enhances anti‐tumor function. A) Tumor bioluminescence images (left), and corresponding analysis (right) of *Trem2^+/+^* and *Trem2^−/−^* orthotopic lung cancer mice (n = 4 /group). B) Kaplan–Meier survival analysis of *Trem2^+/+^* and *Trem2^−/−^* orthotopic lung cancer mice (n = 6 /group). C) ScRNA‐seq profiling workflow. D) UMAP visualization of 10 unique cell clusters from *Trem2* ^+/+^ and *Trem2* ^−/−^ tumor tissues (n = 3 /group). Each dot represents a single cell. Colors denote the corresponding cell clusters. E) UMAP visualization of the T/NK cluster (left) and corresponding cell frequency changes (right) in *Trem2* ^+/+^ and *Trem2* ^−/−^ mice. F) Immunohistochemical staining of CD4 (left) and corresponding analysis (right) in tumor tissues from *Trem2* ^+/+^ and *Trem2* ^−/‐^ mice (n = 5 /group). Positive staining was indicated by brown coloration. Scale bar, 20 µm. G) Immunohistochemical staining of CD49b (left) and corresponding analysis (right) in tumor tissues from *Trem2* ^+/+^ and *Trem2* ^−/−^ mice (n = 5 /group). Positive staining was indicated by brown coloration. Scale bar, 20 µm. H,I) Flow cytometry analysis of the percentages of functional molecules on CD4^+^ T (H) and NK cells (I) in both tumor tissues and spleens from *Trem2* ^+/+^ and *Trem2* ^−/−^ mice (n = 5 /group). Data represent mean ± SEM. Two‐way analysis of variance (ANOVA) (A), Log‐Rank test (B), and unpaired *t*‐test (F, G, H, I) was applied. ns: no significance; ^*^ *p* < 0.05; ^**^ *p* < 0.01; ^***^ *p* < 0.001; ^****^ *p* < 0.0001.

To further investigate the relationship between TREM2 and tumor growth, we constructed an orthotopic lung cancer model and a subcutaneous tumor‐bearing model of lung cancer in wild‐type (*Trem2* ^+/+^) and *Trem2* knockout (*Trem2* ^−/−^) mice by directly injecting Lewis Lung Carcinoma (LLC) cells. We monitored tumor development and found that *Trem2* deficiency inhibited tumor progression and prolonged the survival of the mice (**Figures** **2**A,B; S4A, Supporting Information). To determine the functional role of TREM2 in intratumoral macrophages, the subcutaneous tumor tissues were harvested on day 10 after tumor inoculation and sequenced at the single‐cell level for an in‐depth analysis of the TME (Figure 2C). After rigorous cell filtration, 52845 cells were visualized using UMAP, and 10 cell clusters were identified based on the expression of specific marker genes (Figure 2D; Figure S4B, Supporting Information). By observing these cell clusters, we discovered an increase in the macrophage population in the *Trem2* ^−/‐^ group (Figure 2D). Further analysis of their functions revealed that compared to *Trem2* ^+/+^ mice‐derived macrophages, macrophages from *Trem2* ^−/‐^ mice exhibited a more pro‐inflammatory phenotype, whereas their anti‐inflammatory characteristics were markedly weakened (Figure S4C, Supporting Information).

An increase in T and NK cell infiltration was observed in the tumor tissues of the *Trem2^−/−^* mice (Figure 2D). Subsequently, the T and NK cell clusters were further divided into CD4^+^ T, CD8^+^ T, NK, and Th17 cells (Figure 2E; Figure S4D, Supporting Information). The proportion and activation of both CD4^+^ T and NK cells were upregulated in the *Trem2* ^−/‐^ group (Figure 2E; Figure S4E, Supporting Information). However, there was no significant change in the proportion of CD8^+^ T cells (Figure 2E). Next, we validated that both CD4^+^ T and NK cells were increased in the tumor tissues of *Trem2* ^−/−^ mice using immunohistochemistry (Figure 2F,G). We found that the expression of inflammatory factors, including tumor necrosis factor‐α (TNFα), perforin, and granzyme B (GZMB), by CD4^+^ T and NK cells was significantly increased in both tumor tissues and spleens of the *Trem2* ^−/−^ group (Figure 2H,I). These data suggest that the deficiency of TREM2 not only leads to an increase in CD4^+^ T and NK cells, but also enhances cytotoxicity, remodeling the TME to inhibit tumor growth.

### 2.3 Anti‐Tumor Immunity Mediated by TREM2 Deficiency in TAMs Mainly Depends on CD4^+^ T and NK Cells

**Figure 3:**
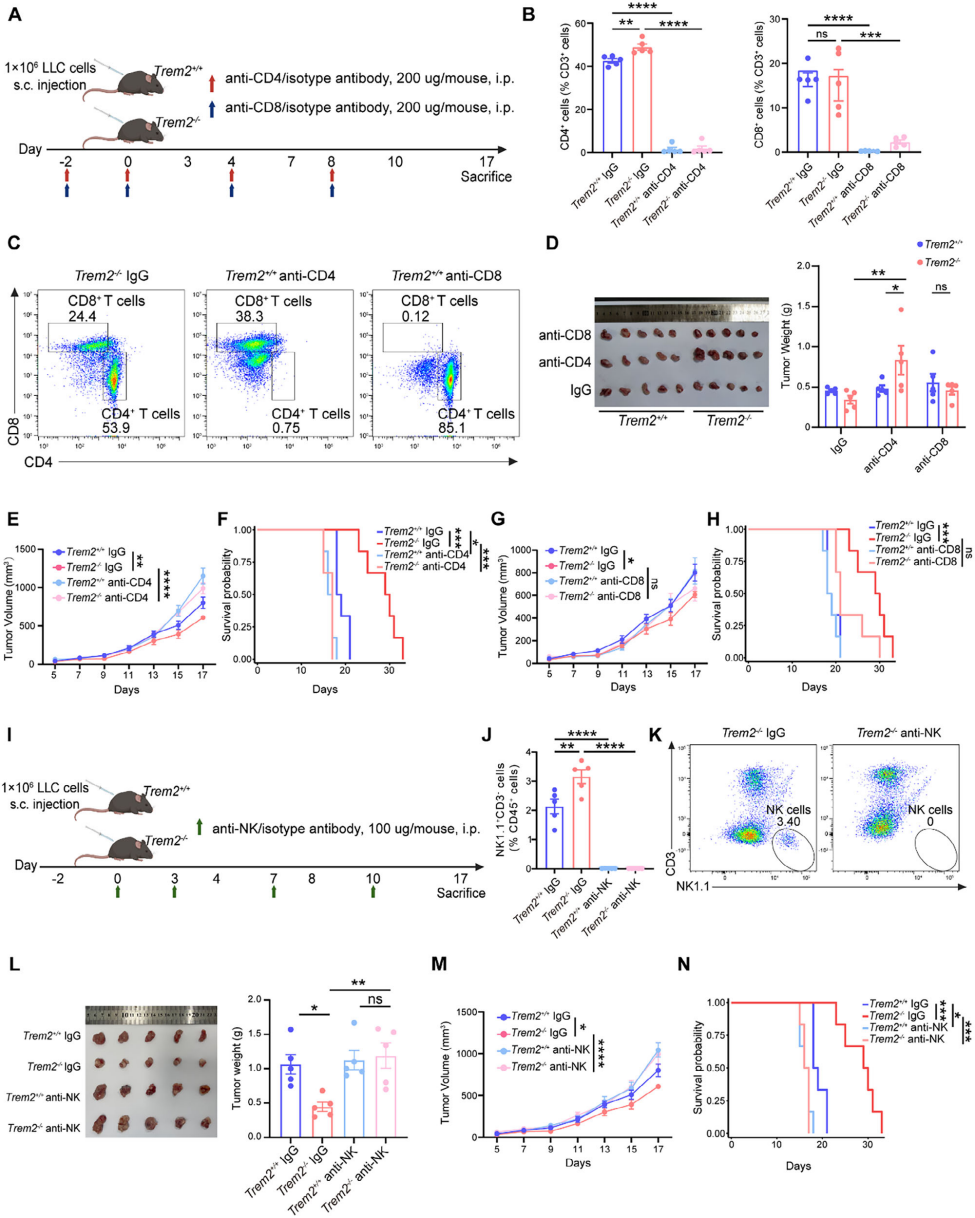
The anti‐tumor effect of TREM2 deficiency mainly depends on CD4^+^ T and NK cells. A) Schematic of experimental approach. 1 × 10^6^ LLC cells were subcutaneously (s.c.) implanted into *Trem2* ^+/+^ and *Trem2* ^−/−^ mice. At day ‐2, 0, 4, and 8 after tumor inoculation, mice were administered anti‐CD4, anti‐CD8 or IgG isotype antibodies (200 µg per mouse) intraperitoneally (i.p.). B) Flow cytometry analysis of the percentages of CD4^+^ and CD8^+^ T cells in the spleens of *Trem2* ^+/+^ and *Trem2* ^−/−^ mice treated with IgG isotype, anti‐CD4, or anti‐CD8 antibodies (n = 5 /group). C) Representative images showing flow cytometric analysis of CD4^+^ and CD8^+^ T cells in the spleens of mice after treatment with IgG isotype, anti‐CD4 or anti‐CD8 antibodies (n = 5 /group). D) Tumor images (left) and tumor weights (right) of *Trem2* ^+/+^ and *Trem2* ^−/−^ mice treated with IgG isotype, anti‐CD4 or anti‐CD8 antibodies were observed and measured 17 days after tumor inoculation (n = 5 /group). E) Tumor volumes of *Trem2* ^+/+^ and *Trem2* ^−/−^ mice treated with IgG isotype or anti‐CD4 antibody were measured until euthanization (n = 5 /group). F) Kaplan–Meier survival analysis of *Trem2* ^+/+^ and *Trem2* ^−/−^ mice treated with IgG isotype or anti‐CD4 antibody (n = 6 mice/group). G) Tumor volumes of *Trem2* ^+/+^ and *Trem2* ^−/−^ mice treated with IgG isotype or anti‐CD8 antibody were measured until euthanization (n = 5 /group). H) Kaplan–Meier survival analysis of *Trem2* ^+/+^ and *Trem2* ^−/−^ mice treated with IgG isotype or anti‐CD8 antibody (n = 6 /group). I) Schematic of the experimental approach. 1×10^6^ LLC cells were subcutaneously (s.c.) implanted into *Trem2* ^+/+^ and *Trem2* ^−/−^ mice. At day 0, 3, 7, and 10 after tumor inoculation, mice were administered anti‐NK or IgG isotype antibody (100 µg per mouse) intraperitoneally (i.p.). J) Flow cytometry analysis of the percentages of NK cells in the spleens of *Trem2* ^+/+^ and *Trem2* ^−/−^ mice treated with IgG isotype or anti‐NK antibody (n = 5 /group). K) Representative images showing flow cytometry analysis of NK cells in the spleens of mice after treatment with IgG isotype or anti‐NK antibody. L) Tumor images (left) and tumor weights (right) of *Trem2* ^+/+^ and *Trem2* ^−/−^ mice treated with IgG isotype or anti‐NK antibody were observed and measured 17 days after tumor inoculation (n = 5 /group). M) Tumor volumes of *Trem2* ^+/+^ and *Trem2* ^−/−^ mice treated with IgG isotype or anti‐NK antibody were measured until euthanization (n = 5 /group). N) Kaplan–Meier survival analysis of *Trem2* ^+/+^ and *Trem2* ^−/−^ mice treated with IgG isotype or anti‐NK antibody (n = 6 mice/group). Data represent mean ± SEM. One‐way analysis of variance (ANOVA) (B, J, L), two‐way ANOVA (D, E, G, M), and Log‐Rank test (F, H, N) were performed. ns: no significance; ^*^ *p* < 0.05; ^**^ *p* < 0.01; ^***^ *p* < 0.001; ^****^ *p* < 0.0001.

To further validate whether the anti‐tumor effects mediated by TREM2 deficiency relied on CD4^+^ T, CD8^+^ T, or NK cells, we individually depleted these three types of immune cells in both *Trem2* ^+/+^ and *Trem2* ^−/−^ mice and subsequently observed changes in tumor growth. Equal numbers of LLC cells (1 × 10^6^ cells per mouse) were subcutaneously injected into both *Trem2* ^+/+^ and *Trem2* ^−/‐^ C57BL/6N mice. In the experimental groups, anti‐CD4^+^ or anti‐CD8^+^ T‐depleting antibodies (200 µg per mouse) were administered intraperitoneally on days ‐2, 0, 4, and 8 after tumor inoculation^[^ ^13^, ^14^, ^15^ ^]^ (**Figure** **3**A). In the NK cell depletion group, anti‐NK antibodies (100 µg per mouse) were injected intraperitoneally on days 0, 3, 7, and 10 after tumor inoculation^[^ ^14^, ^16^ ^]^ (Figure 3I). *Trem2* ^+/+^ and *Trem2* ^−/‐^ mice injected with isotype antibodies served as the control groups. To dynamically observe tumor growth in mice, we regularly measured tumor volumes. On day 17 after tumor bearing, the mice were euthanized, and the tumors and spleens were collected for further analysis (Figure 3A,I).

CD4^+^ T, CD8^+^ T, and NK cells were effectively depleted (Figure 3B,C,J,K; Figure S5A,B, Supporting Information). Moreover, in the control groups of mice injected with isotype antibodies, the proportions of CD4^+^ T and NK cells in *Trem2* ^−/−^ mice were significantly higher than those in *Trem2* ^+/+^ mice, whereas the change in CD8^+^ T cells was not significant (Figure 3B,J), which echoed the results of previous scRNA‐seq (Figure 2E). Both the weight and volume of tumors in *Trem2* ^−/−^ mice were lower than those in *Trem2* ^+/+^ group. Subsequently, in *Trem2* ^−/−^ mice, the tumor weight and volume significantly increased after CD4^+^ T cell depletion, even exceeding those in *Trem2* ^+/+^ mice (Figure 3D,E), indicating that the anti‐tumor effect mediated by TREM2 deficiency was abolished by CD4^+^ T cell depletion. Moreover, the survival duration of *Trem2* ^−/−^ mice with CD4^+^ T cell depletion was significantly shorter than that of *Trem2* ^−/−^ IgG mice (Figure 3F). Following the depletion of CD8^+^ T cells, we observed no notable increase in tumor volume or weight (Figure 3D,G). Moreover, the survival duration of these mice was not significantly affected either (Figure 3H). Similarly, NK cell depletion reversed the tumor‐reducing phenotype in *Trem2* ^−/−^ mice; however, this phenomenon was not observed in *Trem2* ^+/+^ mice (Figure 3L,M). Additionally, *Trem2* ^−/−^ mice with NK cell depletion exhibited a notably shorter survival period than *Trem2* ^−/‐^ IgG mice (Figure 3N). The reduction in tumor burden, which depends on CD4^+^ T and NK cells, was only observed in TREM2 deficiency mice. Therefore, the anti‐tumor effect mediated by TREM2 deficiency in TAMs predominantly depends on CD4^+^ T and NK cells.

### 2.4 TREM2 Inhibits the Production of CX3CL1 from Macrophages

**Figure 4:**
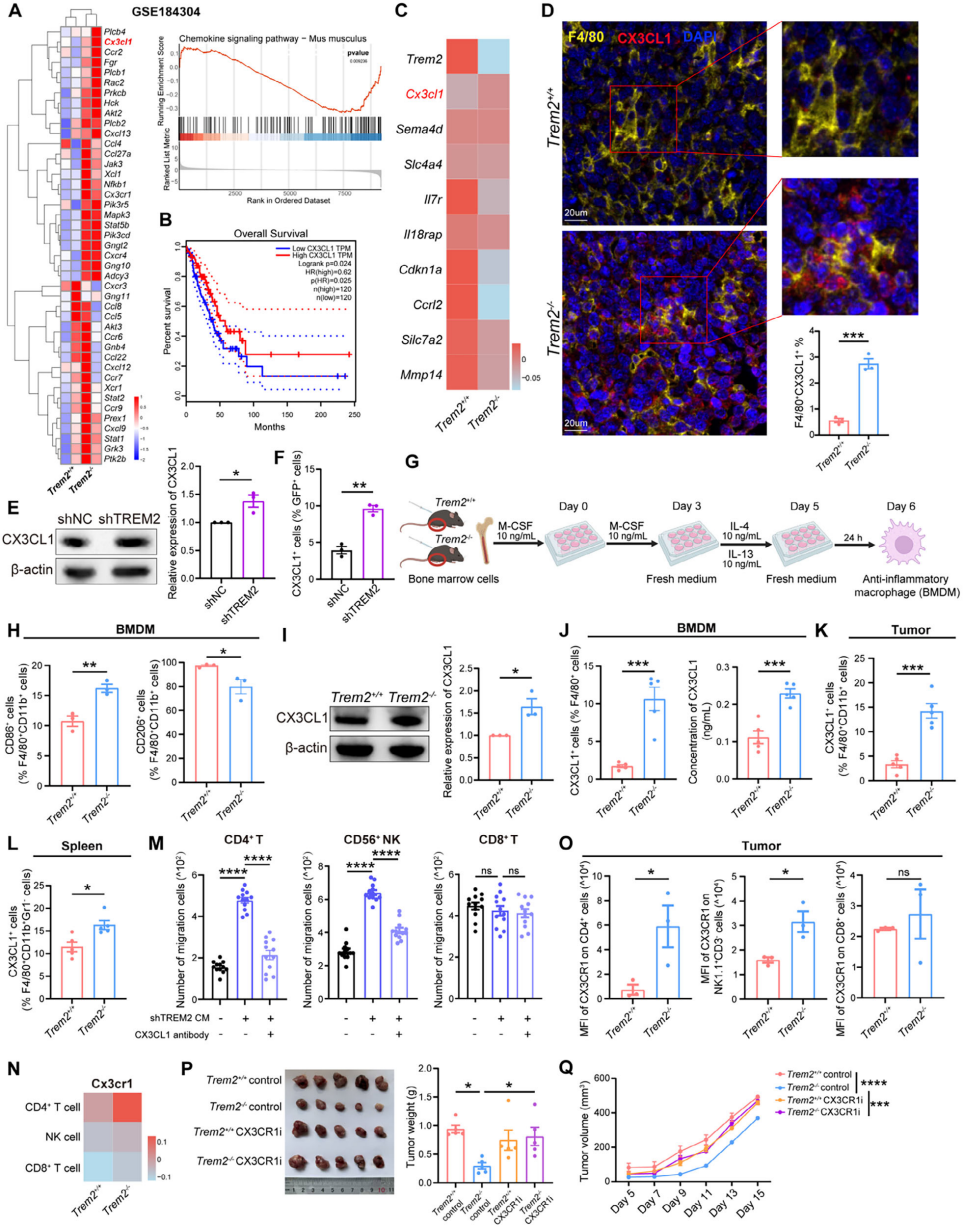
The absence of TREM2 promotes the production of CX3CL1 from macrophages. A) Heatmap of gene expression patterns (left) and gene set enrichment analysis (right) in pulmonary macrophages isolated from *Trem2* ^+/+^ and *Trem2* ^−/−^ mice from the GSE184304 scRNA‐seq dataset. Red, high expression; blue, low expression. Selected genes are highlighted. B) Kaplan–Meier analysis of the association between CX3CL1 expression and overall survival (OS) in patients with lung adenocarcinoma from the GEPIA database. C) Heatmap showing gene expression patterns of macrophages from tumor tissues of *Trem2* ^+/+^ and *Trem2* ^−/−^ mice using scRNA‐Seq data. Selected genes are highlighted. D) Immunofluorescence staining of F4/80, CX3CL1, and DAPI (left) and corresponding analysis (right) in tumor tissues from *Trem2* ^+/+^ and *Trem2* ^−/−^ mice (n = 3 /group). Scale bar, 20 µm. E) Western blot analysis of CX3CL1 expression (left) and corresponding analysis (right) in shNC or shTREM2 THP‐1‐derived macrophages (n = 3 /group). F) Flow cytometry analysis of the percentage of CX3CL1^+^ cells in shNC or shTREM2 THP‐1‐derived macrophages (n = 3 /group). G) Flowchart of the bone marrow‐derived macrophages (BMDMs) induction process. H) Flow cytometry analysis of the percentage of CD86^+^ cells and CD206^+^ cells in F4/80^+^ CD11b^+^ macrophages from BMDMs of *Trem2* ^+/+^ and *Trem2* ^−/−^ mice (n = 3 /group). I) Western blot analysis of CX3CL1 expression (left) and corresponding analysis (right) in BMDMs from *Trem2* ^+/+^ and *Trem2* ^−/−^ mice (n = 3 /group). J) Flow cytometry analysis of the percentage of CX3CL1^+^F4/80^+^ cells (left) and ELISA analysis of CX3CL1 levels in the supernatant (right) of BMDMs from *Trem2* ^+/+^ and *Trem2* ^−/−^ mice (n = 5 /group). K) Flow cytometry analysis of CX3CL1^+^ cells in F4/80^+^ CD11b^+^ macrophages from the tumor tissues of *Trem2* ^+/+^ and *Trem2* ^−/−^ mice (n = 5 /group). L) Flow cytometry analysis of the proportion CX3CL1^+^ cells in F4/80^+^CD11b^+^Gr1^−^ macrophages from the spleens of *Trem2* ^+/+^ and *Trem2* ^−/−^ mice (n = 5 /group). M) Transwell assay to examine the recruitment capacity of PBMC‐derived CD4^+^ T cells, CD56^+^ NK cells and CD8^+^ T cells treated with conditioned medium (CM) from shTREM2 THP‐1‐derived macrophages or CX3CL1 antibodies (n = 12 /group). N) Heatmap showing CX3CR1 expression in CD4^+^ T, NK, and CD8^+^ T cells from tumor tissues of *Trem2* ^+/+^ and *Trem2* ^−/−^ mice using scRNA‐Seq data. O) Flow cytometry analysis of the MFI of CX3CR1 on CD4^+^ cells, NK1.1^+^CD3^−^ cells and CD8^+^ cells from the tumor tissues of *Trem2* ^+/+^ and *Trem2* ^−/−^ mice (n = 3 /group). P) Tumor images (left) and tumor weights (right) of *Trem2* ^+/+^ and *Trem2* ^−/−^ mice treated with solvent or CX3CR1 inhibitor (CX3CR1i). Tumors were observed and measured 15 days after tumor inoculation (n = 5 /group). Q) Tumor volumes of *Trem2* ^+/+^ and *Trem2* ^−/−^ mice treated with solvent or CX3CR1i, measured until euthanization (n = 5 /group). Data represent mean ± SEM. Unpaired *t*‐test (D, E, F, H, I, J, K, L, O), one‐way analysis of variance (ANOVA) (M, P), and two‐way ANOVA (Q) were applied. ns: no significance; ^*^ *p* < 0.05; ^**^ *p* < 0.01; ^***^ *p* < 0.001; ^****^ *p* < 0.0001.

To further explore TREM2 deficiency‐induced increases in CD4^+^ T and NK cell infiltration in the TME, we performed scRNA‐seq analysis on pulmonary macrophages isolated from *Trem2* ^+/+^ and *Trem2* ^−/−^ mice using an online dataset (GSE184304). We explored the impact of TREM2 deficiency on macrophage function using Gene Set Enrichment Analysis (GSEA). The results showed that upregulated DEGs in macrophages from *Trem2* ^−/−^ mice were enriched in the chemokine signaling pathway, with *Cx3cl1* being the most notably differentially expressed chemokine (**Figure** **4**A). Therefore, we speculated that the absence of TREM2 might promote TAM programming into a pro‐inflammatory phenotype with high CX3CL1 production, recruiting more CD4^+^ T and NK cells. Furthermore, elevated expression levels of CX3CL1 are significantly associated with improved prognosis in patients with LUAD (Figure 4B). The expression level of CX3CL1 in peritumor tissues was significantly higher than that in the tumor tissues of patients with LUAD (Figure S6A, Supporting Information). Based on our scRNA‐seq data, the expression of *Cx3cl1* in tumor‐infiltrating macrophages from *Trem2* ^−/−^ mice was significantly higher than that in *Trem2* ^+/+^ mice (Figure 4C). We also observed a co‐localization phenomenon between CX3CL1 and tumor‐infiltrating macrophages, with more CX3CL1^+^ macrophages in the tumor tissues of *Trem2* ^−/−^ mice (Figure 4D).

To investigate the relationship between TREM2 expression and CX3CL1 production, we generated THP‐1 cells with TREM2 knockdown (shTREM2) using shRNA‐mediated lentiviral vectors. THP‐1 cells transfected with a non‐targeting plasmid (shNC) served as controls. The knockdown efficiency of TREM2 was assessed to confirm the successful establishment of the shTREM2 THP‐1 cell line (Figure S6B,C, Supporting Information). TREM2 knockdown promoted the programming of macrophages toward a pro‐inflammatory phenotype (Figure S6D, Supporting Information), which was consistent with the scRNA‐seq results (Figure S4C, Supporting Information). Moreover, the expression level of CX3CL1 in shTREM2 THP‐1‐derived macrophages was significantly increased compared to that in control cells (Figure 4E,F). To dissect the role of TREM2 in macrophages, we generated bone marrow‐derived macrophages (BMDMs) from *Trem2* ^+/+^ and *Trem2* ^−/−^ mice, bone marrow cells were collected and induced into anti‐inflammatory macrophages in vitro (Figure 4G; Figure S6E, Supporting Information). TREM2 deficiency promoted the programming of BMDMs toward a pro‐inflammatory phenotype, characterized by upregulated CD86 and downregulated CD206 expression (Figure 4H). CX3CL1 levels were also significantly elevated in BMDM, tumor‐infiltrating macrophages, and macrophages of spleens from *Trem2* ^−/−^ mice compared to those from *Trem2* ^+/+^ mice (Figure 4I–L; Figure S6F, Supporting Information).

CX3CL1 is a multifunctional inflammatory chemokine, and its biological effects are mediated through its unique receptor chemokine (C‐X3‐C motif) receptor 1 (CX3CR1), which is expressed on T, NK, and B cells.^[^ ^17^, ^18^ ^]^ To investigate the recruitment of CX3CL1 to immune cells, we conducted a transwell experiment using recombinant human CX3CL1 (rhCX3CL1) and peripheral blood mononuclear cells (PBMCs) from healthy individuals. The results indicated that rhCX3CL1 exerted a significant chemotactic effect on CD4⁺ T cells and CD56⁺ NK cells (Figure S6G, Supporting Information), indicating that CD4^+^ T and CD56^+^ NK cells are capable of recruitment in response to the CX3CL1‐CX3CR1 signaling axis. rhCX3CL1 recruited CD4^+^ T cells and CD56^+^ NK cells in a concentration‐dependent manner (Figure S6H, Supporting Information). Similarly, CX3CL1 enhanced the cytotoxicity of CD4^+^ T and NK cells with high levels of interferon γ (IFNγ), perforin, and GZMB (Figure S6I,J, Supporting Information). To further investigate whether TREM2 deficiency mediates the recruitment of CD4^+^ T and NK cells via CX3CL1, conditioned medium of shTREM2 THP‐1‐derived macrophages (shTREM2 CM) was used to promote the migration of CD4^+^ T and NK cells. However, this effect was reversed by the addition of CX3CL1‐neutralizing antibodies. However, the recruitment of CD8^+^ T cells was unaffected by CX3CL1‐neutralizing antibodies (Figure 4M). The scRNA‐seq results of *Trem2* ^+/+^ and *Trem2* ^−/−^ mice showed significantly higher levels of CX3CR1 in CD4^+^ T and NK cells from *Trem2* ^−/−^ mice (Figure 4N), which was further validated by flow cytometry and immunohistochemistry (Figure 4O; Figure S6K,L, Supporting Information). However, there was no significant difference in CX3CR1 expression on CD8^+^ T cells between *Trem2* ^+/+^ and *Trem2* ^−/−^ mice (Figure 4O). Furthermore, within *Trem2* ^−/−^ mice, the expression of CX3CR1 is markedly elevated in CD4^+^ T and NK cells compared to its level in CD8^+^ T cells (Figure S6M, Supporting Information). This finding demonstrates that CD8^+^ T cells may not be required via the CX3CL1‐CX3CR1 axis. To determine the functional role of CX3CL1 in TREM2‐mediated immunosuppression, we challenged *Trem2* ^+/+^ and *Trem2* ^−/−^ mice with LLC cells (1 × 10^6^ cells per mouse) to establish a subcutaneous lung cancer model. We divided the mice into CX3CR1 inhibitor (CX3CR1i) ‐treated and control groups. After tumor inoculation, CX3CR1i or solvent was injected intraperitoneally daily for 2 weeks, and tumor volumes were measured every 2 days. The results revealed that in *Trem2* ^−/−^ mice, CX3CR1 inhibition significantly promoted tumor growth (Figure 4P,Q), reversing the tumor‐suppressive effect induced by *Trem2* knockout. This in vivo evidence confirms the key role of the CX3CL1‐CX3CR1 axis in the anti‐tumor immunity induced by *Trem2* deletion. These results indicate that TREM2‐deficient macrophages facilitate the recruitment of CD4^+^ T and NK cells through increased production of CX3CL1, triggering stronger anti‐tumor immunity.

### 2.5 TREM2 Promotes Cholesterol Efflux, thereby Reprogramming Macrophages toward an Anti‐Inflammatory Phenotype

**Figure 5:**
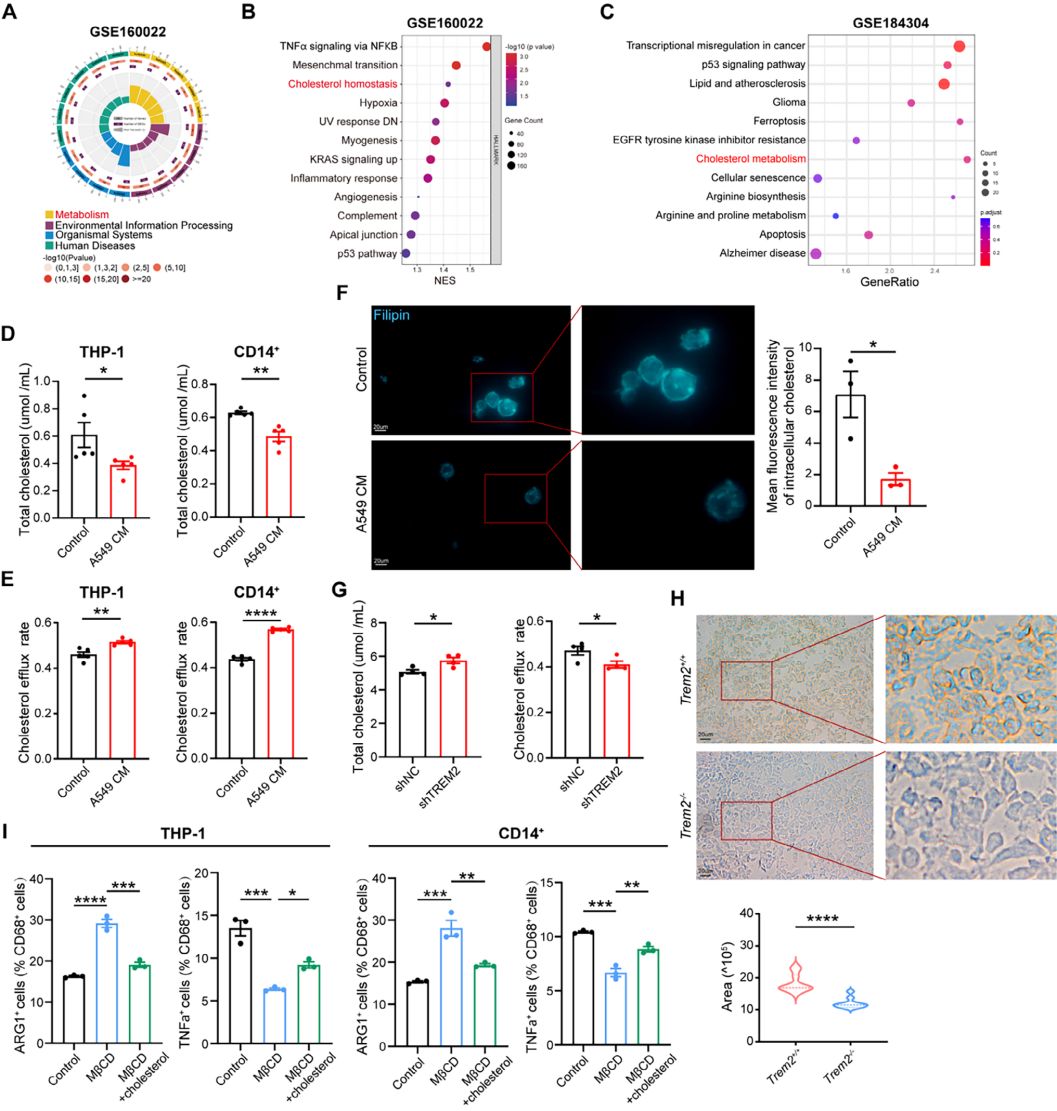
The absence of TREM2 increases cholesterol accumulation in macrophages. A) KEGG pathway enrichment analysis of upregulated DEGs in liver macrophages from *Trem2* ^−/−^ mice compared to *Trem2* ^+/+^ mice using the OmicShare website (GSE160022). B) GSEA pathway enrichment analysis of macrophage in *Trem2* ^+/+^ and *Trem2* ^−/−^ mice from GSE160022 RNA‐seq dataset. NES: Normalized Enrichment Score. C) GSEA pathway enrichment analysis of pulmonary macrophage in *Trem2* ^+/+^ and *Trem2* ^−/−^ mice from the GSE184304 scRNA‐seq dataset. D) Statistical chart showing intracellular total cholesterol in THP‐1 (left) and CD14^+^ monocyte (right)‐derived macrophages incubated with or without (control) A549 CM (n = 5 /group). E) Statistical chart showing cholesterol efflux rate in THP‐1 (left) and CD14^+^ monocyte (right)‐derived macrophages incubated with or without (control) A549 CM (n = 5 /group). F) Filipin staining of intracellular cholesterol levels (left) and corresponding analysis (right) in THP‐1‐derived macrophages incubated with or without (control) A549 CM (n = 3 /group). Scale bar, 20 µm. G) Statistical chart showing the intracellular total cholesterol (left) and cholesterol efflux rate (right) in shNC or shTREM2 THP‐1‐derived macrophages (n = 4 /group). H) Schematic diagram of cholesterol staining (up) and corresponding analysis (down) of cell membranes from cryosectioned tumor tissues of *Trem2* ^+/+^ and *Trem2* ^−/−^ mice (n = 10 /group). Scale bar, 20 µm. I) Flow cytometry analysis of the percentage of ARG1^+^ and TNFα^+^ cells in CD68^+^ cells from THP‐1 (left) and CD14^+^ monocyte (right)‐derived macrophages treated with or without cholesterol (10 ug mL−^1^) or MβCD (n = 3 /group). Data represent mean ± SEM. Unpaired *t*‐test (D, E, F, G, H) and one‐way analysis of variance (ANOVA) I) were applied were applied was applied. ^*^ *p* < 0.05; ^**^ *p* < 0.01; ^***^ *p* < 0.001; ^****^ *p* < 0.0001.

Next, we investigated the mechanism by which TREM2 limited CX3CL1 production by macrophages. We analyzed the DEGs in the RNA‐seq data (GSE160022) of macrophages from *Trem2* ^+/+^ and *Trem2* ^−/−^ mice (Figure S7A,B, Supporting Information). The KEGG pathway analysis revealed enrichment of metabolic pathways among the upregulated genes in *Trem2* ^−/−^ mice (**Figure** **5**A). We then performed GSEA on DEGs from RNA‐seq data of *Trem2* ^+/+^ and *Trem2* ^−/−^ mice, which revealed notable enrichment in cholesterol homeostasis‐related pathways (Figure 5B). Similarly, the GSEA of DEGs from scRNA‐seq data (GSE184304) of *Trem2* ^+/+^ and *Trem2* ^−/−^ mice showed that cholesterol metabolism was strongly enriched in macrophages (Figure 5C). Because intracellular cholesterol accumulation can activate inflammatory signaling pathways in macrophages, leading to a pro‐inflammatory anti‐tumoral phenotype,^[^ ^19^ ^]^ we hypothesized that TREM2 regulates the cholesterol efflux process in macrophages, influences intracellular cholesterol accumulation, and ultimately alters CX3CL1 production.

To validate our hypothesis, we measured the intracellular cholesterol levels and cholesterol efflux rates in the A549 CM‐treated macrophages and the control group. Compared to the control group, THP‐1 and CD14^+^ monocyte‐derived macrophages treated with A549CM exhibited a decrease in intracellular total cholesterol content (Figure 5D) and an increase in the cholesterol efflux rate (Figure 5E). Further validation using the Filipin staining assay demonstrated that the intracellular cholesterol level of A549 CM‐treated macrophages was lower than that of the control group (Figure 5F). We also measured the cholesterol levels in shTREM2 and shNC THP‐1‐derived macrophages. We observed that shTREM2 THP‐1‐derived macrophages showed a significant decrease in the cholesterol efflux rate, accompanied by an increase in the intracellular cholesterol content (Figure 5G). To investigate the effect of TRME2 deficiency on cholesterol levels in tumor tissues, we analyzed cholesterol levels in tumor tissues from *Trem2* ^+/+^ and *Trem2* ^−/−^ mice, showing that cholesterol levels in the cell membranes of *Trem2* ^−/−^ mice were significantly lower than those of *Trem2* ^+/+^ group, suggesting impaired cholesterol efflux and subsequent accumulation of cholesterol within the cells (Figure 5H). This also indirectly supports the role of TREM2 in macrophage cholesterol metabolism.

Methyl‐β‐cyclodextrin (MβCD) can deplete cholesterol from the cell membrane, facilitating cholesterol efflux in macrophages,^[^ ^20^ ^]^ whereas water‐soluble cholesterol can replenish intracellular cholesterol levels.^[^ ^21^ ^]^ To further investigate the impact of cholesterol metabolism on macrophage function, we introduced MβCD or cholesterol into the culture system of THP‐1 and CD14^+^ monocyte‐derived macrophages. Treatment with MβCD upregulated ARG1 expression in CD68^+^ macrophages, which was reversed by subsequent treatment with cholesterol (Figure 5I; Figure S7C,D, Supporting Information). Furthermore, the MβCD‐induced decrease in intracellular cholesterol content resulted in decreased TNFα expression in CD68^+^ macrophages, thus promoting polarization toward an anti‐inflammatory phenotype. However, subsequent treatment with cholesterol reversed these effects (Figure 5I; Figure S7C,D, Supporting Information). These results indicate that TREM2 promotes cholesterol efflux in macrophages, thereby reprogramming these cells toward an anti‐inflammatory phenotype.

### 2.6 TREM2 Promotes Macrophage Cholesterol Efflux via ABCA1 to Limit CX3CL1 Production

**Figure 6:**
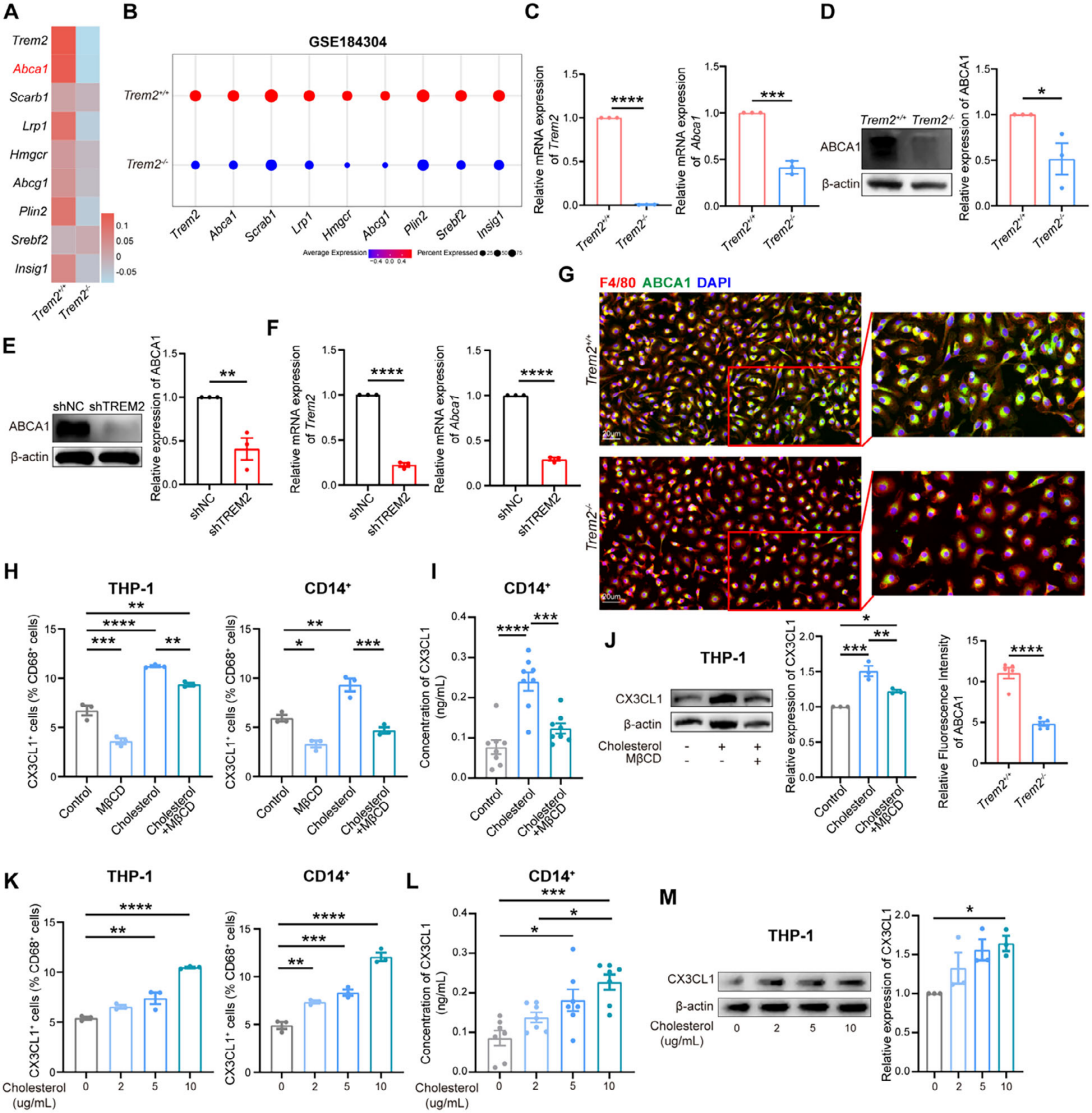
The downregulation of ABCA1 results in the accumulation of cholesterol. A) Heatmap showing the gene expression patterns of macrophages from the tumor tissues of *Trem2^+/+^* and *Trem2^−/−^* mice using scRNA‐seq data. Selected gene is highlighted. B) Expression of cholesterol metabolism genes of *Trem2^+/+^* and *Trem2^−/−^* macrophages using GSE184304 scRNA‐seq data. C) qPCR analysis of *Trem2* (left) and *Abca1* (right) expression in BMDMs from *Trem2^+/+^* and *Trem2^−/−^* mice (n = 3 /group). D) Western blot analysis of ABCA1 expression (left) and corresponding analysis (right) in BMDMs from *Trem2^+/+^* and *Trem2^−/−^* mice (n = 3 /group). E) Western blot analysis of ABCA1 expression (left) and corresponding analysis (right) in shNC or shTREM2 THP‐1‐derived macrophages (n = 3 /group). F) qPCR analysis of *Trem2* (left) and *Abca1* (right) expression in shNC or shTREM2 THP‐1‐derived macrophages (n = 3 /group). G) Immunofluorescence staining of F4/80, ABCA1, and DAPI (up) and corresponding analysis (down) in BMDMs from *Trem2^+/+^* and *Trem2^−/−^* mice (n = 5 /group). Scale bar, 20 µm. H) Flow cytometry analysis of CX3CL1 expression in THP‐1 and CD14^+^ monocyte‐derived macrophages after treatment with or without MβCD and cholesterol (10 ug/mL) (n = 3 /group). I) ELISA analysis of CX3CL1 levels in the supernatant of CD14^+^ monocyte‐derived macrophages after treatment with or without MβCD and cholesterol (10 ug mL^−1^) (n = 8 / group). J) Western blot analysis of CX3CL1 expression (left) and corresponding analysis (right) in THP‐1‐derived macrophages after treatment with or without (control) MβCD and cholesterol (10 ug mL^−1^) (n = 3 /group). K) Flow cytometry analysis of CX3CL1 in THP‐1 and CD14^+^ monocytes‐derived macrophages treated with increasing concentration of cholesterol (n = 3 /group). L) ELISA analysis of CX3CL1 levels in the supernatant of CD14^+^ monocyte‐derived macrophages after treatment with increasing concentrations of cholesterol (n = 7 /group). M) Western blot analysis of CX3CL1 expression (left) and corresponding analysis (right) in THP‐1‐derived macrophages after treatment with increasing concentrations of cholesterol (n = 3 /group). Data represent mean ± SEM. Unpaired t test (C, D, E, F, G) and one‐way analysis of variance (ANOVA) (H, I, J, K, L, M) were applied. ns: no significance; ^*^ *p* < 0.05; ^**^ *p* < 0.01; ^***^ *p* < 0.001; ^****^ *p* < 0.0001.

In order to delve into the mechanisms regulating cholesterol efflux in macrophages, we conducted an analysis of cholesterol‐related genes in macrophages using our scRNA‐seq data from tumor tissues of *Trem2* ^+/+^ and *Trem2* ^−/−^ mice, which revealed that the most prominent DEG was ATP‐binding cassette transporter A1 (ABCA1) (**Figure** **6**A), a cholesterol transporter involved in the efflux of intracellular cholesterol.^[^ ^22^ ^]^ We validated this phenomenon in pulmonary macrophages using the online database GSE184304 (Figure 6B). Hence, we hypothesized that the absence of *Trem2* triggers the downregulation of ABCA1 in macrophages, resulting in restricted cholesterol efflux, which in turn causes the accumulation of cholesterol in macrophages.

To test this hypothesis, we performed subsequent validation experiments and observed that there was a significant downregulation of ABCA1 expression in BMDMs derived from *Trem2* ^−/−^ mice, as well as in shTREM2 THP‐1‐derived macrophages (Figure 6C–G; Figure S7E, Supporting Information). This demonstrates that TREM2 deficiency gives rise to the downregulation of ABCA1 in macrophages, which leads to intracellular cholesterol accumulation. Subsequently, we intend to explore the impact of alterations in cholesterol levels within macrophages on their secretion of CX3CL1. Therefore, we treated THP‐1 and CD14^+^ monocyte‐derived macrophages with cholesterol or MβCD to assess changes in CX3CL1 expression. The results revealed that MβCD reduced CX3CL1 expression in macrophages, whereas cholesterol had the opposite effect. Sequential treatment with cholesterol followed by MβCD reversed the increased CX3CL1 expression level (Figure 6H–J). Similarly, MβCD reduced CX3CL1 expression in shTREM2 THP‐1‐derived macrophages (Figure S7F, Supporting Information). Moreover, CX3CL1 levels increased with increasing cholesterol concentrations in THP‐1 and CD14^+^ monocyte‐derived macrophages, indicating a gradient‐dependent link with cholesterol levels (Figure 6K–M). These findings demonstrated that TREM2 promotes cholesterol efflux via ABCA1 and inhibits CX3CL1 release by macrophages.

### 2.7 Bortezomib and Ataluren Enhance Anti‐Tumor Efficacy by Inhibiting TREM2 to Reshape the Tumor Microenvironment

**Figure 7:**
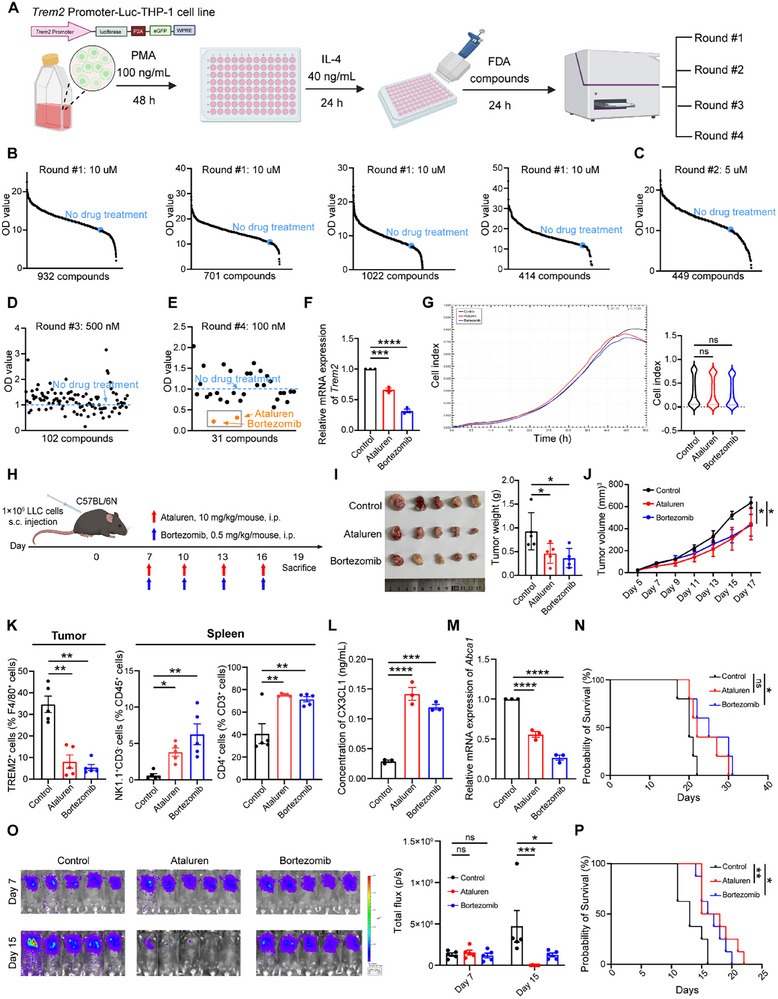
Ataluren and bortezomib reduce TREM2 expression and delay tumor growth in vivo. A) Schematic workflow for drug screening. B–E). Four rounds of screening were performed for test compounds at different doses. Compounds with OD values lower than those of the no‐drug treatment were selected for the next round of screening. F) qPCR analysis of *Trem2* expression in THP‐1‐derived macrophages after treatment with or without (control) ataluren or bortezomib (n = 3 /group). G) Real‐time intelligent monitoring of the viability of THP‐1 cells in response to treatment with ataluren and bortezomib (n = 4 /group). H) Schematic of experimental approach. 1 × 10^6^ LLC cells (s.c.) implanted into C57BL/6N mice. On days 7, 10, 13, and 16 after tumor inoculation, mice were intraperitoneally (i. p.) treated with or without (control) ataluren (1.0 mg per kg per mouse) or bortezomib (0.5 mg per kg per mouse) intraperitoneally (i.p.) (n = 5 /group). I) Tumor images (left) and tumor weights (right) of C57BL/6N mice were observed and measured until euthanization after treatment with or without (control) ataluren or bortezomib (n = 5 /group). J) Tumor volumes of C57BL/6N mice treated with or without (control) ataluren or bortezomib were measured until euthanization (n = 5 /group). K) Flow cytometry analysis of TREM2^+^ cells in the tumor tissues, NK1.1^+^CD3^−^ cells, and CD4^+^ cells in the spleens of C57BL/6N mice treated with or without (control) ataluren or bortezomib (n = 5 /group). L) CX3CL1 levels in the supernatant of BMDMs from C57BL/6N mice treated with or without (control) ataluren or bortezomib were analyzed (n = 3 /group). M) qPCR analysis of *Abca1* expression in BMDMs from C57BL/6N mice after treatment with or without (control) ataluren or bortezomib (n = 3/group). N) Kaplan‐Meier survival curve for mice treated with or without (control) ataluren or bortezomib in a subcutaneous tumor‐bearing mouse model (n = 5 /group). O) Tumor bioluminescence images (left) and corresponding analysis (right) of C57BL/6N mice after treatment with or without (control) ataluren or bortezomib (n = 5 /group). P) Kaplan–Meier survival analysis of C57BL/6N mice in the orthotopic lung cancer model treated with or without (control) ataluren or bortezomib (n = 8 /group). Data represent mean ± SEM. One‐way analysis of variance (ANOVA) (F, G, I, K, L, M), two‐way ANOVA (J, O), and Log‐Rank test (N, P) were applied. ns: no significance; ^*^ *p* < 0.05; ^**^ *p* < 0.01; ^***^ *p* < 0.001; ^****^ *p* < 0.0001.

Finally, we conducted FDA‐approved drug screening to identify drugs that reduce TREM2 expression to provide a potential strategy for tumor therapy.^[^ ^23^ ^]^ THP‐1 cells were lentivirally transduced to express genetic probes, and the expression of the luciferase reporter gene and eGFP reporter gene was controlled by the promoter of *Trem2*. Engineered THP‐1 cells were induced to differentiate into anti‐inflammatory macrophages in vitro and were treated with FDA‐approved drugs (**Figure** **7**A). After four rounds of drug screening, we identified ataluren and bortezomib among the 3069 compounds, both of which were subsequently validated to effectively inhibit TREM2 expression in engineered THP‐1‐derived macrophages (Figure 7B–F). Using a real‐time intelligent monitoring instrument, we observed that the addition of ataluren and bortezomib did not affect the growth of THP‐1‐derived macrophages (Figure 7G).

We subcutaneously injected an equal number of LLC cells (1 × 10^6^ cells per mouse) into C57BL/6N mice and administered intraperitoneal injections of ataluren (10 mg per kg per mouse) or bortezomib (0.5 mg per kg per mouse) on days 7, 10, 13, and 16 after tumor inoculation.^[^ ^24^, ^25^ ^]^ On day 19 after tumor inoculation, the mice were euthanized, and their tumor tissues and spleens were collected for further analysis (Figure 7H). The results showed that tumor weight and volume were significantly reduced in mice treated with either ataluren or bortezomib, and there was no significant difference in the body weight of the mice, indicating the safety of the screened drugs (Figure 7I,J; Figure S8A, Supporting Information). Further analysis of the spleens and tumor tissues of mice revealed that ataluren or bortezomib significantly reduced TREM2 expression in macrophages and increased the number of CD4^+^ T and NK cells, whereas the number of CD8^+^ T cells remained unchanged (Figure 7K; Figure S8B–E, Supporting Information). In addition, the production of CX3CL1 was significantly elevated in BMDMs from mice treated with ataluren or bortezomib (Figure 7L), whereas the expression of *Abca1* was significantly reduced (Figure 7M). What's more, we conducted in vivo experiments to observe whether ataluren or bortezomib could improve the survival of tumor‐bearing mice, which indicated extended survival in treated mice (Figure 7N). Finally, we used the established orthotopic lung adenocarcinoma mouse model to further validate that intraperitoneal administration of ataluren or bortezomib can effectively inhibit tumor growth and prolong the survival of mice (Figure 7O,P). Collectively, our findings suggest a potential therapeutic strategy for cancer that involves reshaping of the immune microenvironment.

## 3 Discussion

Increasing evidence highlights the high and specific expression of TREM2 within a subset of TAMs that suppresses immune response.^[^ ^8^, ^26^, ^27^, ^28^, ^29^ ^]^ TREM2 has been recognized as a marker for TAMs across various cancer types, indicating that TREM2^+^ TAMs may facilitate tumor growth, except in tumors of the central nervous system.^[^ ^30^, ^31^ ^]^ Given their abundance within the TME, TAMs are significant contributors to tumorigenesis and progression. Several studies have designated TREM2 as a potential target for immunotherapy of solid tumors.^[^ ^32^ ^]^ This prompted an investigation into the role of TREM2 in fostering tumor development and the possibility of using TREM2^+^ TAMs as therapeutic targets for lung cancer. Initially, our scRNA‐seq data indicated that TREM2 was specifically expressed in tumor‐infiltrating macrophages, and its expression was markedly elevated in tumor tissues compared to peritumoral tissues. Subsequently, to gain deeper insights into the role of TREM2 in the TME, we compared the scRNA‐seq data of tumor tissues from *Trem2* ^−/−^ and *Trem2* ^+/+^ mice. Our findings revealed that the deficiency of TREM2 led to an increase in macrophages with a pro‐inflammatory phenotype, accompanied by a significant increase in CD4^+^ T and NK cells, enhancing their capacity to counteract tumor growth.

Our study revealed the upregulation of CX3CL1 in TAMs following the deletion of TREM2. CX3CL1 is a member of the chemokine receptor superfamily,^[^ ^33^, ^34^ ^]^ and the specific receptor for CX3CL1 is CX3CR1, which is predominantly expressed on cytotoxic effector lymphocytes such as NK cells and cytotoxic T lymphocytes.^[^ ^35^ ^]^ CX3CL1 overexpression triggers recruitment of CX3CR1‐positive CD4^+^ T and NK cells.^[^ ^36^, ^37^ ^]^ miR‐561‐5p promotes pulmonary metastasis of hepatocellular carcinoma by suppressing CX3CL1, a chemokine essential for recruiting CX3CR1^+^ NK cells and activating their antitumor function.^[^ ^38^ ^]^ What's more, the CX3CR1 expression gradient is a key marker that delineates the functional heterogeneity in cytokine production and cytotoxic potential, and the differentiation states of CD4^+^ and CD8^+^ T cells, highlighting its promise for clinical translation.^[^ ^39^ ^]^ In this study, we found that the elevation of CX3CL1 induced by TREM2 deficiency did not obviously enhance the recruitment of CD8^+^ T cells. However, prior researches have revealed a distinct role of CX3CL1 in regulating CD8^+^ T cell function, demonstrating that CX3CL1 exerts a pivotal antitumor effect within liver tumor settings by facilitating the recruitment and activation of CD8^+^ T cells.^[^ ^40^, ^41^ ^]^

Adding further complexity to the role of CX3CL1 in tumors, another study reported that CX3CL1 was upregulated in tumors with GLI1 overexpression. In this context, elevated CX3CL1 did not contribute to antitumor immunity, instead, it promoted the recruitment of polymorphonuclear myeloid‐derived suppressor cells and suppressed T cell function, thereby facilitating tumor immune evasion.^[^ ^42^ ^]^ These contrasting observations collectively underscore the multifaceted and context‐dependent nature of the function of CX3CL1 in tumor biology. The existing controversy regarding its regulatory effects highlights the need for further in‐depth investigation to clarify the underlying mechanisms that dictate the pro‐ or anti‐tumor roles of CX3CL1 in different tumor contexts.

Beyond aiding CD8^+^ T cells in the direct identification and destruction of tumor cells, CD4^+^ T cells can also exert a direct anti‐tumor effect through the secretion of cytokines, including IFNγ, TNFα, and interleukin‐2 (IL‐2).^[^ ^43^, ^44^, ^45^ ^]^ CD4^+^ T cells constitute a diverse cell population, with the Th1 subset demonstrating a clear anti‐tumor function.^[^ ^46^ ^]^ Furthermore, CD4^+^ T cells collaborate with macrophages to induce an inflammatory form of cell death in tumor cells.^[^ ^47^ ^]^ NK cells can impede tumor growth via multiple pathways, such as the discharge of GZMB, direct killing of tumor cells through death receptor binding, and the emission of cytokines, such as IFNγ or TNFα, which directly target tumor cells to diminish their viability or rate of proliferation.^[^ ^48^ ^]^ Despite the presence of NK cells in primary solid tumors, metastases, and lymph nodes, NK cell infiltration is often limited to most solid tumors.^[^ ^49^, ^50^, ^51^ ^]^ Promoting the recruitment of NK cells to tumor tissue sites is an effective way to inhibit tumor progression.^[^ ^52^ ^]^ Our study revealed that genetic deletion of TREM2 in TAMs rescued NK cell accumulation by secreting more CX3CL1 and promoting regression of lung tumors. However, the complex interplay between TREM2‐deficient TAMs, CD4^+^ T cells, and NK cells in their anti‐tumor effects warrants further investigation.

Although TREM2 blockade can enhance the response of CD8^+^ T cells, slow tumor progression, and synergize with immune checkpoint inhibitors in models of sarcoma, ovarian cancer, and breast cancer,^[^ ^27^, ^53^ ^]^ our study presents different findings. In this study, scRNA‐seq data indicated no significant difference in CD8^+^ T cell infiltration between the tumor tissues of *Trem2* ^+/+^ and *Trem2* ^‐/‐^ mice. In line with our study, Park et al. demonstrated that in *Trem2* KO mice, CD8^+^ T cell depletion only partly reversed the reduced tumor burden in tumor‐bearing ones, whereas NK cell depletion fully reversed the reduced tumor growth phenotype.^[^ ^52^ ^]^ We believe that CD8^+^ T cell depletion did not affect tumor volume because their numbers and function within the tumor microenvironment are likely restricted, as factors such as fibrosis and metabolic suppression can impair their function.^[^ ^54^ ^]^ In addition, the low expression of MHC‐I molecules on tumor cells likely impedes the effective recognition and elimination of tumor cells by CD8^+^ T cells.^[^ ^55^, ^56^ ^]^ Thus, in instances of low MHC‐I expression by tumor cells, the role of immune cells that can target and kill tumor cells independently of MHC‐I is especially significant, including CD4^+^ T and NK cells.^[^ ^57^ ^]^

Macrophages are crucial components of the TME and their metabolic state significantly influences their functionality.^[^ ^58^, ^59^ ^]^ Interest in the role of cholesterol metabolism in tumorigenesis has recently increased. Our scRNA‐seq data indicated that ABCA1, a crucial lipid transport protein in TAMs, was significantly downregulated upon TREM2 deletion, leading to the programming of TAMs toward a pro‐inflammatory phenotype. The loss of ABCA1, which is responsible for cholesterol efflux, reverses the tumor‐promoting function of TAMs and reduces tumor progression.^[^ ^10^ ^]^ TREM2 has also been implicated in the regulation of cholesterol metabolism in microglia.^[^ ^60^ ^]^

Our study found that the loss of TREM2 inhibited ABCA1 expression to promote cholesterol accumulation in macrophages and induced the programming of TAMs into a pro‐inflammatory phenotype characterized by increased production of the pro‐inflammatory factor CX3CL1, which further recruited more CD4^+^ T and NK cells and enhanced anti‐tumor immunity. The findings from our scRNA‐seq analysis of *Trem2* ^+/+^ and *Trem2* ^−/−^ mice showed a notable increase in CX3CR1 expression, specifically in CD4^+^ T and NK cells in *Trem2* ^−/−^ mice, which exceeded the expression levels detected in CD8^+^ T cells. This observation elucidates the mechanism underlying the anti‐tumor effects of TREM2 deficiency, which are not dependent on CD8^+^ T cells.

The immunosuppressive effect of TREM2 on macrophages suggests that TREM2 is a viable target, and that drugs targeting TREM2 can inhibit tumor progression.^[^ ^61^ ^]^ We identified ataluren and bortezomib, two small‐molecule inhibitors that reduce TREM2 expression, with the potential to inhibit tumor growth and improve survival in a lung cancer model. Ataluren, a translational read‐through induction drug, has been approved to treat Duchenne muscular dystrophy.^[^ ^62^ ^]^ Bortezomib combined with lenalidomide and dexamethasone (VRd) is the first‐line treatment for newly diagnosed multiple myeloma.^[^ ^63^ ^]^ We are the first to report that both drugs can inhibit TREM2 expression in TAMs, suggesting that their potential to improve clinical outcomes of patients with cancer. It should be emphasized that the combination of targeting TREM2 drugs with immunotherapy or other therapies merits further exploration.

We propose that TREM2 on TAMs is an important immune checkpoint that suppresses macrophage pro‐inflammation by regulating cholesterol metabolism and dampening anti‐tumor immunity mediated by NK and CD4^+^ T cells. Therefore, TREM2 inhibition enhances the anti‐tumor response by reshaping the immune microenvironment, underscoring a potential novel therapeutic strategy for cancer treatment.

### 3.1 Limitations of the Study

There are some limitations to this study. After observing the phenomenon that cholesterol accumulation in macrophages leads to increased secretion of CX3CL1, we did not conduct in‐depth research into its specific mechanism, which warrants further investigations. In our study, we demonstrated that ataluren and bortezomib effectively inhibit TREM2 expression in macrophages; however, we did not investigate their mechanisms underlying this inhibition. In addition, the drugs used in this study may lack sufficient potency at current doses and regimens to significantly improve survival. The limited survival benefit could also stem from the small sample size and individual variations among the mice.

## 4 Experimental Section

### Human Samples

Tumor tissues and matched peritumor tissues were collected from 10 patients diagnosed with NSCLC. Freshly resected tissues were placed in phosphate‐buffered saline (PBS) and immediately transported to the laboratory for further analysis. All participants enrolled in this study provided written informed consent, and the study was approved by the Committee of the First Affiliated Hospital of Zhengzhou University.

### Murine Tumor Models

All animal experiments were reviewed and approved by the Institutional Animal Care and Use Committee of the First Affiliated Hospital of the Zhengzhou University. Wild‐type (WT) C57BL/6N and C57BL/6N *Trem2* ^−/−^ mice were generated by Cyagen Bioscience, Inc. (Suzhou, China). All mice were bred and maintained in a specific pathogen‐free (SPF) facility in standard cages at 22 °C under a 12 h light‐dark cycle and received food and water. All fodders were replaced weekly, with regular health monitoring.

### Orthotopic Lung Cancer Mouse Models

To establish orthotopic lung cancer mouse models, after intraperitoneal anesthesia induction with ready‐to‐use tribromoethanol solution (Nanjing Aibei Biotechnology, M2910), mice were positioned in dorsal recumbency on a thermostatically controlled heating pad (37 °C). The dorsal fur was shaved, and the skin was disinfected with iodine tincture. Guided by the superior margin of the fifth rib, an equal volume of luciferase‐tagged LLC cells (5 × 10⁵ cells per mouse) was then injected into the lung tissue. Tumor formation was confirmed at defined intervals via bioluminescence imaging using an IVIS imaging system.

### Single‐Cell RNA‐seq Library Construction and Sequencing

Tumors from *Trem2* ^+/+^ and *Trem2* ^−/−^ mice were washed in ice‐cold RPMI 1640 medium and dissociated using collagenase IV, Dispase, and DNase I. After counting, fresh cells were washed twice with RPMI 1640 and resuspended at a density of 1 × 10^6^ cells mL^−1^ in phosphate‐buffered saline (PBS) containing 0.04% bovine serum albumin. Single‐cell RNA‐seq libraries were prepared using a SeekOne Digital Droplet Single Cell 3 library preparation kit (SeekGene Catalog No. K00202). Briefly, an appropriate number of cells were mixed with reverse transcription reagent and added to the sample well in the SeekOne chip S3. Subsequently, Barcoded Hydrogel Beads and partitioned oil were dispensed separately into the corresponding wells of the chip. After emulsion droplet generation, reverse transcription was performed at 42 °C for 90 min followed by inactivation at 85 °C for 5 min. Next, the cDNA was purified from the broken droplets and amplified by PCR. The amplified cDNA product was purified, fragmented, end‐repaired, A‐tailed, and ligated to a sequencing adaptor. Finally, indexed PCR was performed to amplify the DNA representing the 3′ polyA part of the expressing genes, which also contained the Cell Barcode and Unique Molecular Index. The indexed sequencing libraries were cleaned using VAHTS DNA Clean Beads (Vazyme, N411‐01) and analyzed using a Qubit (Thermo Fisher Scientific, Q33226) and a Bio‐Fragment Analyzer (Bioptic, Qsep400). The libraries were then sequenced on an Illumina NovaSeq 6000 with a PE150 read length, or on a DNBSEQ‐T7 platform with a PE150 read length.

### Analysis of scRNA‐seq Data

Raw sequencing data were processed using Fastp (v.0.20.1) to trim the primer sequences and eliminate low‐quality bases. Subsequently, SeekOne Tools were used to preprocess the sequencing data and align them with GRCm38 to obtain a gene expression matrix. Seurat (version 4.3.0.1)^[^ ^64^ ^]^ was used for filtering and data normalization such that cells with < 200 or > 5000 detected genes were omitted. The MAD variance normal^[^ ^65^ ^]^ was used to remove cells affected by the mitochondrial genes. The remaining cells were used for subsequent analysis.

### Antibody‐Mediated Blockade and Depletion Studies

To deplete CD4^+^ and CD8^+^ T and NK cells, mice were intraperitoneally injected with anti‐CD4 (Selleck, clone GK1.5, A2101), anti‐CD8α (Selleck, clone GK2.43, A2102), anti‐NK1.1 (BioXCell, clone PK136, BE0036), or IgG isotype control (Selleck, A2150). On days ‐2, 0, 4, and 8 after tumor inoculation, mice were administered anti‐CD4, anti‐CD8 antibody, or IgG isotype control (200 µg per mouse) intraperitoneally (i.p.). On days 0, 3, 7, and 10 after tumor inoculation, mice were intraperitoneally (i.p.) administered the anti‐NK antibody or IgG isotype control (100 µg per mouse). All mice were sacrificed on day 17 after tumor inoculation.

To block CX3CR1, mice were intraperitoneally injected with CX3CR1 inhibitor (JMS‐17‐2, S0135, Selleck) at a dose of 10 mg per kg per mouse or solvent (5% DMSO+45% PEG300+50% ddH2O) every day for two weeks after the day of tumor inoculation. All mice were sacrificed on day 16 post tumor inoculation.

### THP‐1 Monocytes Differentiation to Anti‐Inflammatory Macrophages In Vitro

THP‐1 cells were obtained from the Cell Bank and the Stem Cell Bank of the Chinese Academy of Sciences. The cells were cultured in RPMI‐1640 medium (Sigma, R8758) supplemented with 10% fetal bovine serum, 100 U mL^−1^ penicillin, and 100 µg mL^−1^ streptomycin at 37 °C with 5% carbon dioxide. For differentiation, THP‐1 cells were seeded at a density of 1 × 10^6^ cells mL^−1^ in a 12‐well plate, and treated with 100 ng mL^−1^ PMA (Sigma, 79346) for 24 h to induce macrophage‐like differentiation. And then the medium was replaced with RPMI‐1640 medium without PMA, and the cells were cultured for an additional 48 h. To induce anti‐inflammatory macrophages, the cells were further treated with RPMI‐1640 medium containing 40 ng mL^−1^ IL‐4 (Pepro Tech, 200‐04) for an additional 24 h and collected for further experiments.

### Generation of Tumor‐Associated Macrophages (TAMs) with Tumor‐Conditioned Media (TCM)

To prepare TCM, A549 cells were grown to ≈80% confluence, and serum‐free DMEM medium (Sigma, D6429) was added to the culture and incubated for 48 h. The TCM was subsequently collected and filtered through a 0.22µm filter to remove any cellular debris or particles, ensuring its purity for subsequent experiments and applications. THP‐1 monocytes were inoculated in a 12‐well plate at a density of 1 × 10^6^ cells mL^−1^. The cells were then cultured in a 1:1 ratio of RPMI and TCM, with half of the medium replenished daily. The cells were collected on day three post‐inoculation.

### Isolation of Human Peripheral Blood Mononuclear Cells (PBMCs)

PBMCs were isolated from the buffy coats of healthy donors at the First Affiliated Hospital of Zhengzhou University. Density gradient centrifugation was performed using lymphocyte isolation solution (TBD, LTS1077) at 20 °C (2500 rpm) for 25 min with an acceleration and deceleration of 9. Purified PBMCs were resuspended in MACS Running Buffer (Miltenyi, 130‐091‐221).

### Isolation and Culture of Bone Marrow‐Derived Macrophages (BMDMs)

Femurs were dissected from the muscle tissue and then washed in PBS. Flushed the bone marrow cells from the bones using PBS under aseptic conditions. Collected the flushed cells in a sterile 1.5 mL conical tube and centrifuged the cell suspension at 1600 rpm for 5 min at 4 °C. Discard the supernatant. Resuspended the cell pellet in 2–5 mL of RBC lysis buffer and incubated for 8 min at room temperature. Added excess complete DMEM medium to stop the lysis reaction. Centrifuged at 1600 rpm for 5 min at 4 °C and discarded the supernatant. Resuspended the cell pellet in complete DMEM medium. Counted the cells and seeded the bone marrow cells into a 12‐well plate at a concentration of 1 × 10^6^ cells mL^−1^ and cultured in DMEM (Sigma, D6429) (37 °C, 5% CO_2_) supplemented with 10 ng mL^−1^ of recombinant murine M‐CSF (Pepro Tech, 315‐02). The first day of plating was considered as day 0. Half of the medium and 10 ng mL^−1^ of recombinant murine M‐CSF were replaced on day 3. In addition, the medium was replaced, and 10 ng mL^−1^ murine IL‐4 (Pepro Tech, 214‐14) and 10ng mL^−1^ murine IL‐13 (Pepro Tech, 210‐13) were simultaneously added on day 5 to induce anti‐inflammatory macrophages. Cells were collected on day 6 for subsequent experiments.

### Western Blot

The collected cell samples were centrifuged at 1800 rpm for 5 min, after which the supernatant was carefully discarded. The cell pellets were then resuspended in RIPA lysis buffer (Beyotime, P0013B) in the presence of protease and phosphatase inhibitors and lysed on ice for 10 min before being stored at −80 °C. After that, the protein samples were centrifuged at 12000 rpm for 5 min. The supernatant containing the soluble proteins was mixed with sodium dodecyl sulfate (SDS) loading buffer at a volume ratio of 1/4 (SDS to supernatant) and denatured at 100 °C for 10 min. Protein peptides were separated by SDS‐PAGE (DAKEWE, 8012011) and transferred to a nitrocellulose membrane (Cytiva, 1060001). The membrane was blocked with NcmBlot blocking buffer (NCM Biotech, p30500) for 15 min and incubated overnight with the primary antibodies. The membranes were washed with TBST for 3 times the following day and incubated with horseradish peroxidase‐conjugated secondary antibodies (1:5000). Blots were developed using an ECL Substrate (Tanon, 180‐501) and visualized using a BLT Imaging system. β‐actin was used as loading controls, depending on the mass of the proteins of interest. All western blot experiments were performed with three biological replicates. **Table** **1**

**Table 1:** Western blot antibody reagents.

Antibody	Source	Cat.	Dilution
TREM2	Proteintech	68723	1:2000
ABCA1	ABclonal	A22125	1:500
CX3CL1	Affinity	DF12376	1:1000
β‐actin	Affinity	T0022	1:15000
Anti‐rabbit HRP 2nd antibody	Solarbio	111‐035	1:5000
Anti‐mouse HRP 2nd antibody	Solarbio	115‐035	1:5000

### RT‐qPCR

Total RNA was extracted using TRIzol (Takara, 9109) following a well‐established protocol, and the RNA concentration and purity were assessed using a Nanodrop 2000 spectrophotometer (Thermo Fisher, USA). Complementary DNA was synthesized with 1 µg of RNA using an All‐in‐one RT kit (Vazyme, R333‐01) according to the manufacturer's instructions. Quantitative qPCR analysis was performed with diluted cDNA, SYBR Green PCR Master Mix (Vazyme, Q712), and corresponding primers using a C1000 Touch Thermal Cycler (Bio‐Rad). The specificity of the amplification products was confirmed using melting curve analysis. The PCR reactions for each gene were repeated 3 times. Independent experiments were conducted in triplicate. For qPCR data analysis, the gene expression level was normalized to that of GAPDH, and the relative expression level was calculated using the 2‐ΔΔCt method. **Table** **2**

**Table 2:** Primer sequence of RT‐qPCR assay.

Gene (mice)	Forward primer sequence (5′–3′)	Reverse primer sequence (3′–5′)
*Gapdh*	GGTTGTCTCCTGCGACTTCA	TGGTCCAGGGTTTCTTACTCC
*Trem2*	CTGGAACCGTCACCATCACTC	CGAAACTCGATGACTCCTCGG
*Abca1*	GCTTGTTGGCCTCAGTTAAGG	GTAGCTCAGGCGTACAGAGAT
*Cx3cl1*	AAATGCGAAATCATGTGCGAC	CCTGGTTTAGCTGATAGCGGAT

Gene (human)	Forward primer sequence (5′–3′)	Reverse primer sequence (3′–5′)
*Gapdh*	CAGGAGGCATTGCTGATGAT	GAAGGCTGGGGCTCATTT
*Trem2*	GCCTGCATACTTGCCACTT	ACCTCCCCACTCCCTCA
*Abca1*	GAAGTACATCAGAACATGGGC	GATCAAAGCCATGGCTGTAG
*Cx3cl1*	ACCACGGTGTGACGAAATG	TGTTGATAGTGGATGAGCAAAGC

### Enzyme‐Linked Immunosorbent Assay (ELISA)

Supernatants were collected from BMDM of both *Trem2* ^+/+^ and *Trem2* ^−/−^ mice as well as from BMDM treated with ataluren and bortezomib. CX3CL1 levels were measured using a mouse CX3CL1 ELISA kit (Elabscience, E‐EL‐M0267) according to the manufacturer's instructions. Supernatants were collected from CD14^+^ monocyte‐derived macrophages under three conditions: treated with MβCD, cholesterol, and left untreated as a control. Additionally, supernatants were also collected from macrophages treated with a series of increasing concentrations of cholesterol. CX3CL1 levels were measured using a human CX3CL1 ELISA kit (Elabscience, E‐EL‐H0044) according to the manufacturer's instructions.

### Immunofluorescence

Immunofluorescence was performed to detect TREM2 in tumor and peritumoral tissues from NSCLC patients, CX3CL1 in tumor tissues from *Trem2* ^+/+^ and *Trem2* ^−/−^ mice, and ABCA1 in BMDMs from *Trem2* ^+/+^ and *Trem2* ^−/−^ mice. For organizational immunofluorescence, samples were fixed by immersion in 4% paraformaldehyde (PFA) at 4 °C for 24 h, embedded in paraffin, and sliced to a thickness of 3 µm. For cellular immunofluorescence, bone marrow cells were seeded at a concentration of 1 × 10^6^ cells mL^−1^ into a 24‐well plate with corresponding cell slides (abs7027, Absin) and then induced into anti‐inflammatory macrophages based on a previously described method. The cell‐coated coverslips were then carefully inverted onto the glass slides and blocked with 5% BSA at room temperature for 30 min. All the above samples were incubated with the following primary antibodies (rabbit anti‐TREM2 (MAB17291, R&D), rabbit anti‐CD163 (ab182422, Abcam), rabbit anti‐CD68 (ab192847, Abcam), rabbit anti‐CX3CL1 (DF12376, Affinity), rabbit anti‐ABCA1 (A21976, Abclonal), and rabbit anti‐F4/80 (#70076, CST)) overnight at 4 °C, followed by incubation with the corresponding secondary antibodies for 1 h at room temperature the next day, and then stained with DAPI (G1012, Servicebio) for 10 min. Finally, the slides were covered with an anti‐fluorescence quenching reagent (G1401; Servicebio). The samples were visualized using a standing fluorescence microscope (Nikon, E100, Japan) and Pannoramic DESK scanner (3DHISTECH, Hungary) and processed using Case viewer software (v.2.0, 3DHISTECH, Hungary).

### Filipin Staining

THP‐1 cells were seeded at a concentration of 1 × 10^6^ cells mL^−1^ into a 24‐well plate with corresponding cell slides (abs7027, Absin) and then induced into tumor‐associated macrophages with A549 CM. The slides were washed three times with PBS, followed by incubation for 1 h in the dark at room temperature with 50 µg mL^−1^ Filipin III (SAE0087, Sigma). After this, slides were washed with PBS again and coverslipped with anti‐fluorescence quenching reagent (G1401; Servicebio). The fluorescence signals of Filipin were detected by confocal microscope (LSM880, Zeiss) and the same settings were employed between different groups.

### Immunohistochemical

Tumor tissues from *Trem2* ^+/+^ and *Trem2* ^−/−^ mice were collected and fixed in 4% buffered formalin, dehydrated in increasingly dilute alcohol solutions, embedded in paraffin wax, and cut into 3‐µm‐thick sections for H&E staining. Anti‐CD49b (1:200, A19068; Abclonal), anti‐CD4 (1:100, GB11064, Servicebio), anti‐CX3CR1 (1:1000, GB11861, Servicebio) and NKp44 (1:500, GB11615, Servicebio) primary antibodies were used. Sections and pictures were observed and captured under a standing fluorescence microscope (Nikon, E100, Japan) and Pannoramic DESK scanner (3DHISTECH, Hungary) and processed using Case Viewer software (v.2.0, 3DHISTECH, Hungary).

### Flow Cytometry Analysis

Flow cytometry was used to determine the expression levels of cell surface markers and internal factors. Single cells were labeled with antibodies against surface molecules for 15 min at 4 °C in the dark. For intracellular cytokine detection, cells were first fixed with a fixative (Servicebio, G1101) on ice for 30 min in the dark, followed by 30 min of exposure to 1× Intracellular Staining Perm Wash Buffer (BioLegend, 421002), and then labeled with antibodies for 15 min at 4 °C in the dark. For gating strategies, dead cells were labeled with a Fixable Viability Dye (BioLegend, 423101) and excluded. Flow cytometry was performed using a Beckman CytoFLEX flow cytometer, and the data were analyzed using the CytExpert software (v.2.0.0.283, Beckman Coulter) and FlowJo software (v.10.8.1, BD). The antibodies used for cellular staining are listed in Table S3 (Supporting Information).

### Cholesterol Quantification

Cells were first washed and resuspended in PBS, and then lysed by ultrasonication to release cholesterol, followed by high‐speed centrifugation (12 000 rpm, 15 min) to obtain the supernatant. Standards and samples were diluted appropriately according to the manufacturer's guidelines, and Amplex Red reagent working solution (Thermo Fisher, A12216) was added to initiate the reaction. After incubation at 37 °C in the dark for 30 min or longer, the fluorescence intensity was measured at an emission wavelength of 590 nm (SpectraMax id3 plate reader) to quantify cholesterol levels. The intracellular cholesterol content was calculated by comparison with a standard curve.

### Cholesterol Efflux Assay

THP‐1 cells were differentiated into TAMs with A549 CM as previously described and incubated with 5 µm NBD‐cholesterol (J&K Scientific, 62533) for 4h. After removing the supernatant and washing twice with phenol red‐free RPMI, the cells were treated with 50µg mL^−1^ of APOA (MCE, HY‐P7526) for 4h to induce cholesterol efflux. The supernatant representing cholesterol‐induced efflux fluid (A) was collected. The cells were then lysed with 0.1% Triton X‐100 (Solarbio, T8200) and centrifuged at 12000 rpm for 10 min to remove debris. The supernatant was obtained as the intracellular cholesterol solution (B). One hundred microliters of both A and B were transferred to a 96‐well plate, with triplicate wells for each sample. Fluorescence was measured at excitation and emission wavelengths of 469 and 537 nm, respectively, using a SpectraMax id3 plate reader. The formula for the cholesterol efflux rate is as follows:

(1) $$\begin{array}{ccc} & & \mathrm{Cholesterol}\;\mathrm{Efflux}\;\mathrm{Rate}\\ & & =\frac{\mathrm{cholesterol}-\mathrm{induced}\;\mathrm{efflux}\;\mathrm{fluid}}{\mathrm{cholesterol}-\mathrm{induced}\;\mathrm{efflux}\;\mathrm{fluid}+\mathrm{intracellular}\;\mathrm{cholesterol}\;\mathrm{solution}} \end{array}$$

### Plasmid Construction and Stable Transfection

shTREM2 plasmid (Shanghai GeneChem Co. Ltd.) were efficiently transduced into competent DH5 cells (Beijing Dingguo Changsheng Biotechnology Co. Ltd.) using a combined chemical heat‐shock transformation method. Following this, bacterial amplification was performed, and the bacterial culture was collected. The shTREM2 plasmid was extracted using the EndoFree Mini Plasmid Kit II (TIANGEN, DP118) following the manufacturer's protocol. The plasmid transfection reagent complex was co‐transfected into 293T cells with jetPRIME (Polyplus, 101000046), and incubated at room temperature for 15 min. After 4 h of transfection, the medium was replaced with fresh DMEM. The virus‐containing supernatant was harvested 48–72 h post‐transfection, centrifuged to remove debris, and applied to precultured THP‐1 cells. Successfully transfected cells were selected using 2 µg mL^−1^ puromycin (Biosharp, BL528A), and the efficiency of TREM2 gene knockout was confirmed by qRT‐PCR **Table** **3**.

**Table 3:** shRNA sequences used in gene knockdown.

ID	Target (sense) sequence (5′ to 3′)
TREM2‐RNAi(83271‐1)	gcGTGTGGTCAGCACGCACAA

### Drug Screening

To identify drugs that reduce TREM2 expression, an FDA‐approved drug library was purchased for drug screening (Selleck, L1300). A lentiviral vector encoding the *Trem2* promoter‐driven luciferase‐eGFP reporter gene was used to generate a stable transgenic THP‐1 cell line. Flow cytometry‐based sorting refined the population of THP‐1 cells expressing *the Trem2* promoter‐luciferase‐eGFP. Cells (5 × 10^4^ cells well^−1^) were seeded in a 96‐well plate in vitro and induced to differentiate into anti‐inflammatory macrophages with PMA and IL‐4, according to the aforementioned method. The cells were then treated with each compound for 24 h, and fluorescence readings were collected using an automatic microplate reader (Molecular Devices). A total of four rounds of drug screening experiments were conducted. Compounds that showed an OD value lower than that of the untreated control group were selected for the next round of drug screening experiments. The drug concentrations used in the four rounds of screening experiments were progressively reduced, namely 10 µm, 5 µm, 500 nm, and 100 nm. Ataluren and bortezomib were the most suitable candidate compounds.

### Transwell Assay

The chemotactic effects of varying concentrations of recombinant human CX3CL1 (rhCX3CL1) (MCE, HY‐P7180) on PBMC, CD4^+^, CD8^+^ T, and CD56^+^ NK cell subsets were investigated. Cell migration assays were performed using Transwell cell culture inserts comprising two chambers separated by an 8.0 µm polycarbonate membrane (Corning, 3422). The lower chamber was filled with serum‐free RPMI or THP‐1 shTREM2 conditioned medium, supplemented with varying concentrations of rhCX3CL1, with the option to pre‐treat with CX3CL1 antibodies (R&D, MAB275) for 2 h. PBMC or purified CD4^+^, CD8^+^ T and CD56^+^ NK cell subsets (1 × 10^5^ cells in 200 µL) were added to the upper chamber and incubated for 12 h. Migrated cells in the lower chamber were quantified by microscopy or analyzed by flow cytometry to determine the types and proportions of migrated cells.

### Cell Growth Monitoring

To evaluate the safety of bortezomib and ataluren on THP‐1‐derived macrophages, a real‐time intelligent monitoring instrument (CM100‐α; Shanghai Six Beans Medical Technology Co., Ltd., Shanghai, China) was employed to assess growth by measuring the cell index of THP‐1 cells. THP‐1 cells were first seeded in a 16‐well electrode plate at a density of 4 × 10⁴ cells per well and induced to adhere using 100 ng mL^−1^ PMA for 4 h. The cells were then treated with bortezomib (100 nm) and ataluren (100 nm). Cell signals were detected every 10 min throughout the assay, which was terminated at 56 h for statistical analysis.

### Study on Mouse Survival

Following the NIH Guidelines for Endpoints in Animal Study Proposals (2019), during the mouse survival observation period, the surface tumor volume was measured every 2 days. The following criteria were used as experimental endpoints: 1) Tumor size, including any single tumor diameter exceeding 20 mm or tumor volume surpassing 2000 mm^3^; 2) tumor condition, such as ulceration, necrosis, or infection of the tumor; and 3) health deterioration, such as the occurrence of hypothermia or seizure in mice.

### Statistical Analysis

Data from different groups were compared using the two‐tailed Student's *t*‐test, one‐way analysis of variance (ANOVA), or two‐way ANOVA. All experiments were repeated at least three times. Survival data were analyzed using the Kaplan–Meier survival curve time series test. Statistical analyses were performed using the GraphPad Prism 9.5.1 software. Data were presented as means ± standard error of mean (SEM). ns: not significant, ^*^ *p <* 0.05, ^**^ *p <* 0.01, ^***^ *p <* 0.005, ^****^ *p <* 0.0001.

## Conflict of Interest

The authors declare no conflict of interest.

## Author Contributions

Y.W., W.Y., and X.W. are co‐first authors. L.Y. and Y.Z. contributed to conceptualization. W.Y. contributed to methodology. Y.W., W.Y., X.W., Q.Z., Q.L., A.L., S.L., and T.W. contributed to Investigation. Q.Z. and T.W. contributed to data Curation., Y.W., W.Y., and X.W. contributed to formal analysis. Y.W. and X.W. were responsible for writing the original draft., L.Y. and Y.Z. were responsible for writing, reviewing, and editing. L.Y. and Y.Z. provided funding acquisition., Y.W., W.Y., and X.W. contributed to visualization.

## Supporting information



Supporting Information

Supporting Information

Supporting Information

Supporting Information

## Data Availability

scRNA‐sequencing datasets have been deposited in the Genome Sequence Archive (GSA) (https://ngdc.cncb.ac.cn/gsa/). Human data are accessible through GSA‐human: HRA006716, and HRA009508. Mouse data are accessible through GSA: CRA020898. These data are publicly available as of the date of publication. All data reported in this paper will be shared by the corresponding author.
