# Supplementary material for: TREM2‐Mediated Cholesterol Efflux in Macrophages Inhibits Anti‐Tumor Immunity via Limitation of CD4+ T and NK Cells
Source: Adv Sci (Weinh). 2025 Oct 20;13(5):e06995. doi: 10.1002/advs.202506995 (PMC12850164; doi:10.1002/advs.202506995)
Supplement: Supplementary file 1 — Supporting Information [file ADVS-13-e06995-s002.docx]

**Supplementary figures**

**
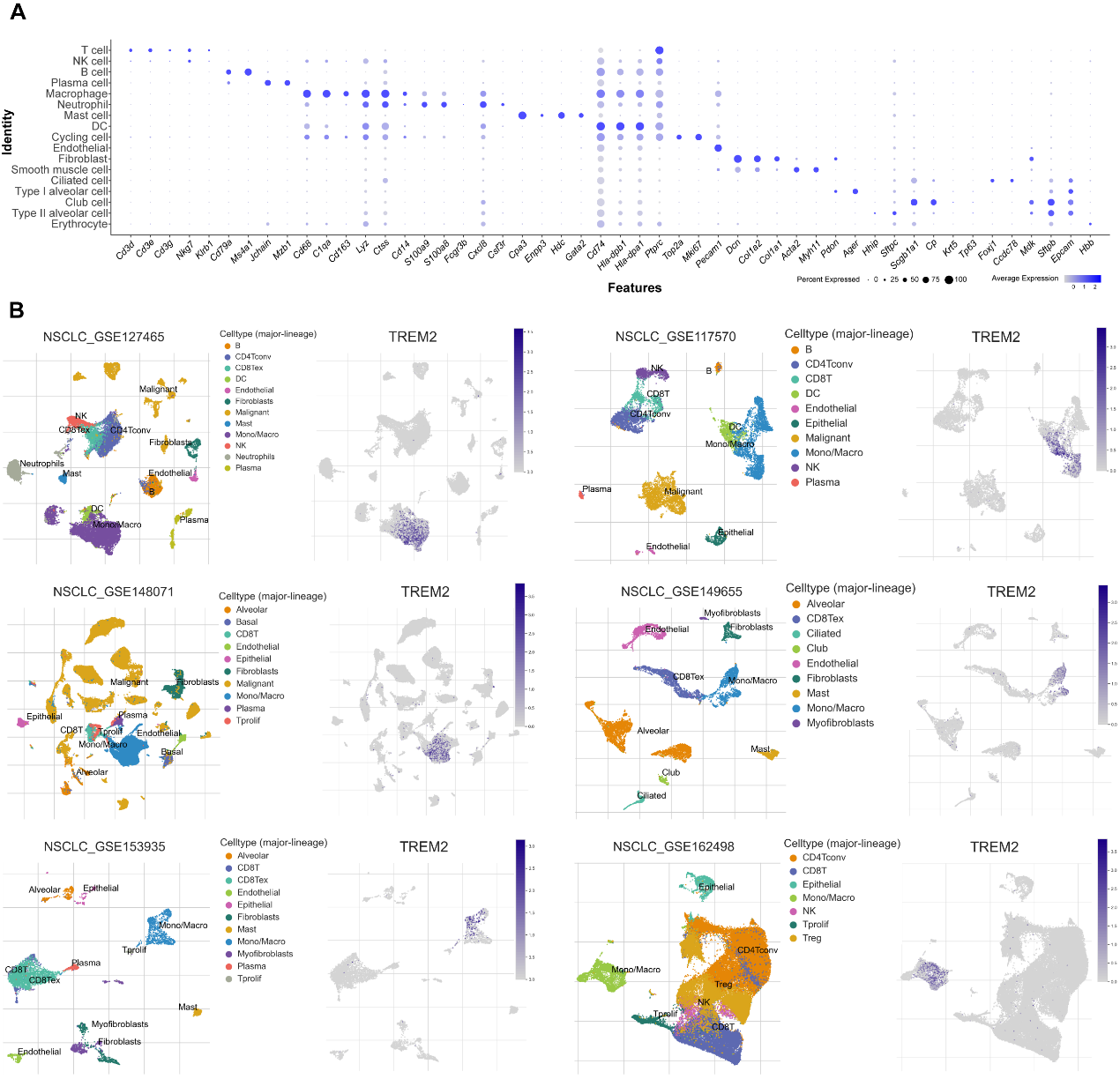
**

**Supplementary Figure 1. TREM2 exhibits high specific expression in macrophage.**

(A) Expression patterns of representative 17 cell clusters-specific marker genes from 10 paired tumor and peritumor tissues of NSCLC on UMAP. Dot diameter indicates the proportion of cluster cells expressing a given gene. The color intensity represents the gene expression levels. (B) Analysis of *Trem2* gene expression in NSCLC using scRNA-seq dataset from the Tumor Immune Single-Cell Hub database.

**
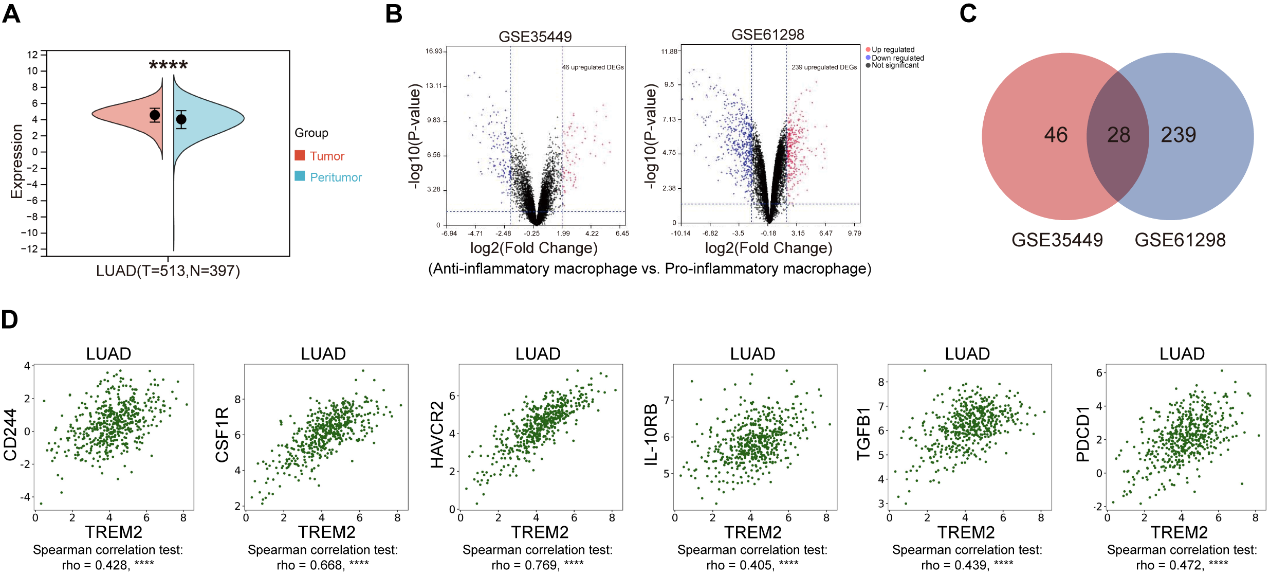
**

**Supplementary Figure 2. TREM2 is associated with immunosuppression.**

(A) Bean plot showing the expression of TREM2 on macrophages in paired tumor and peritumor tissues of LUAD analyzed from TCGA database. (B) Volcano plots showing the DEGs between anti-inflammatory and pro-inflammatory macrophages from the GSE35449 (left) and GSE61298 (right) datasets (criteria: P<0.05 and absolute log2 [fold change] ≧2). (C) Venn diagram showing the overlap of upregulated genes in anti-inflammatory macrophages between the GSE35449 and GSE61298 datasets. (D) Scatter plots depicting the correlation between TREM2 expression and immunosuppressive molecules in the LUAD cohort from the TCGA database. “rho” denotes the Spearman correlation coefficient. ****P < 0.0001.


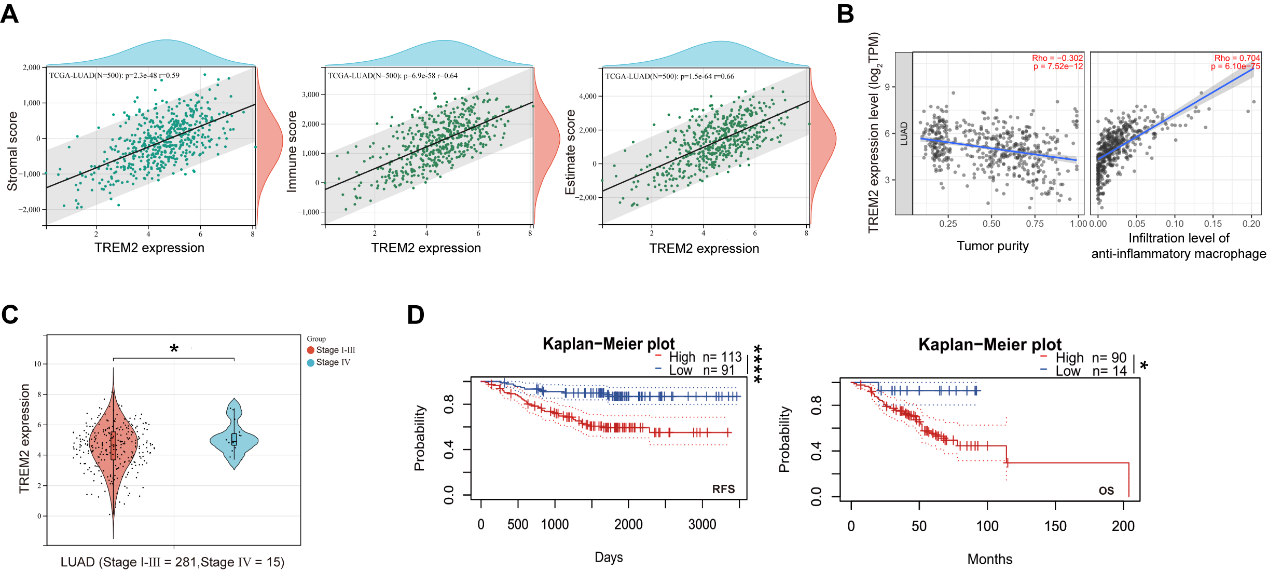


**Supplementary Figure 3. High expression of TREM2 is associated with poor prognosis.**

(A) Scatter plots between TREM2 expression and Stromal, Immune, ESTIMATE scores in TCGA LUAD dataset. (B) Scatter plots between TREM2 expression and tumor purity and infiltration level of anti-inflammatory macrophages in TCGA LUAD dataset using the xCell method. (C) Violin plot showing TREM2 expression across various tumor stages in the TCGA LUAD dataset. (D) Kaplan-Meier plots of TREM2 expression and overall survival (OS), and recurrence-free survival (RFS) from the Prognoscan database. *P < 0.05; ****P < 0.0001.

**
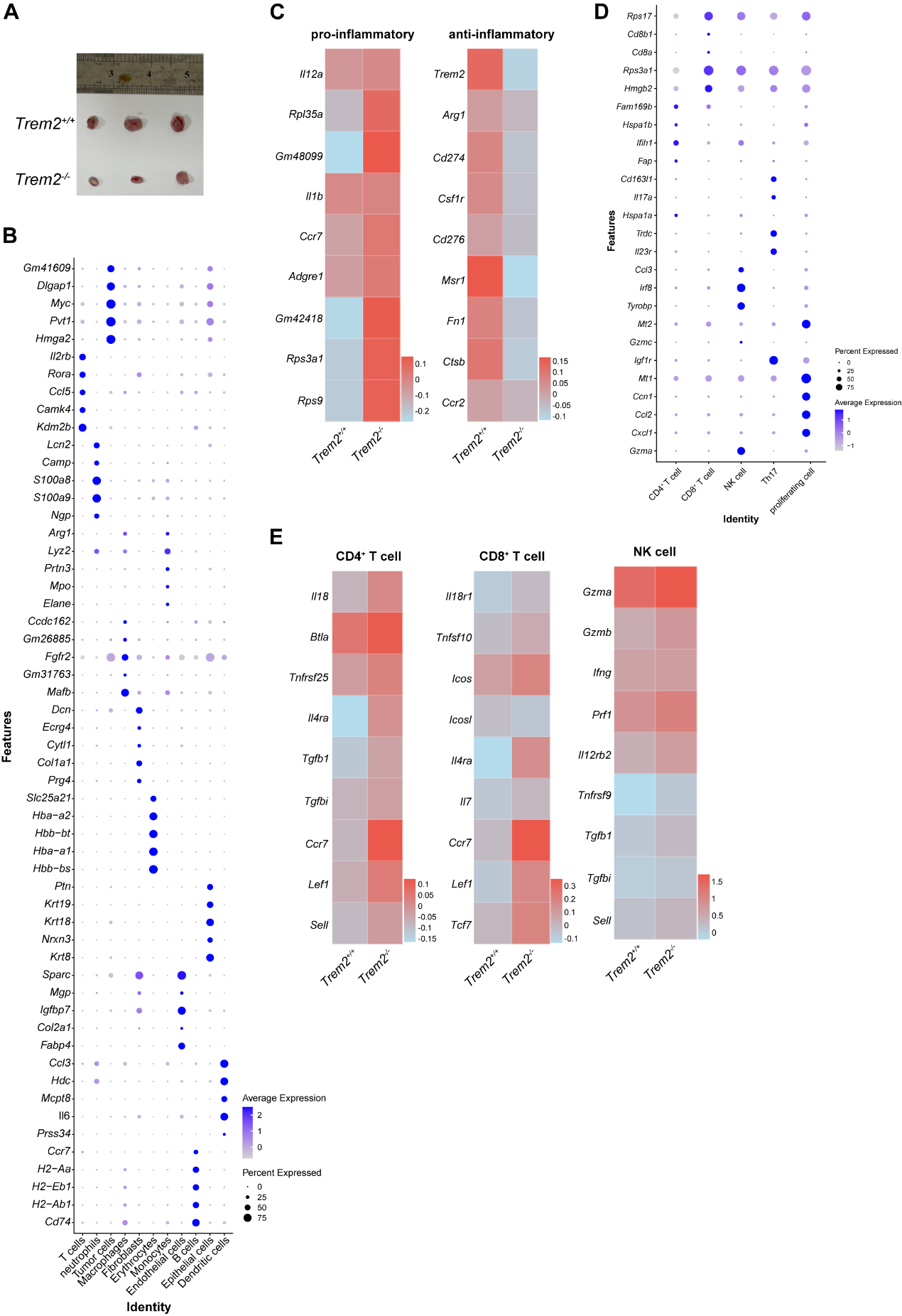
**

**Supplementary Figure 4. The absence of TREM2 reshaped the tumor microenvironment towards pro-inflammatory phenotype.**

(A) Tumor images of *Trem2*^+/+^ and *Trem2*^-/-^ mice 10 days after tumor cells implantation (n = 3 /group). (B) Expression patterns of representative cluster-specific marker genes in tumor tissues of *Trem2*^+/+^ and *Trem2*^-/-^ mice on UMAP. Dot diameter indicates the proportion of cluster cells expressing a given gene. The color intensity represents the gene expression levels. (C) Heatmaps showing the gene expression patterns of pro-inflammatory macrophage (left) and anti-inflammatory macrophage (right) from the scRNA-seq data of tumor tissues of *Trem2*^+/+^ and *Trem2*^-/-^ mice. (D) Expression patterns of representative cluster-specific marker genes in tumor tissues of *Trem2*^+/+^ and *Trem2*^-/-^ mice on UMAP. Dot diameter indicates the proportion of cluster cells expressing a given gene. The color intensity represents the gene expression levels. (E) Heatmaps showing the gene expression patterns of CD4^+^ T, CD8^+^ T and NK cell from the scRNA-seq data of tumor tissues of *Trem2*^+/+^ and *Trem2*^-/-^ mice.

**
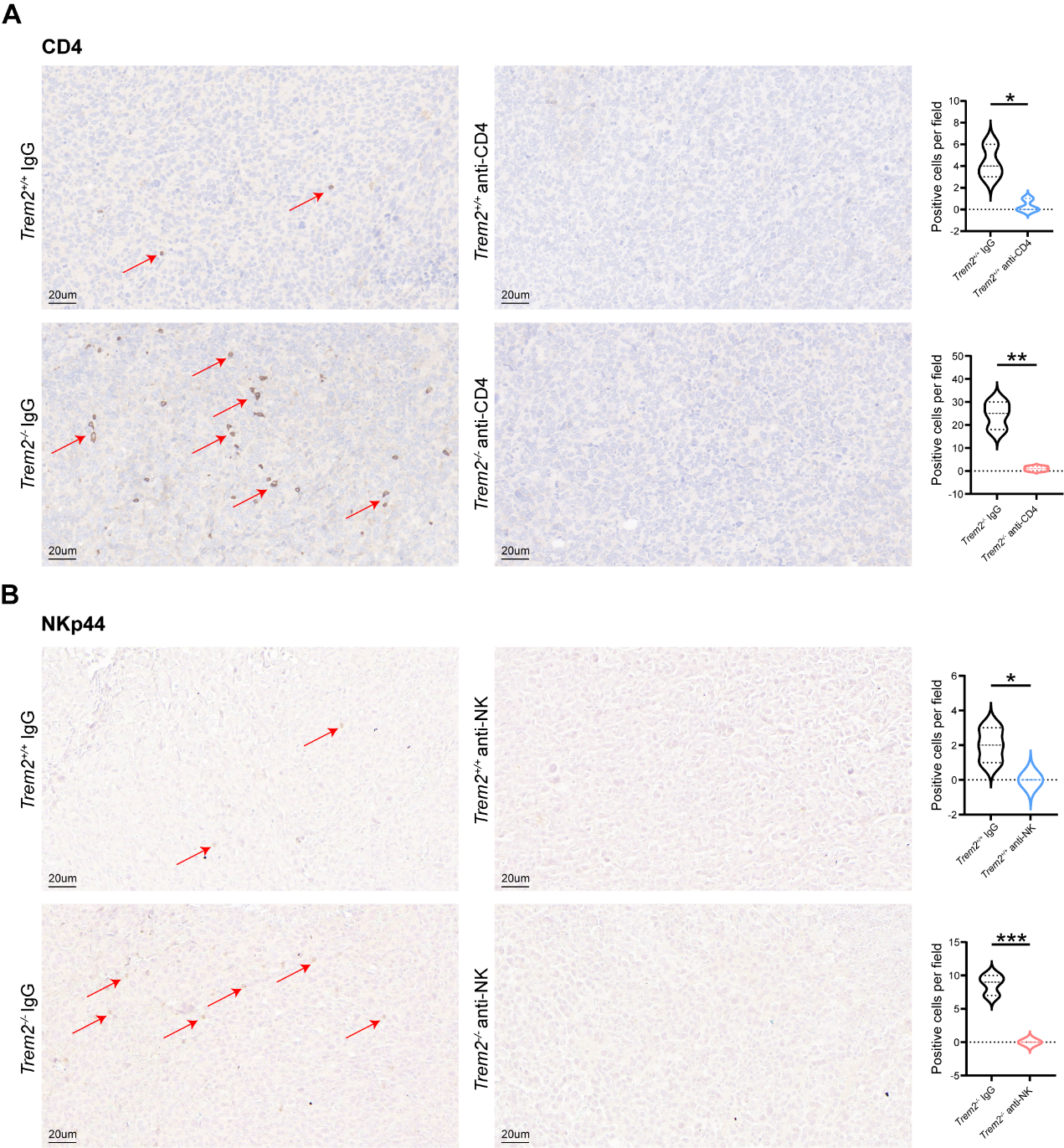
**

**Supplementary Figure 5. The clearance efficiency of CD4^+^ T, CD8^+^ T and NK cells.**

(A) Immunohistochemical staining of CD4 (left) and corresponding analysis (right) in tumor tissues of *Trem2*^+/+^ and *Trem2*^-/-^ mice treated with IgG isotype or anti-CD4 antibody (n = 3 /group). Scale bar, 20 µm. (B) Immunohistochemical staining of NKp44 (left) and corresponding analysis (right) in tumor tissues of *Trem2*^+/+^ and *Trem2*^-/-^ mice treated with IgG isotype, anti-NK antibody (n = 3 /group). Scale bar, 20 µm. Data represent mean ± SEM. Unpaired t test (A, B) was applied. *P < 0.05; **P < 0.01; ***P < 0.001; ****P < 0.0001.

**
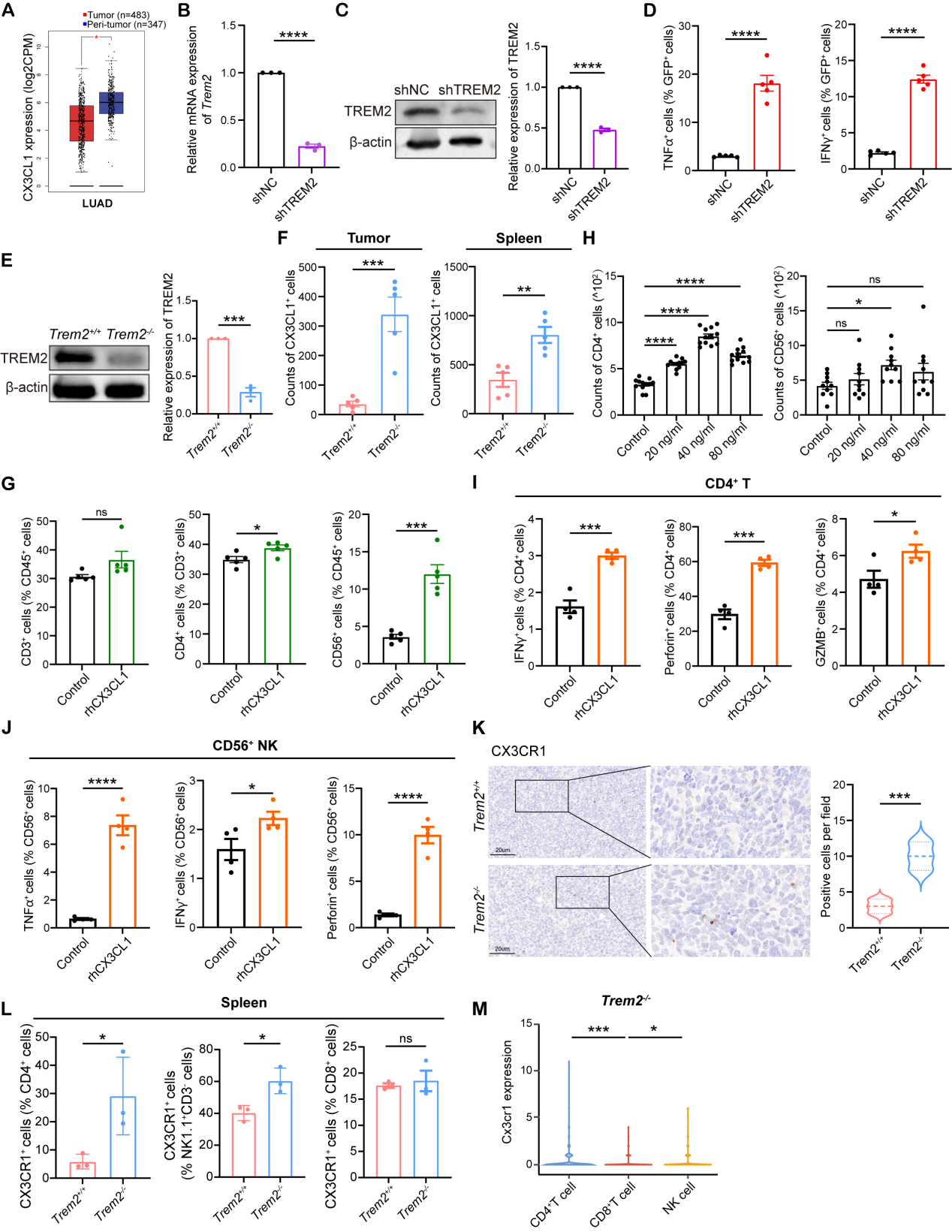
**

**Supplementary Figure 6. CX3CL1 recruits CD4^+^ T and NK cells and enhances their anti-tumor ability.**

(A) The analysis of CX3CL1 expression in tumor and peritumor tissues of LUAD using the GEPIA database. (B) qPCR analysis of *Trem2* expression in shNC or shTREM2 THP-1-derived macrophages (n = 3 /group). (C) Western blot analysis of TREM2 expression (left) and corresponding analysis (right) in shNC or shTREM2 THP-1-derived macrophages (n = 3 /group). (D) Flow cytometry analysis of the percentage of TNFα^+^ and IFNγ^+^ cells in GFP^+^ cells in shNC or shTREM2 THP-1-derived macrophages (n = 5 /group). (E) Western blot analysis of TREM2 expression (left) and corresponding analysis (right) in BMDMs from *Trem2*^+/+^ and *Trem2*^-/-^ mice (n = 3 /group). (F) Flow cytometry analysis of the counts of CX3CL1^+^ cells in tumor tissues (left) and spleens (right) isolated from *Trem2*^+/+^ and *Trem2*^-/-^ mice (n = 5 /group). (G) Transwell assay to examine the recruitment capacity of healthy donor's PBMC-derived CD3^+^ T, CD4^+^ T and CD56^+^ NK cells treated with or without recombinant human CX3CL1 (rhCX3CL1) (40 ng/ml) (n = 5 /group). (H) Transwell assay to examine PBMC-derived CD4^+^ T (n = 12 /group) and CD56^+^ NK (n = 10 /group) cells treated with increasing concentration gradients of rhCX3CL1. (I) Flow cytometry analysis of the percentage of IFNγ^+^, Perforin^+^ and GZMB^+^ cells in CD4^+^ cells from PBMC treated with or without rhCX3CL1 (40 ng/ml) (n = 4 /group). (J) Flow cytometry analysis of the percentage of TNFα^+^, IFNγ^+^, and Perforin^+^ cells in CD56^+^ cells from PBMC treated with or without rhCX3CL1 (40 ng/ml) (n = 4 /group). (K) Immunohistochemical analysis of CX3CR1 in tumor tissues of the *Trem2*^+/+^ and *Trem2*^-/-^ mice (n = 3 /group). Positive staining is indicated by brown coloration. Scale bar, 20 µm. (L) Flow cytometry analysis of the percentage of CX3CR1^+^ cells in CD4^+^ cells, NK1.1^+^CD3^-^ cells and CD8^+^ cells from the spleens of *Trem2*^+/+^ and *Trem2*^-/-^ mice (n = 3 /group). (M) Violin plot illustrating the expression of Cx3cr1 in CD4^+^ T, CD8^+^ T, and NK cells from scRNA-seq of tumor tissues in *Trem2^-/-^* mice (n = 3 /group). Data represent mean ± SEM. Unpaired t test (B, C, D, E, F, G, I, J, K, L) and one-way analysis of variance (ANOVA) (H), and wilcox test (M) were applied. ns：no significance; *P < 0.05; **P < 0.01; ***P < 0.001; ****P < 0.0001.

**
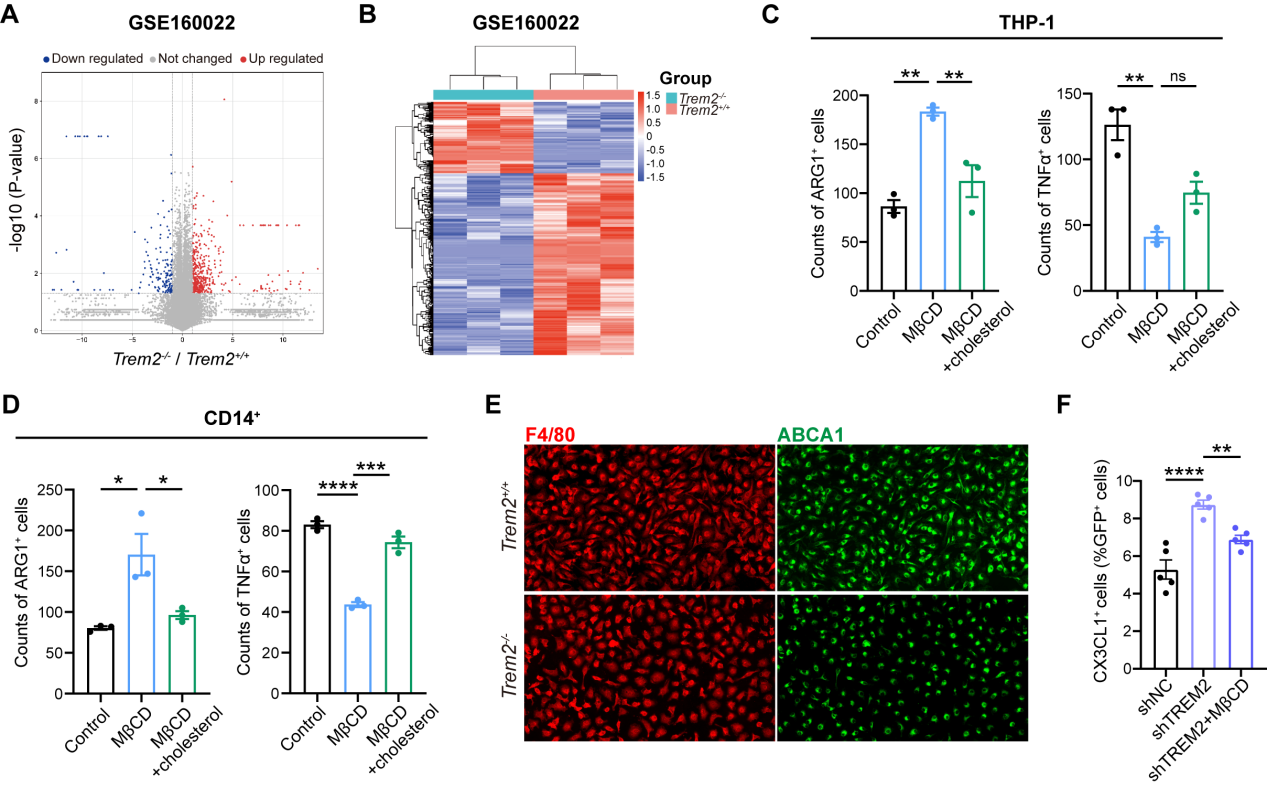
**

**Supplementary Figure 7. Cholesterol accumulation in macrophages promotes their transformation into pro-inflammatory phenotype.**

(A-B) Volcano plot (A) and heatmap (B) showing the DEGs in the transcriptome sequencing of liver macrophages between the *Trem2*^+/+^ and *Trem2*^-/-^ mice from the GSE160022 dataset (criteria: P<0.05 and absolute log2 [fold change] ≧2). (C) Flow cytometry analysis of the counts of ARG1^+^ and TNFα^+^ cells in THP-1-derived macrophages treated with or without (control) cholesterol or MβCD (n = 3 /group). (D) Flow cytometry analysis of the counts of ARG1^+^ and TNFα^+^ cells in CD14^+^ monocytes-derived macrophages treated with or without cholesterol or MβCD (n = 3 /group). (E) Immunofluorescence staining of F4/80 and ABCA1 in BMDM of the *Trem2*^+/+^ and *Trem2*^-/-^ mice. Scale bar, 20 µm. (F) Flow cytometry analysis of the percentage of CX3CL1^+^ cells in GFP^+^ cells respectively in the shNC or shTREM2 THP-1-derived macrophages treated with or without MβCD (n = 5 /group). Data represent mean ± SEM. One-way analysis of variance (ANOVA) (C, D, F) was applied. ns：no significance; *P < 0.05; **P < 0.01; ***P < 0.001; ****P < 0.0001.


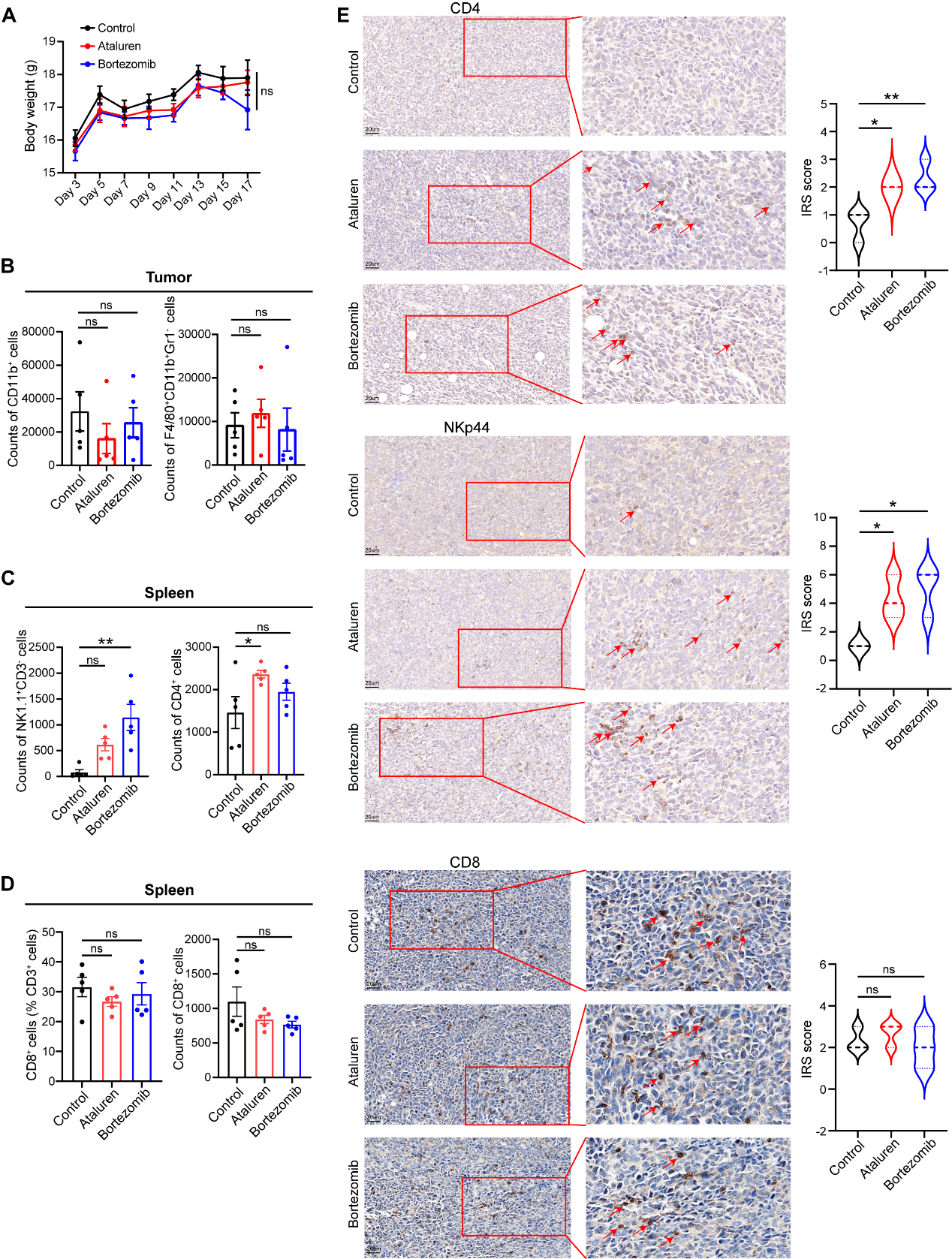


**Supplementary Figure 8. The effect of ataluren and bortezomib on lymphocytes.**

(A) Body weight of C57BL/6N mice treated with or without (control) ataluren or bortezomib were measured until euthanization (n = 5 /group). (B) Flow cytometry analysis of the counts of CD11b^+^ and F4/80^+^CD11b^+^Gr1^-^ cells in tumor tissues collected from C57BL/6N mice treated with or without (control) ataluren or bortezomib (n = 5 /group). (C) Flow cytometry analysis of the counts of NK1.1^+^CD3^-^ and CD4^+^ cells in the spleens of C57BL/6N mice treated with or without (control) ataluren or bortezomib (n = 5 /group). (D) Flow cytometry analysis of the percentage of CD8^+^ cells in CD3^+^ cells and counts of CD8^+^ cells in the spleens of C57BL/6N mice treated with or without (control) ataluren or bortezomib (n = 5 /group). (E) Immunohistochemical staining of CD4, NKp44, and CD8 and corresponding analysis in tumor tissues isolated from C57BL/6N mice treated with or without (control) ataluren or bortezomib (n = 3 /group). Scale bar, 20 µm. Data represent mean ± SEM. Two-way analysis of variance (ANOVA) (A) and one-way ANOVA (B, C, D, E) were applied. ns：no significance; *P < 0.05; **P < 0.01; ***P < 0.001; ****P < 0.0001.
