# Supplementary material for: TREM2‐Mediated Cholesterol Efflux in Macrophages Inhibits Anti‐Tumor Immunity via Limitation of CD4+ T and NK Cells
Source: Adv Sci (Weinh). 2025 Oct 20;13(5):e06995. doi: 10.1002/advs.202506995 (PMC12850164; doi:10.1002/advs.202506995)
Supplement: Supplementary file 2 — Supporting Information [file ADVS-13-e06995-s004.docx]

**Flow Cytometry Antibodies and Cell Lines**

| **REAGENT or RESOURCE** | **SOURCE** | **IDENTIFIER** |
| --- | --- | --- |
| **Antibodies** | | |
| Zombie Aqua™ Fixable Viability Kit | BioLegend | Cat# 423101 |
| Alexa Fluor® 700 anti-mouse/human CD11b Antibody | BioLegend | Cat# 101222 |
| Brilliant Violet 421™ anti-mouse Ly-6G/Ly-6C (Gr-1) Antibody | BioLegend | Cat# 108445 |
| PerCP/Cyanine5.5 anti-mouse F4/80 Antibody | BioLegend | Cat# 123127 |
| PE anti-mouse CD45 Antibody | BioLegend | Cat# 147712 |
| Alexa Fluor® 700 anti-mouse CD3 Antibody | BioLegend | Cat# 100216 |
| PE/Cyanine7 anti-mouse CD3 Antibody | BioLegend | Cat# 100219 |
| PerCP/Cyanine5.5 anti-mouse CD4 Antibody | BioLegend | Cat# 100434 |
| FITC anti-mouse CD8a Antibody | BioLegend | Cat# 100804 |
| APC/Cyanine7 anti-mouse CD49b (pan-NK cells) Antibody | BioLegend | Cat# 108919 |
| Brilliant Violet 421™ anti-mouse NK-1.1 Antibody | BioLegend | Cat# 108731 |
| Brilliant Violet 650™ anti-mouse NK-1.1 Antibody | BioLegend | Cat# 108736 |
| Brilliant Violet 421™ anti-mouse CD86 Antibody | BioLegend | Cat# 105123 |
| PE/Dazzle™ 594 anti-mouse CX3CR1 Antibody | BioLegend | Cat# 149013 |
| Mouse CX3CL1/Fractalkine PE-conjugated Antibody | R&D | Cat# FAB571P-025 |
| Human/Mouse TREM2 Alexa Fluor® 488‑conjugated Antibody | R&D | Cat# FAB17291G |
| APC anti-mouse Perforin Antibody | BioLegend | Cat# 154304 |
| Brilliant Violet 421™ anti-human/mouse Granzyme B Recombinant Antibody | BioLegend | Cat# 396414 |
| PE anti-mouse IFN-γ Antibody | BioLegend | Cat# 505808 |
| Brilliant Violet 605™ anti-mouse TNF-α Antibody | BioLegend | Cat# 506329 |
| [FITC anti-human CD45 Antibody](https://www.biolegend.com/en-gb/products/fitc-anti-human-cd45-antibody-707) | BioLegend | Cat# 304005 |
| [PerCP/Cyanine5.5 anti-human CD68 Antibody](https://www.biolegend.com/en-gb/products/percp-cyanine5-5-anti-human-cd68-antibody-7007) | BioLegend | Cat# 333813 |
| APC/Cyanine7 anti-human CD206 (MMR) Antibody | BioLegend | Cat# 321119 |
| PE/Cyanine7 anti-human CD56 (NCAM) Antibody | BioLegend | Cat# 318318 |
| Brilliant Violet 605™ anti-human CD3 Antibody | BioLegend | Cat# 317321 |
| PerCP/Cyanine5.5 anti-human CD4 Antibody | BioLegend | Cat# 300529 |
| PE/Cyanine7 anti-human CD4 Antibody | BioLegend | Cat# 300511 |
| Human/Mouse TREM2 PE-conjugated Antibody | R&D | Cat# FAB17291P |
| PE/Cyanine7 anti-human Arginase I Antibody | BioLegend | Cat# 369707 |
| Arginase 1 Monoclonal Antibody (A1exF5), Alexa Fluor™ 488, eBioscience™ | ThermoFisher | Cat# 53-3697-80 |
| Brilliant Violet 605™ anti-human TNF-α Antibody | BioLegend | Cat# 502935 |
| PerCP/Cyanine5.5 anti-human/mouse Granzyme B Recombinant Antibody | BioLegend | Cat# 372211 |
| Brilliant Violet 421™ anti-human Perforin Antibody | BioLegend | Cat# 308121 |
| [PE/Cyanine7 anti-human Perforin Antibody](https://www.biolegend.com/en-gb/products/pe-cyanine7-anti-human-perforin-antibody-12957) | BioLegend | Cat# 353315 |
| APC anti-human IFN-γ Antibody | BioLegend | Cat# 502512 |
| FITC anti-human IFN-γ Antibody | BioLegend | Cat# 502505 |
| Human CX3CL1/Fractalkine Chemokine Domain Alexa Fluor® 700-conjugated Antibody | R&D | Cat# IC3652N |
| Human CX3CL1/Fractalkine Alexa Fluor® 750-conjugated Antibody | R&D | Cat# IC3652RS |
| **Cell lines** | | |
| LLC cell line (Mouse) | Pricella | Cat# CL-0140 |
| A549 cell line (Human) | the Cell Bank and the Stem Cell Bank of the Chinese Academy of Sciences | Cat# TCHu150 |
| H322 cell line (Human) | Cellosaurus | Cat# CVCL_1556 |
| 293T cell line (Human) | the Cell Bank and the Stem Cell Bank of the Chinese Academy of Sciences | Cat# GNHu17 |
| THP-1 cell line (Human) | the Cell Bank and the Stem Cell Bank of the Chinese Academy of Sciences | Cat# SCSP-567 |
