# Supplementary material for: TREM2‐Mediated Cholesterol Efflux in Macrophages Inhibits Anti‐Tumor Immunity via Limitation of CD4+ T and NK Cells
Source: Adv Sci (Weinh). 2025 Oct 20;13(5):e06995. doi: 10.1002/advs.202506995 (PMC12850164; doi:10.1002/advs.202506995)

The flow cytometry plots and gating strategies

Fig 1G THP-1 control vs. A549 CM

Gating Strategy for the percentages of CD206, TREM2 and ARG1 expressions on THP-1 derived macrophages incubated with or without A549 CM.

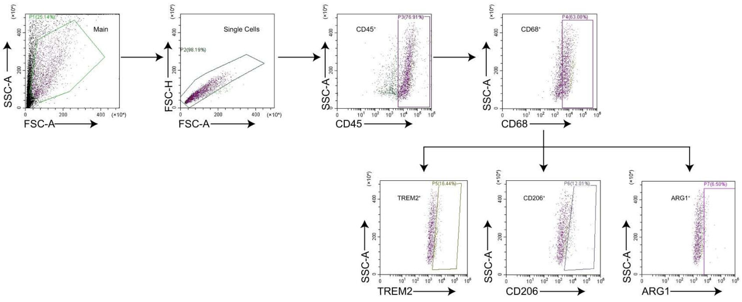

Fig 1G THP-1 control vs. H322 CM

Gating Strategy for the percentages of CD206, TREM2 and ARG1 expressions on THP-1 derived macrophages incubated with or without H322 CM.

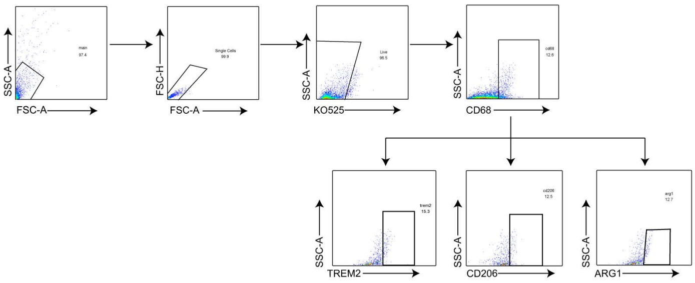

Fig 1G CD14 control vs. A549 CM / H322 CM

Gating Strategy for the percentages of CD206, TREM2 and ARG1 expressions on CD14+ monocytes derived macrophages incubated with or without A549 CM or H322 CM.

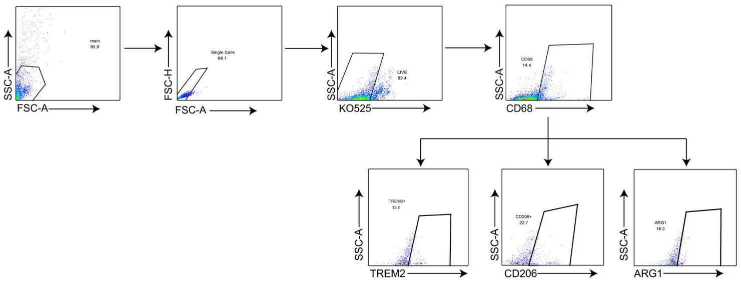

Fig 2H-I Tumor / Spleen *Trem2*$^{+/+}$ vs. *Trem2*$^{-/-}$

Gating Strategy for the percentages of Perforin, TNFα, and GZMB of CD4^{+} T and NK cells in both tumor tissues and spleens from *Trem2*$^{+/+}$ and *Trem2*$^{-/-}$ mice.

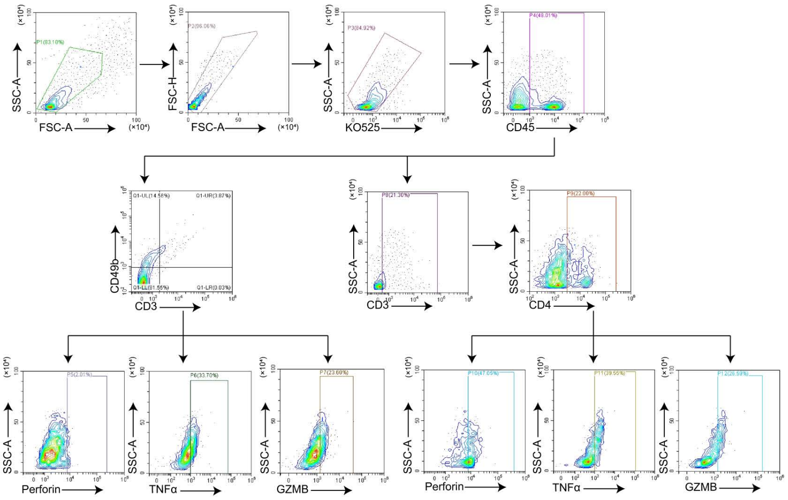

Fig 3B,J Spleen *Trem2*$^{+/+}$ vs. *Trem2*$^{-/-}$

Gating Strategy for the percentages of CD4^{+} T, CD8^{+} T, or NK cells in the spleens of *Trem2*$^{+/+}$ and *Trem2*$^{-/-}$ mice treated with IgG isotype, anti-CD4, anti-CD8 or anti-NK antibodies.

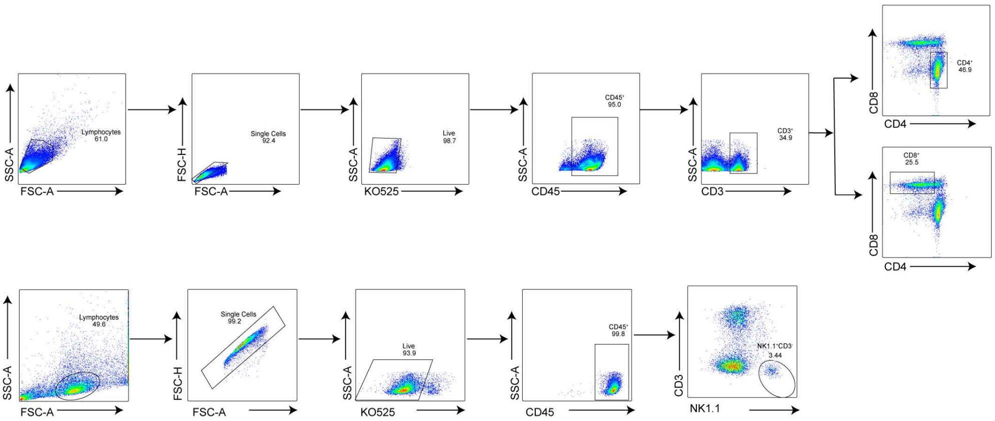

Fig 4F THP-1 shNC vs. shTREM2

Gating Strategy for the percentages of CX3CL1^{+} cells in shNC or shTREM2 THP-1-derived macrophages.

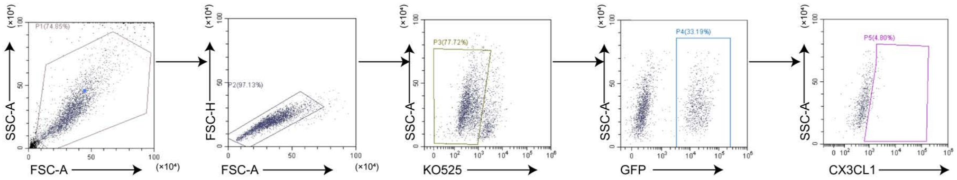

Fig 4H BMDM *Trem2*$^{+/+}$ vs. *Trem2*$^{-/-}$

Gating Strategy for the percentage of CD86^{+} cells and CD206^{+} cells in BMDMs of *Trem2*$^{+/+}$ and *Trem2*$^{-/-}$ mice.

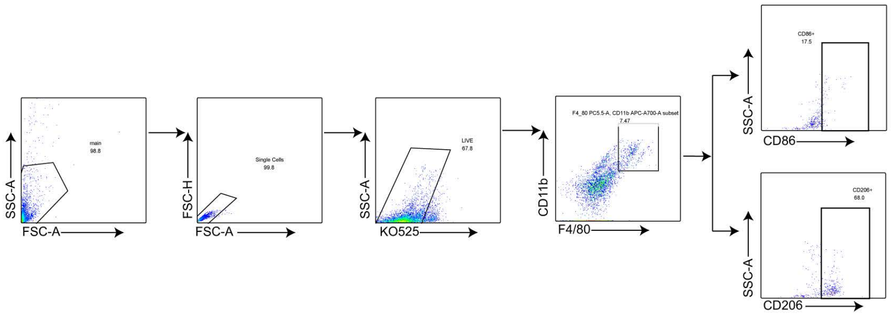

Fig 4J BMDM *Trem2*$^{+/+}$ vs. *Trem2*$^{-/-}$

Gating Strategy for the percentage of CX3CL1^{+} cells from BMDMs of *Trem2*$^{+/+}$ and *Trem2*$^{-/-}$ mice.

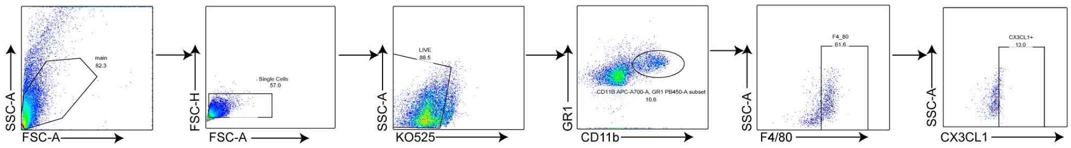

Fig 4K Tumor *Trem2*$^{+/+}$ vs. *Trem2*$^{-/-}$

Gating Strategy for the percentage of CX3CL1^{+} cells from tumor tissues of *Trem2*$^{+/+}$ and *Trem2*$^{-/-}$ mice.

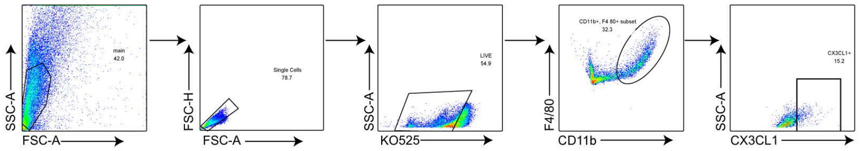

Fig 4L Spleen *Trem2*$^{+/+}$ vs. *Trem2*$^{-/-}$

Gating Strategy for the percentage of CX3CL1^{+} cells from spleens of *Trem2*$^{+/+}$ and *Trem2*$^{-/-}$ mice.

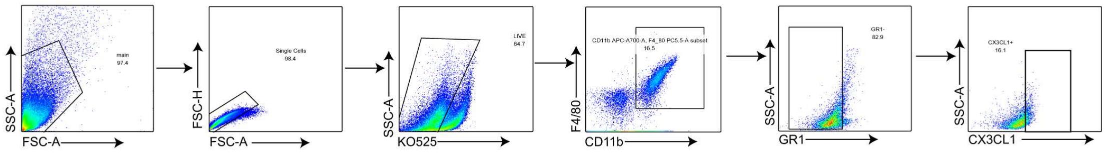

Fig 4O Tumor *Trem2*$^{+/+}$ vs. *Trem2*$^{-/-}$

Gating Strategy for the percentage of CX3CR1^{+} cells in CD4^{+} cells, NK1.1^{+}CD3^{-} cells and CD8^{+} cells from the tumor tissues of *Trem2*$^{+/+}$ and *Trem2*$^{-/-}$ mice.

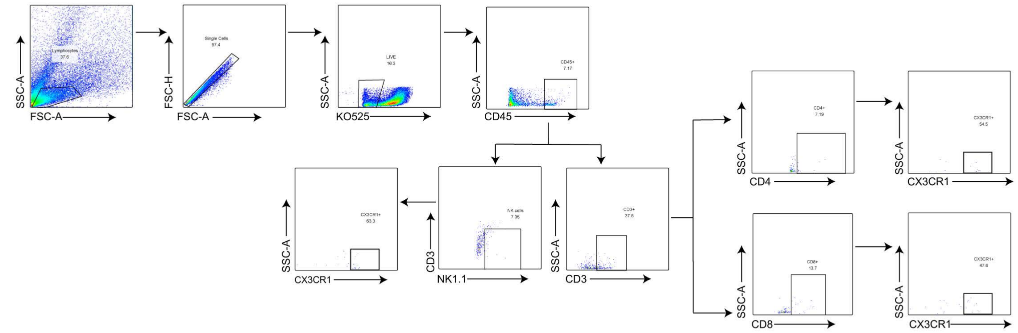

Fig 5I THP-1 / CD14 control vs. MβCD vs. MβCD+cholesterol

Gating Strategy for the percentage of ARG1^{+} and TNFα^{+} cells in CD68^{+} cells on THP-1 or CD14^{+} monocytes derived macrophages treated with or without cholesterol or MβCD.

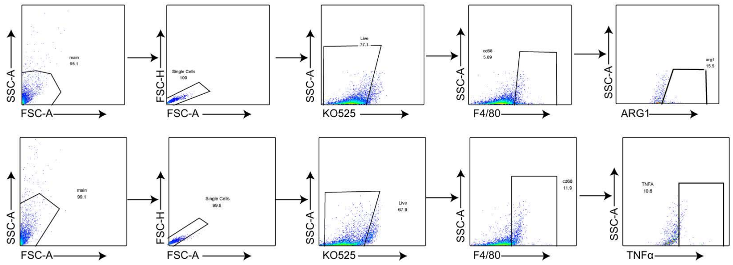

Fig 6H THP-1 control vs. MβCD vs. cholesterol vs. cholesterol+MβCD

Gating Strategy for the percentage of CX3CL1^{+} in CD68^{+} cells on THP-1 derived macrophages treated with or without cholesterol or MβCD.

Fig 6K THP-1 increasing concentration of cholesterol (0, 2, 5, 10 ug/mL)

Gating Strategy for the percentage of CX3CL1^{+} in CD68^{+} cells on THP-1 derived macrophages treated with increasing concentration of cholesterol.

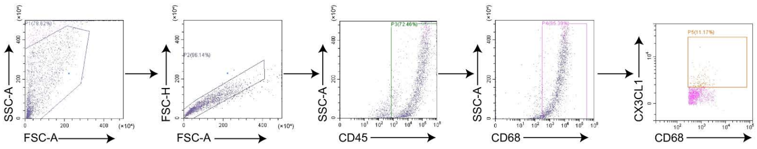

Fig 6H CD14 control vs. MβCD vs. cholesterol vs. cholesterol+MβCD

Gating Strategy for the percentage of CX3CL1^{+} in CD68^{+} cells on CD14^{+} monocytes derived macrophages treated with or without cholesterol or MβCD.

Fig 6K CD14 increasing concentration of cholesterol (0, 2, 5, 10 ug/mL)

Gating Strategy for the percentage of CX3CL1^{+} in CD68^{+} cells on CD14^{+} monocytes derived macrophages treated with increasing concentration of cholesterol.

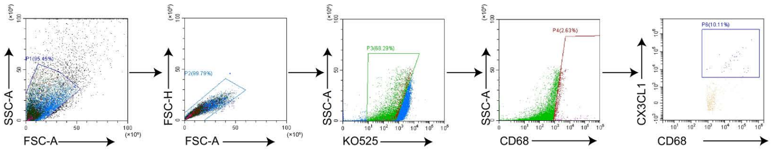

Fig 7K / Fig S8B Tumor control vs. ataluren vs. bortezomib

Gating Strategy for the percentages of TREM2$^+$ cells of macrophages in the tumor tissues of C57/BL6N mice treated with or without ataluren or bortezomib.

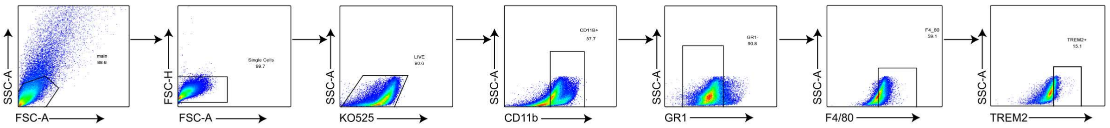

Fig 7K / Fig S8C,D Spleen control vs. ataluren vs. bortezomib

Gating Strategy for the percentages of CD4$^+$ T, CD8$^+$ T, or NK cells in the spleens of C57/BL6N mice treated with or without ataluren or bortezomib.

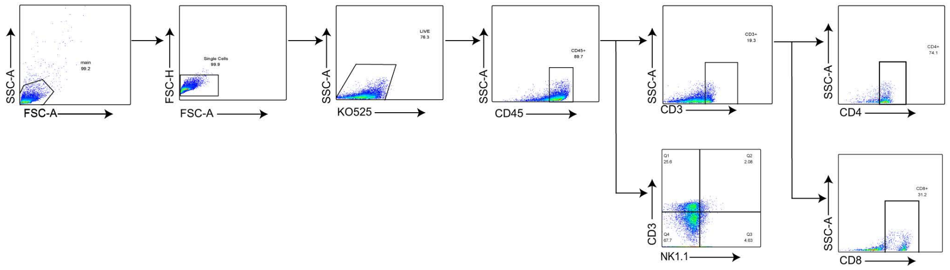

Fig S6D THP-1 shNC vs. shTREM2

Gating Strategy for the percentages of TNFα⁺ and IFNγ⁺ cells in shNC or shTREM2 THP-1-derived macrophages.

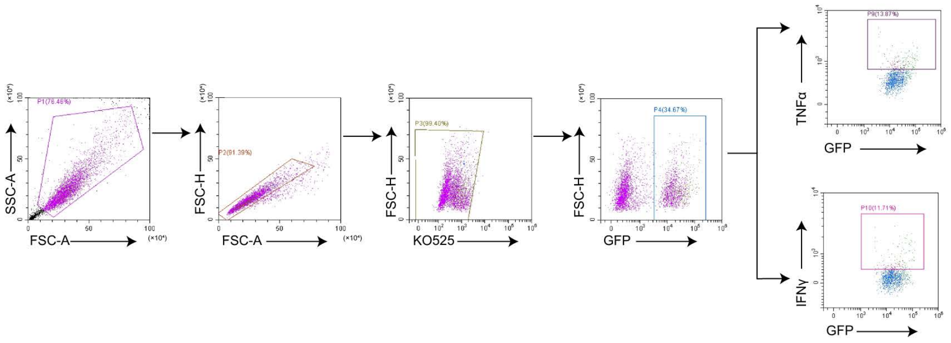

Fig S6G PBMC control vs. rhCX3CL1

Gating Strategy for the percentages of CD3⁺, CD4⁺ T, and CD56⁺ cells in PBMC treated with or without rhCX3CL1.

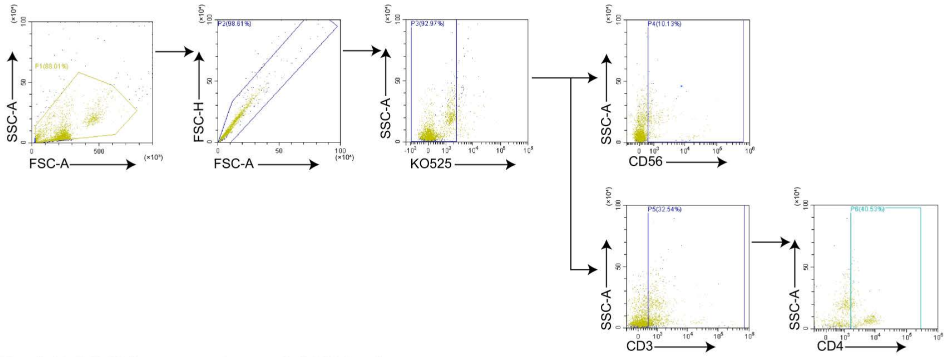

Fig S6I PBMC control vs. rhCX3CL1

Gating Strategy for the percentages of IFNγ⁺, Perforin⁺, and GZMB⁺ cells in CD4⁺ T cells from PBMC treated with or without rhCX3CL1.

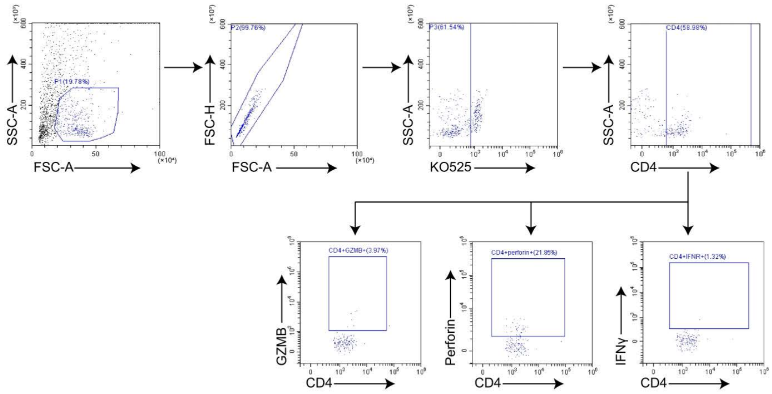

Fig S6J PBMC control vs. rhCX3CL1

Gating Strategy for the percentages of IFNγ⁺, Perforin⁺, and GZMB⁺ cells in CD56⁺ cells from PBMC treated with or without rhCX3CL1.

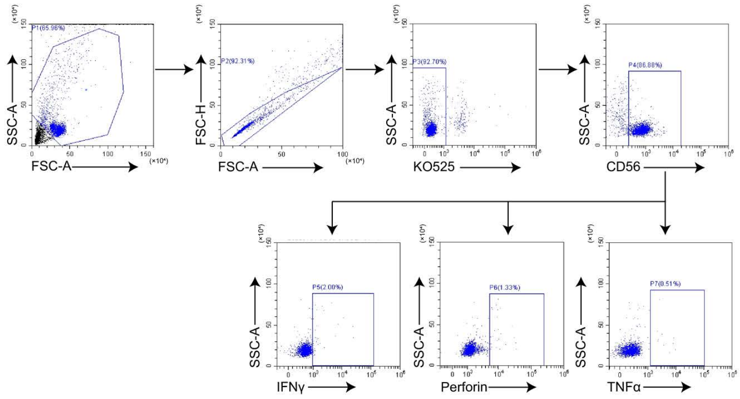

Fig S6L Spleen *Trem2*$^{+/+}$ vs. *Trem2*$^{-/-}$

Gating Strategy for the percentage of CX3CR1^{+} cells in CD4^{+} cells, NK1.1^{+}CD3^{-} cells and CD8^{+} cells from the spleens of *Trem2*$^{+/+}$ and *Trem2*$^{-/-}$ mice.

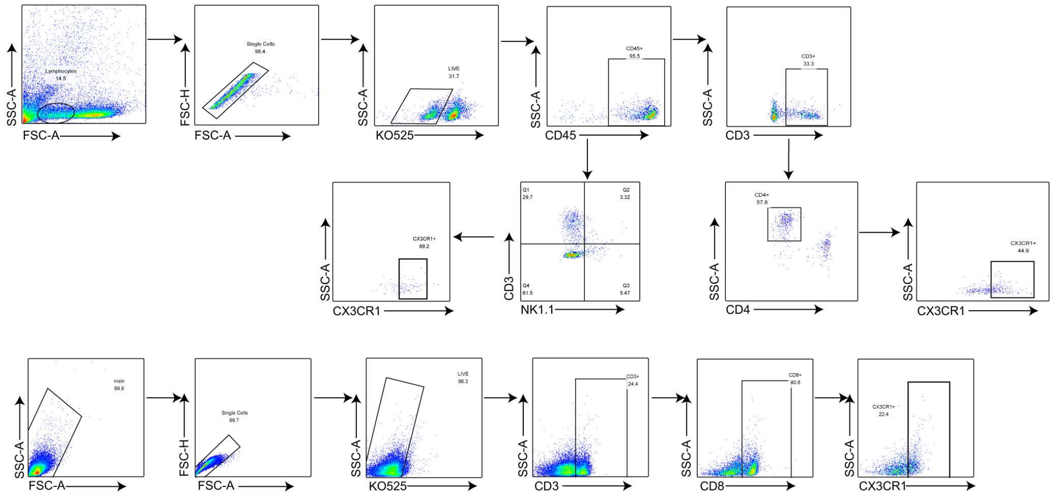

Fig S7F THP-1 shNC vs. shTREM2

Gating Strategy for the percentages of CX3CL1^{+} cells in shNC or shTREM2 THP-1-derived macrophages.

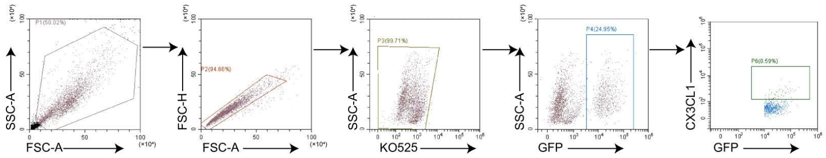

Supplement: Supplementary file 3 — Supporting Information [file ADVS-13-e06995-s003.pdf]
