# Supplementary material for: TREM2‐Mediated Cholesterol Efflux in Macrophages Inhibits Anti‐Tumor Immunity via Limitation of CD4+ T and NK Cells
Source: Adv Sci (Weinh). 2025 Oct 20;13(5):e06995. doi: 10.1002/advs.202506995 (PMC12850164; doi:10.1002/advs.202506995)

**Original Western Blot Figures**

**Figure 1H:**

**①②**


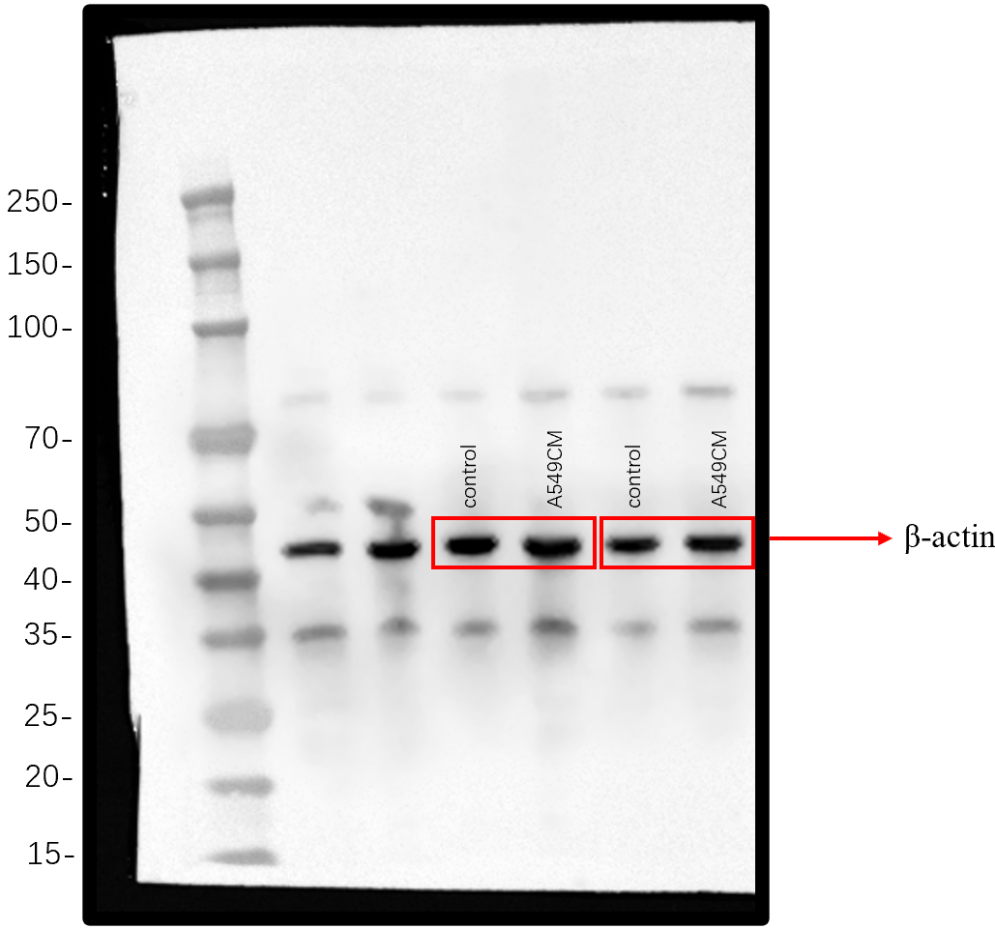


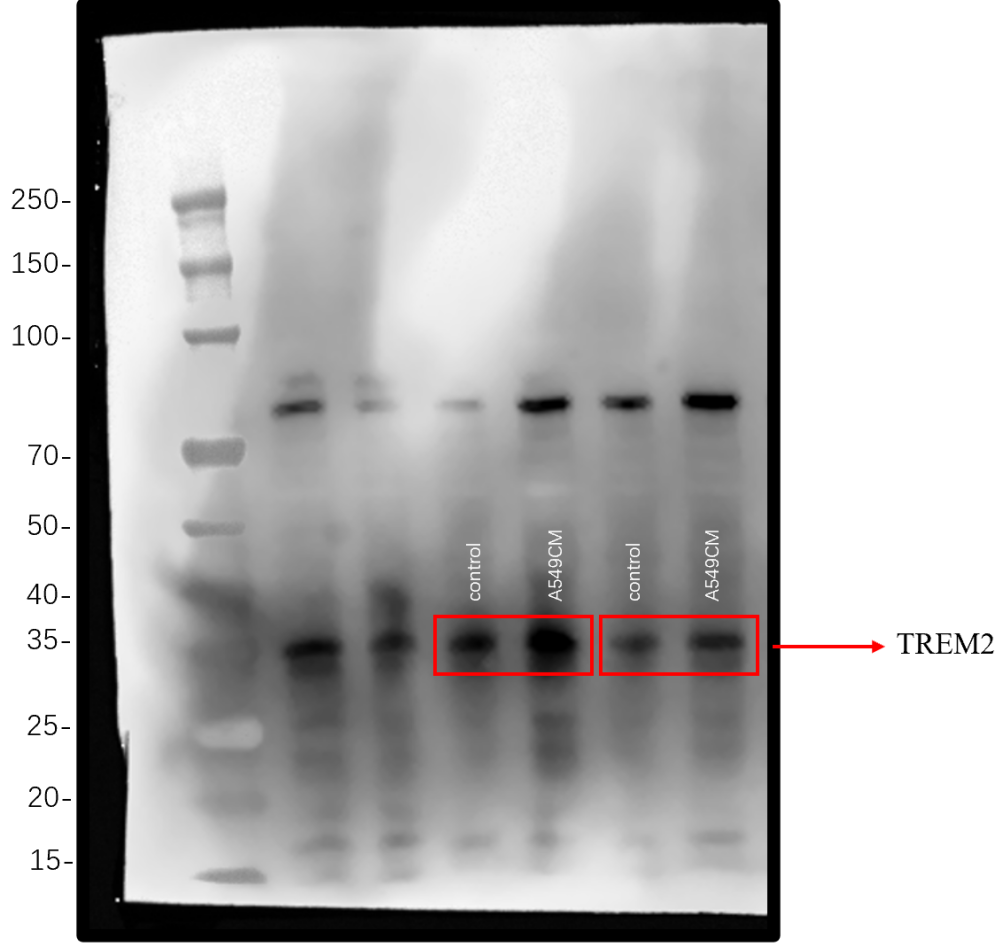


**③**

**
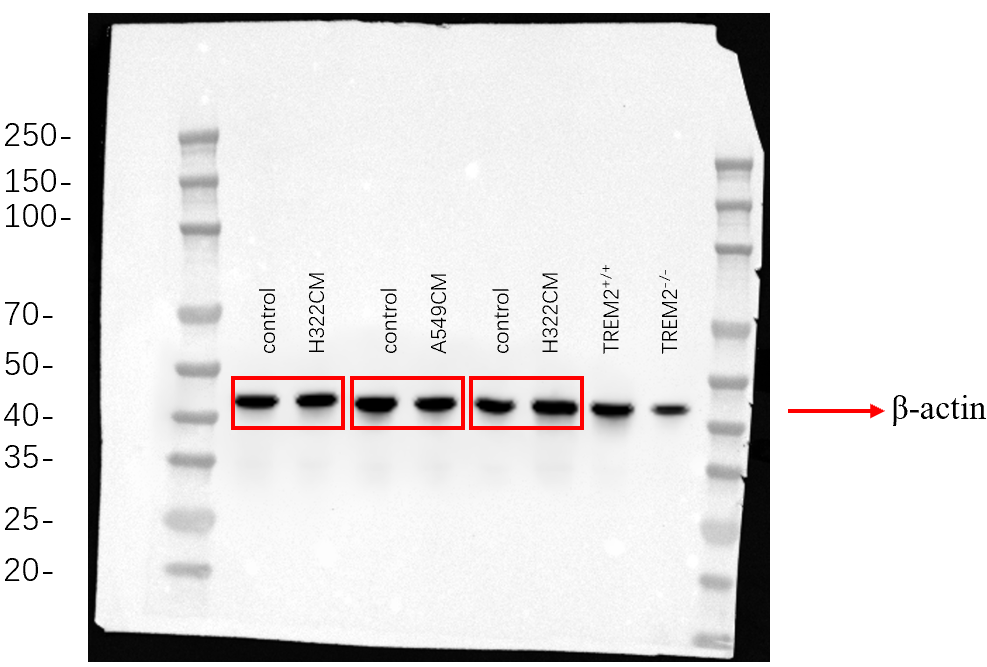
**

**
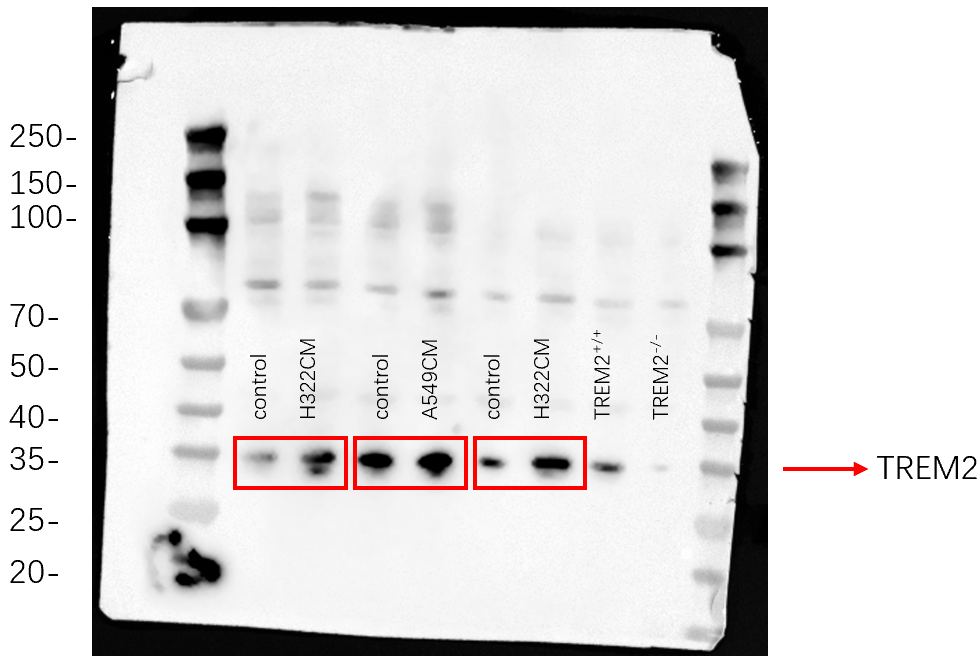
**

**Figure 1H:**

**①②**

**
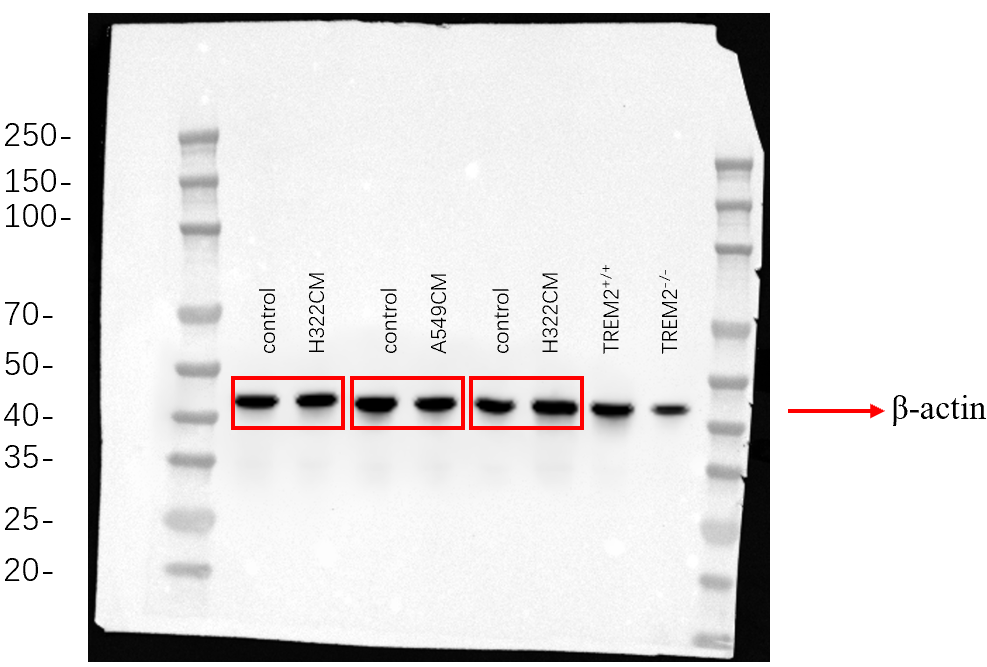
**

**
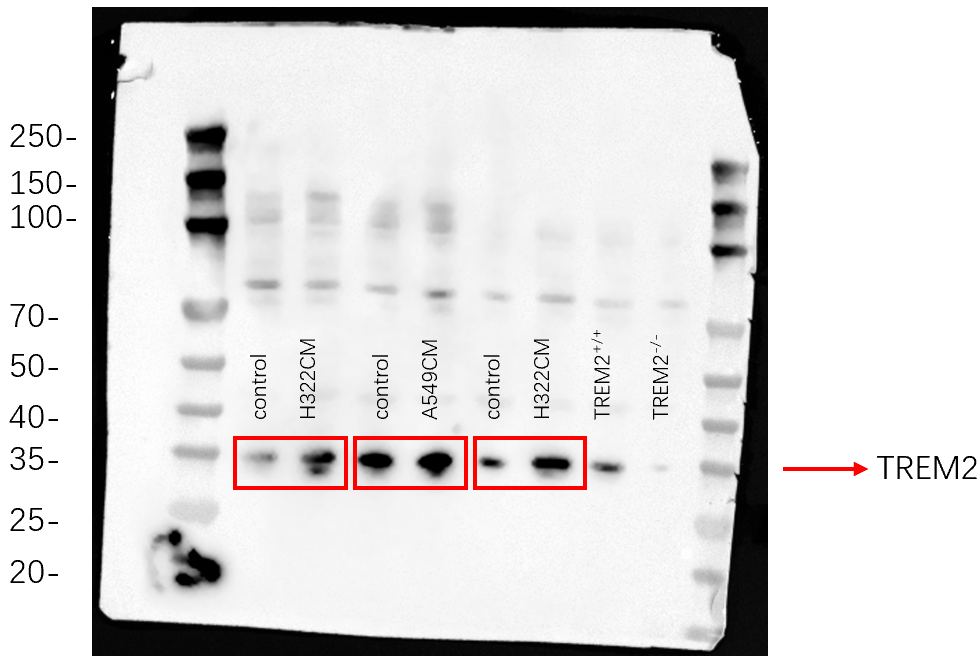
**

**③**

**
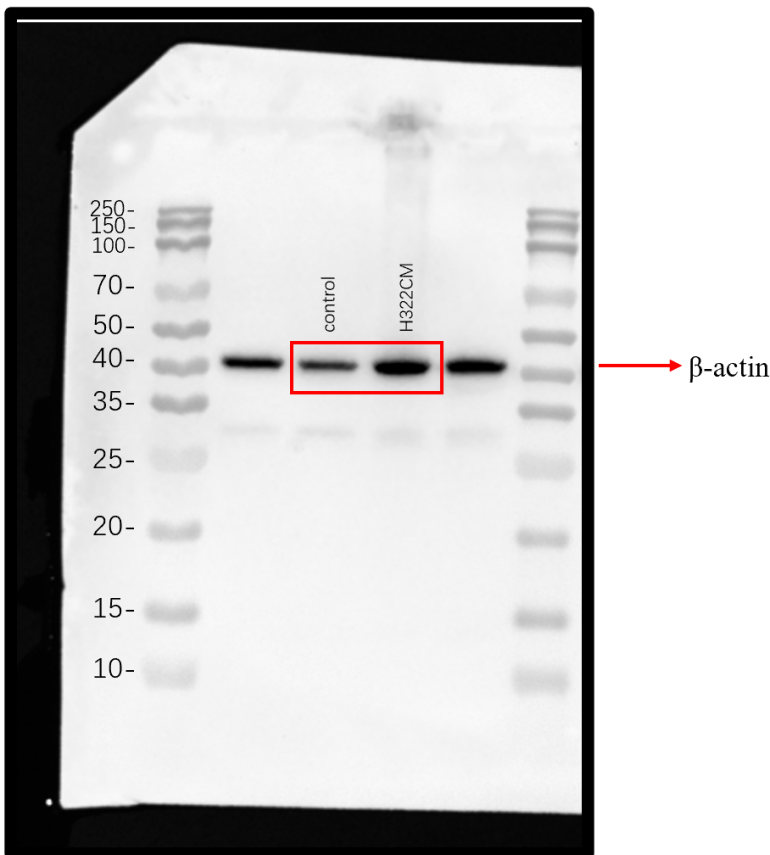
**

**
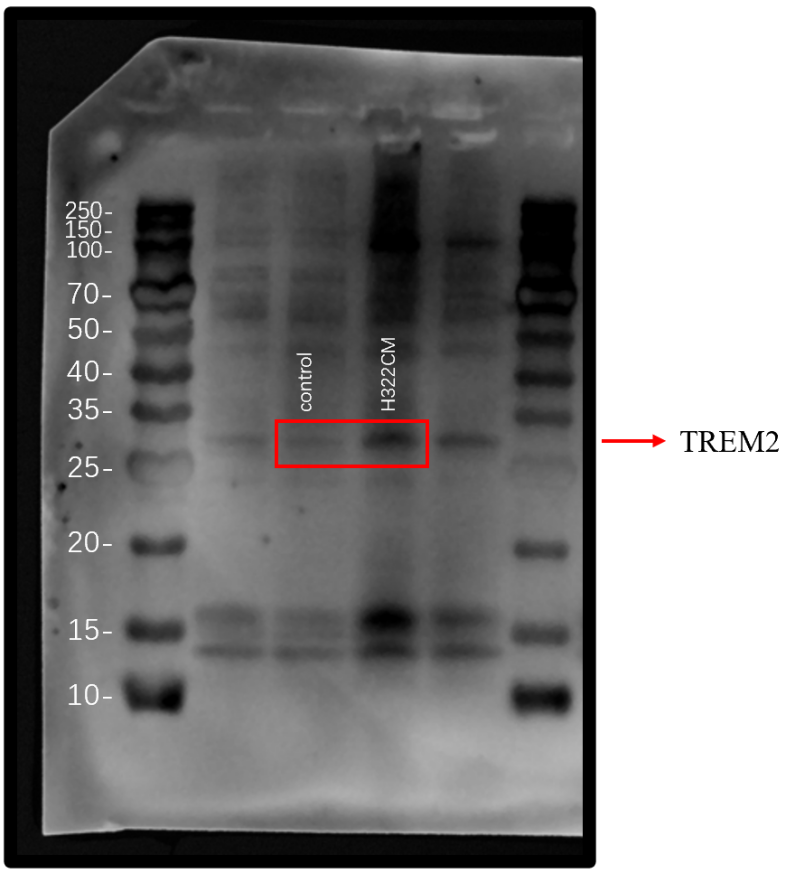
**

**Figure 4E:**

**①**

**
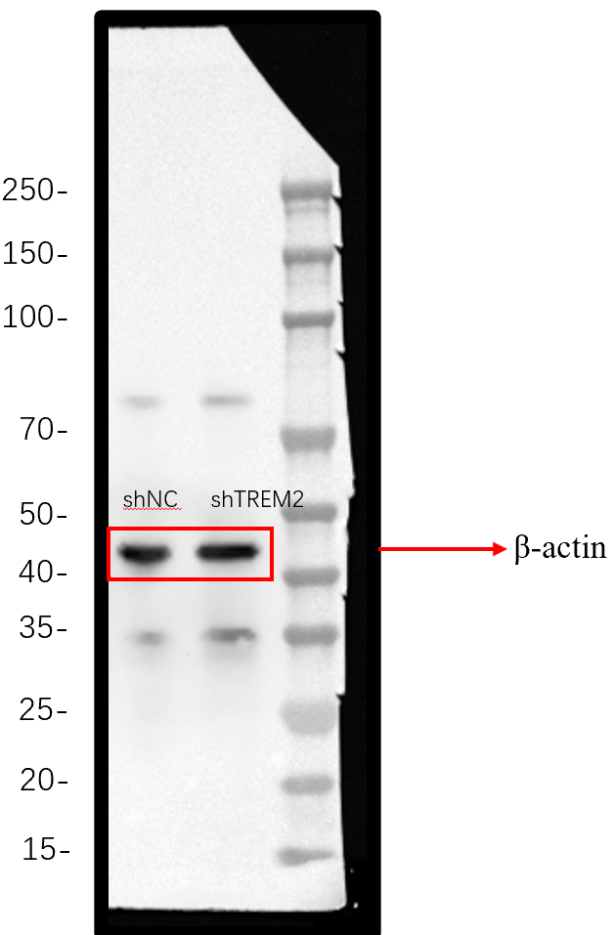
**

**
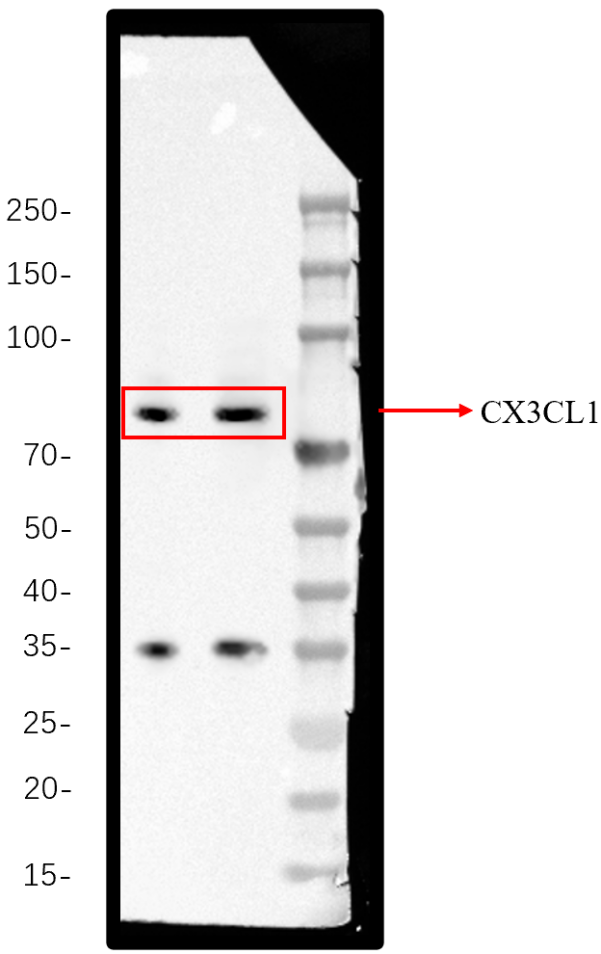
**

**②③**


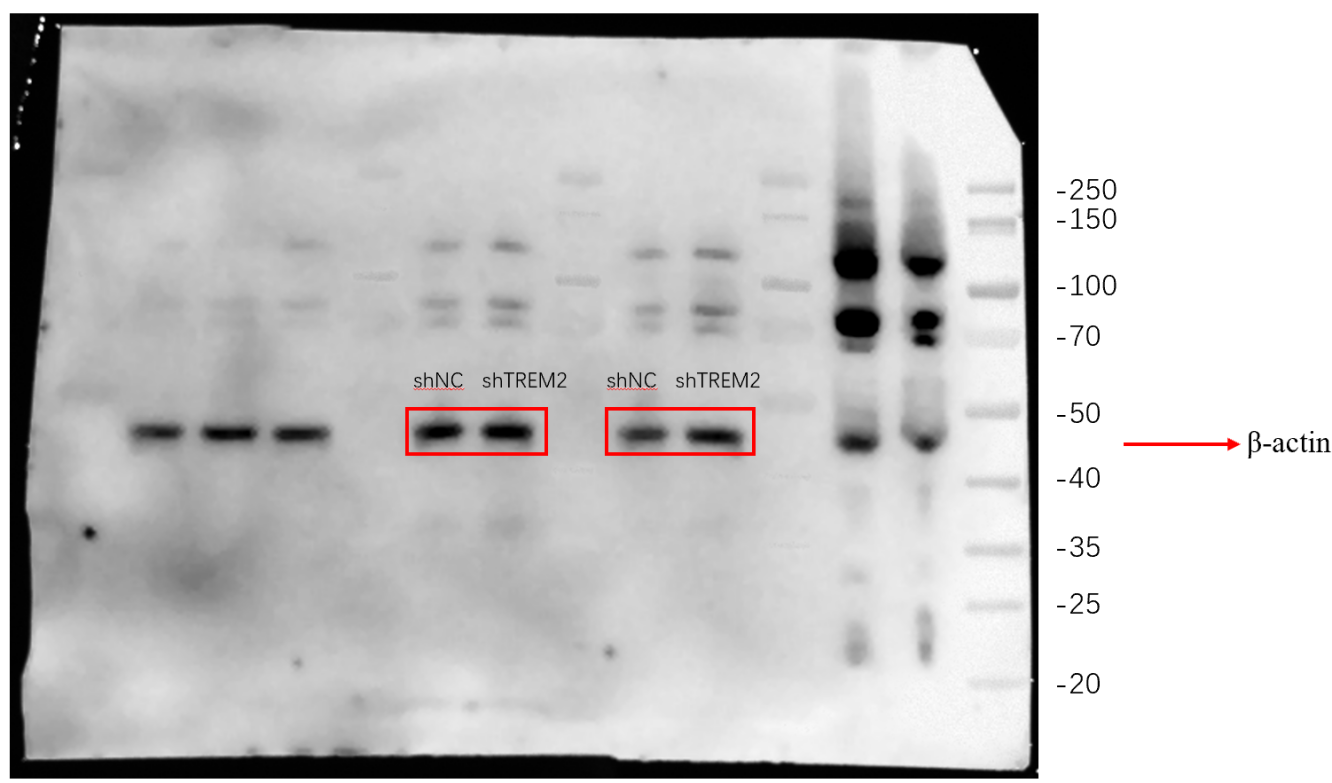

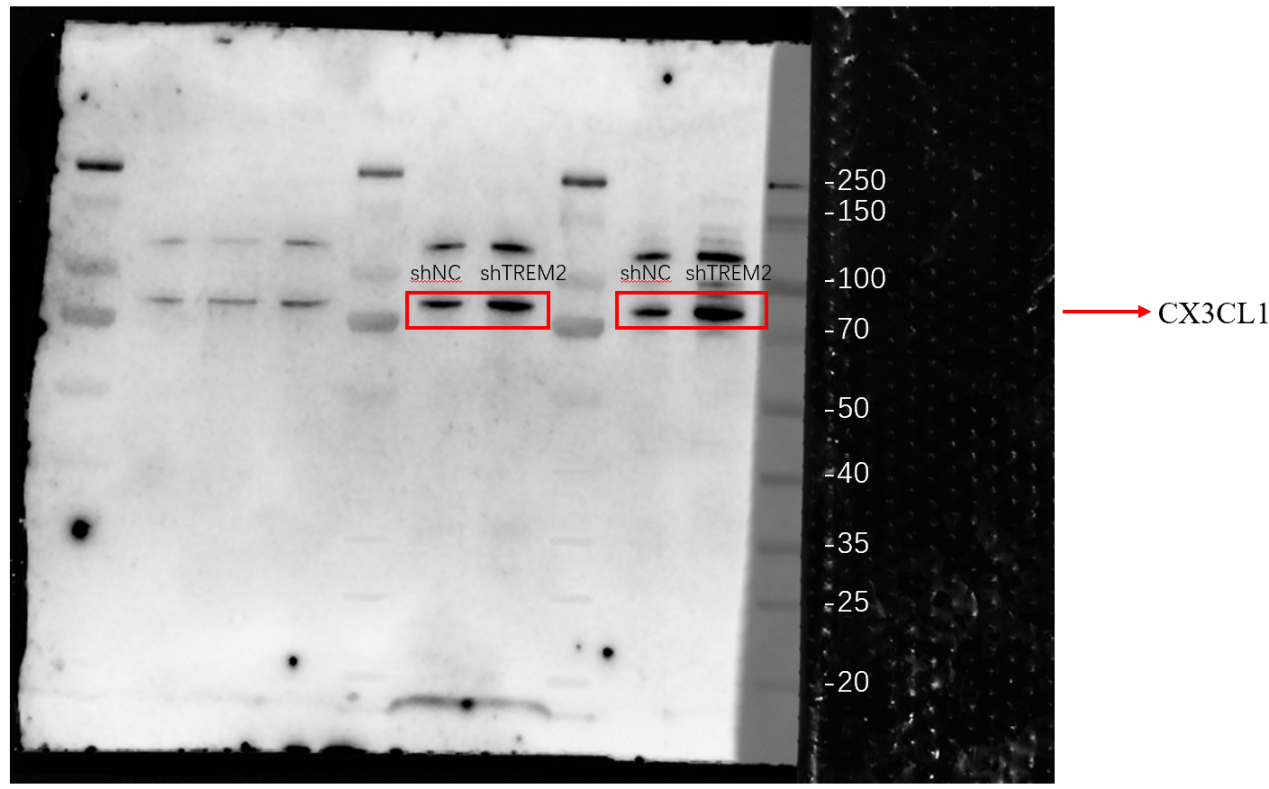


**Figure 4I:**

**①②③**


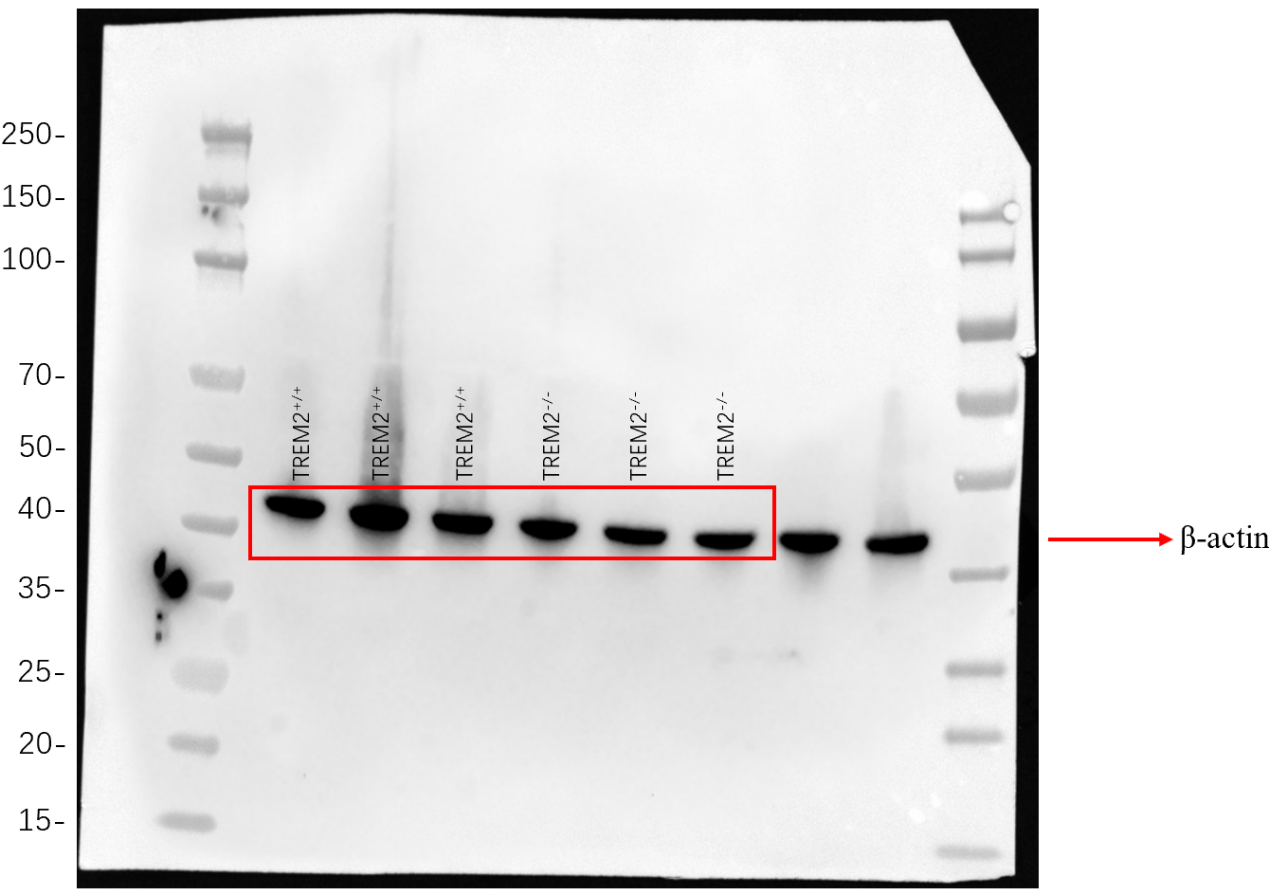


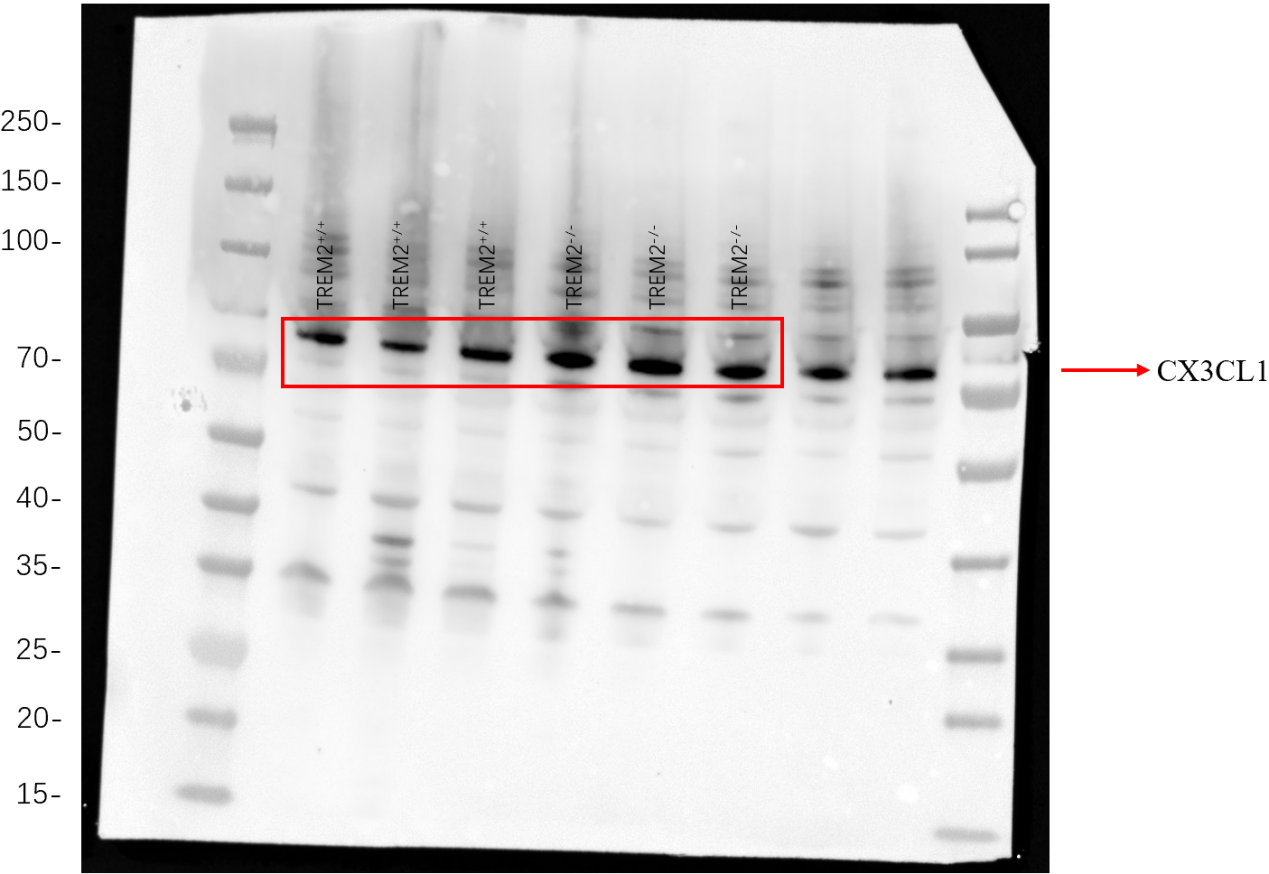


**Figure 6D:**

**①**


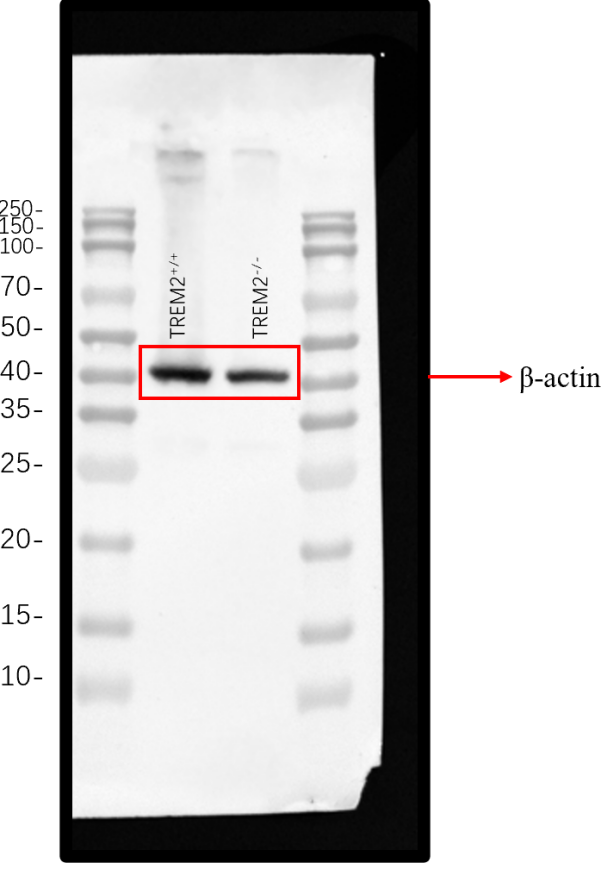


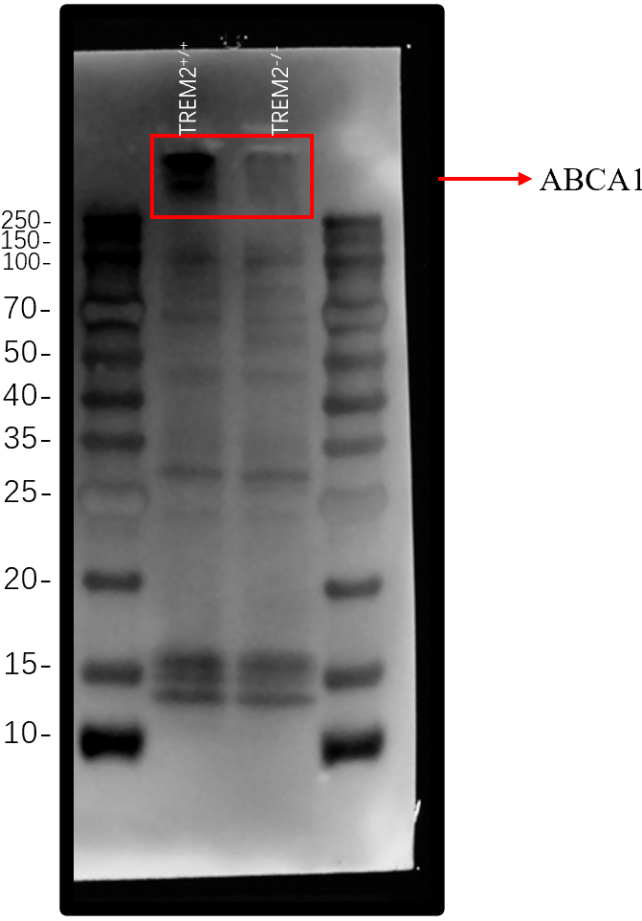


**②**


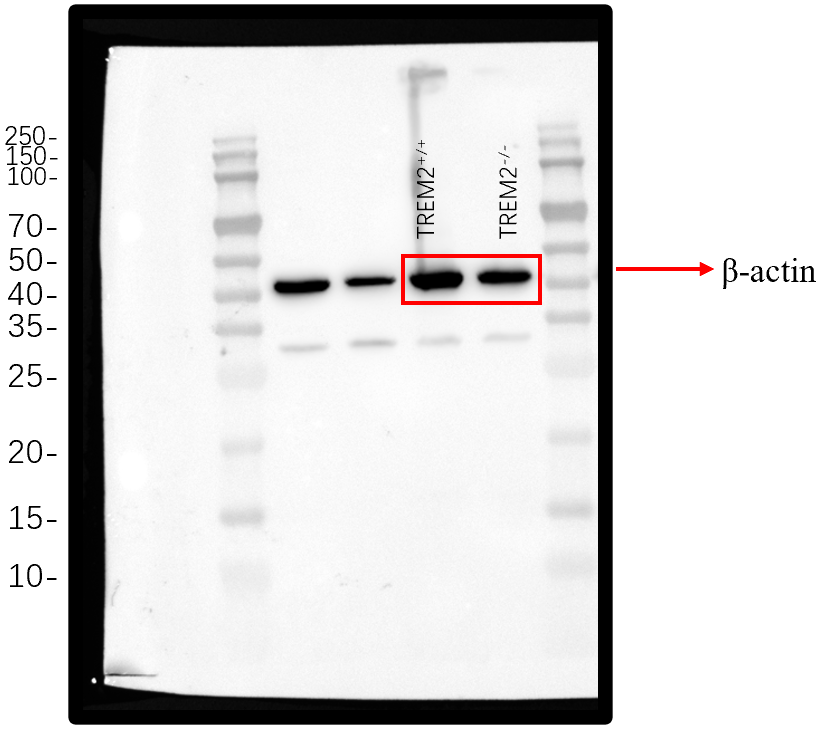


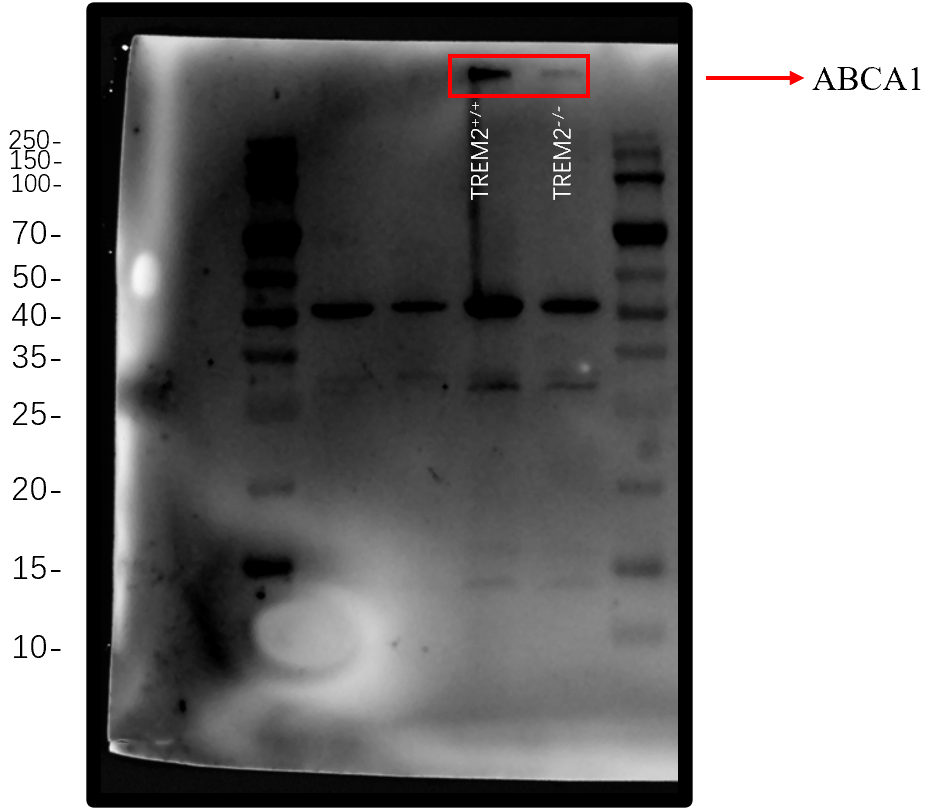


**③**


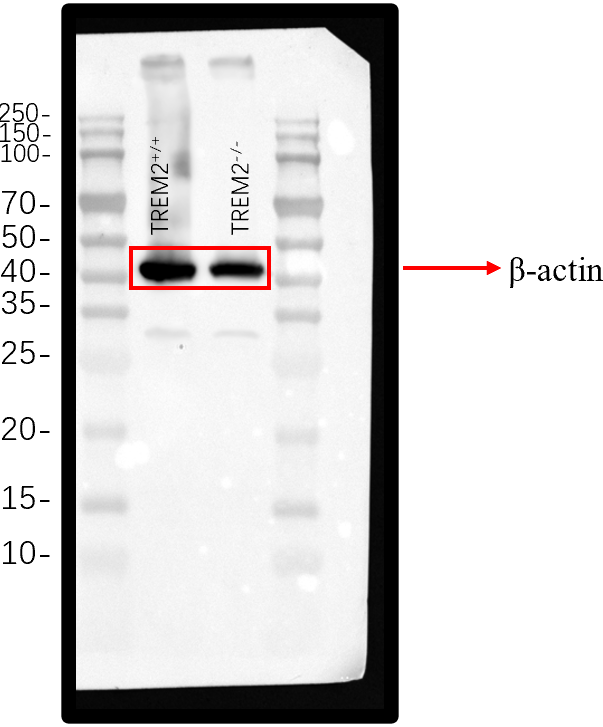


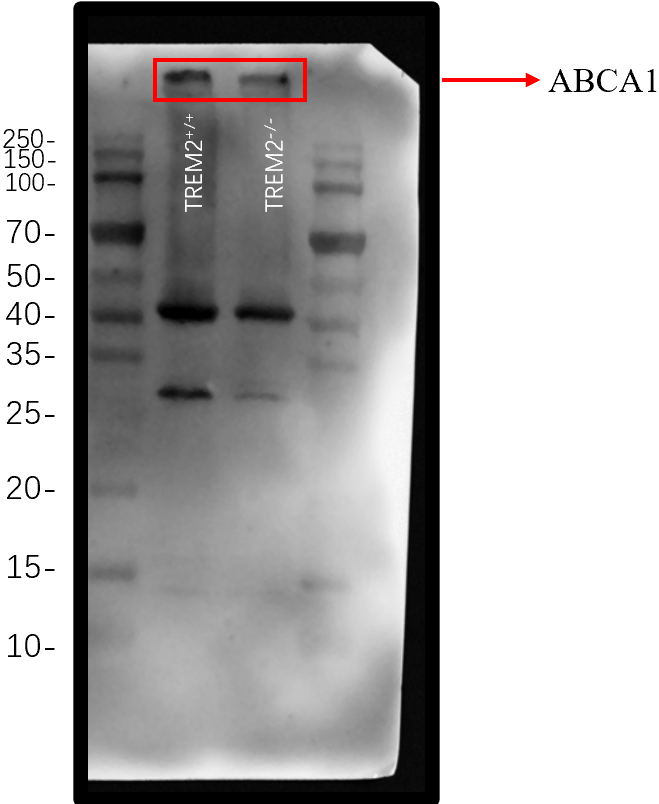


**Figure 6E:**

**①**


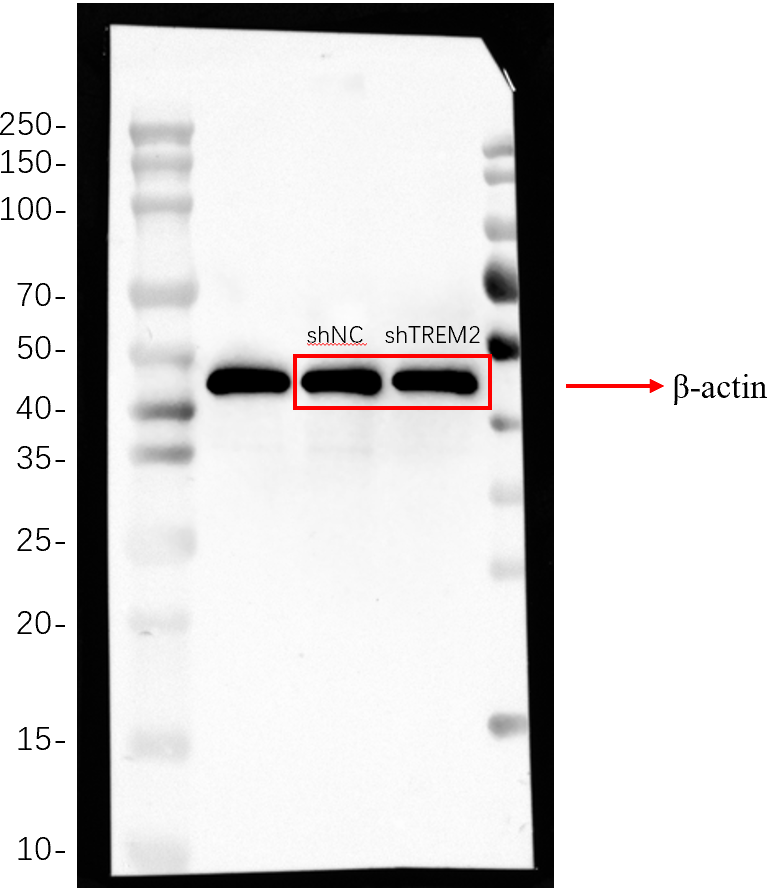


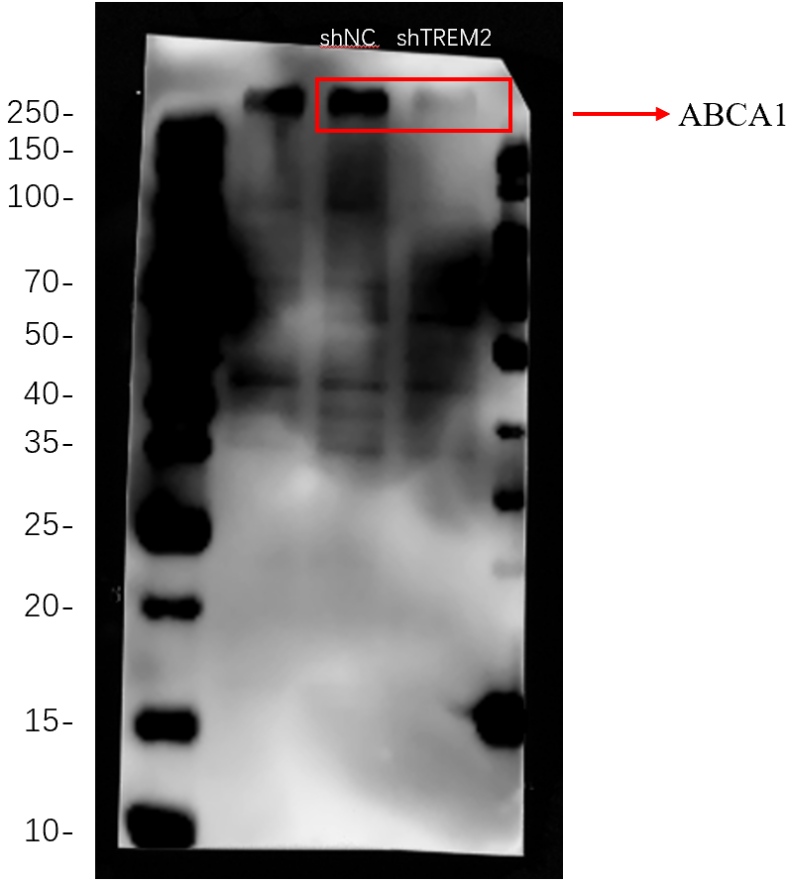


**②**

**
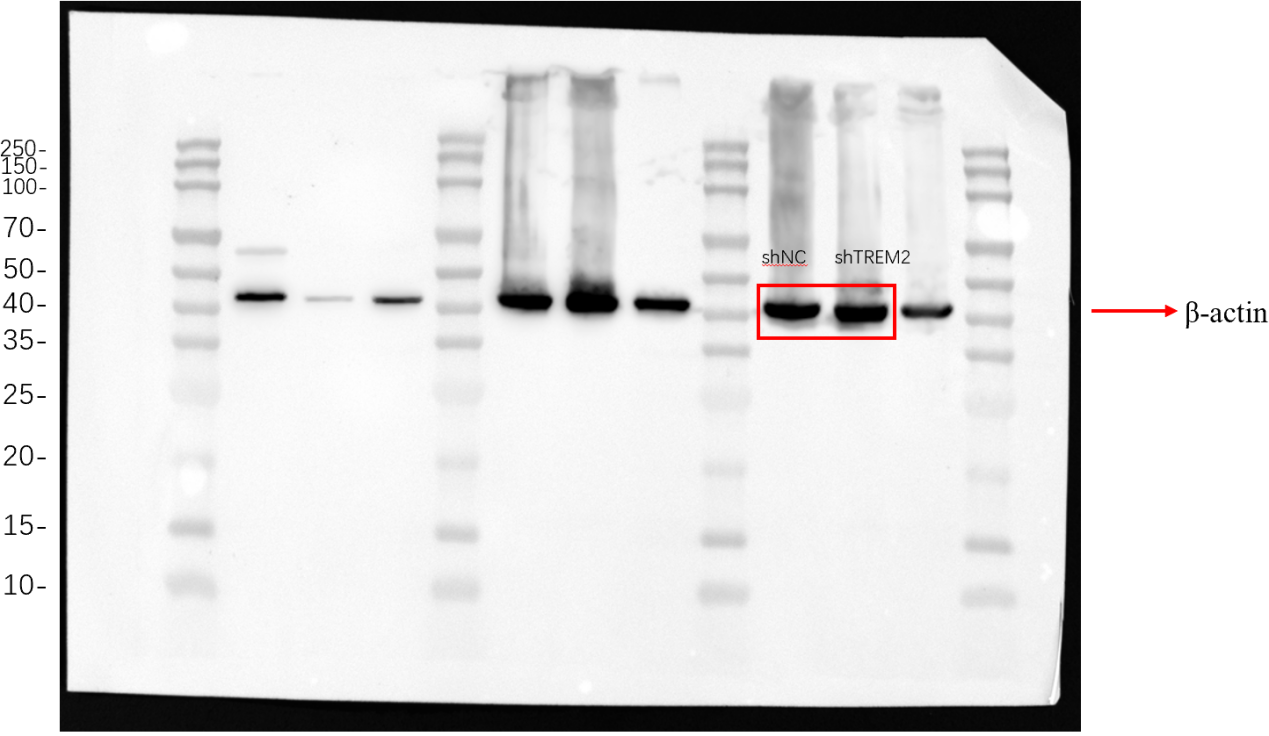
**

**
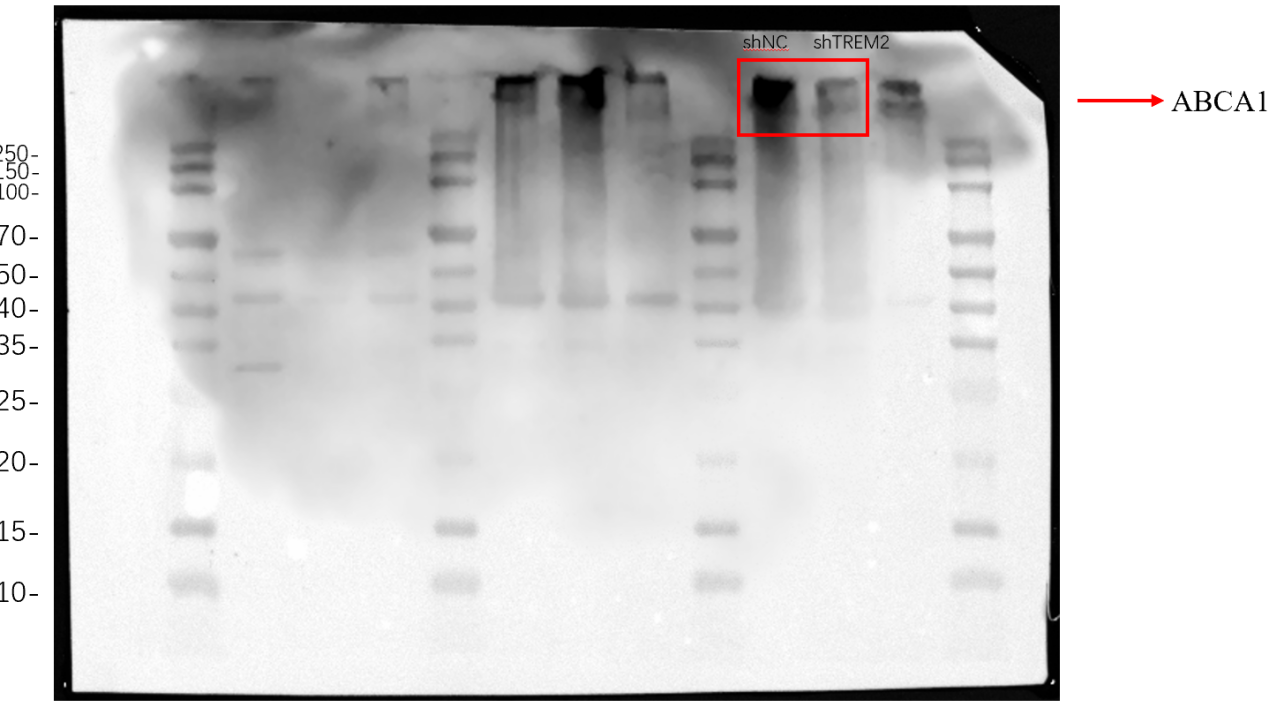
**

**③**


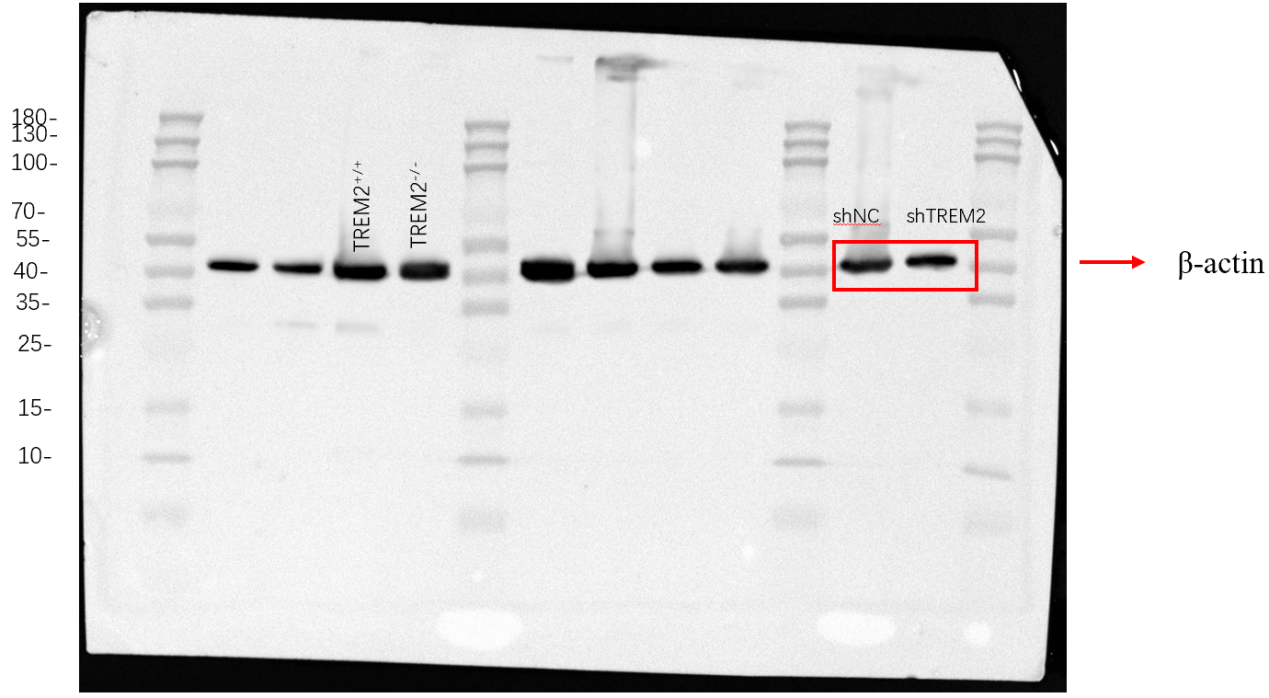


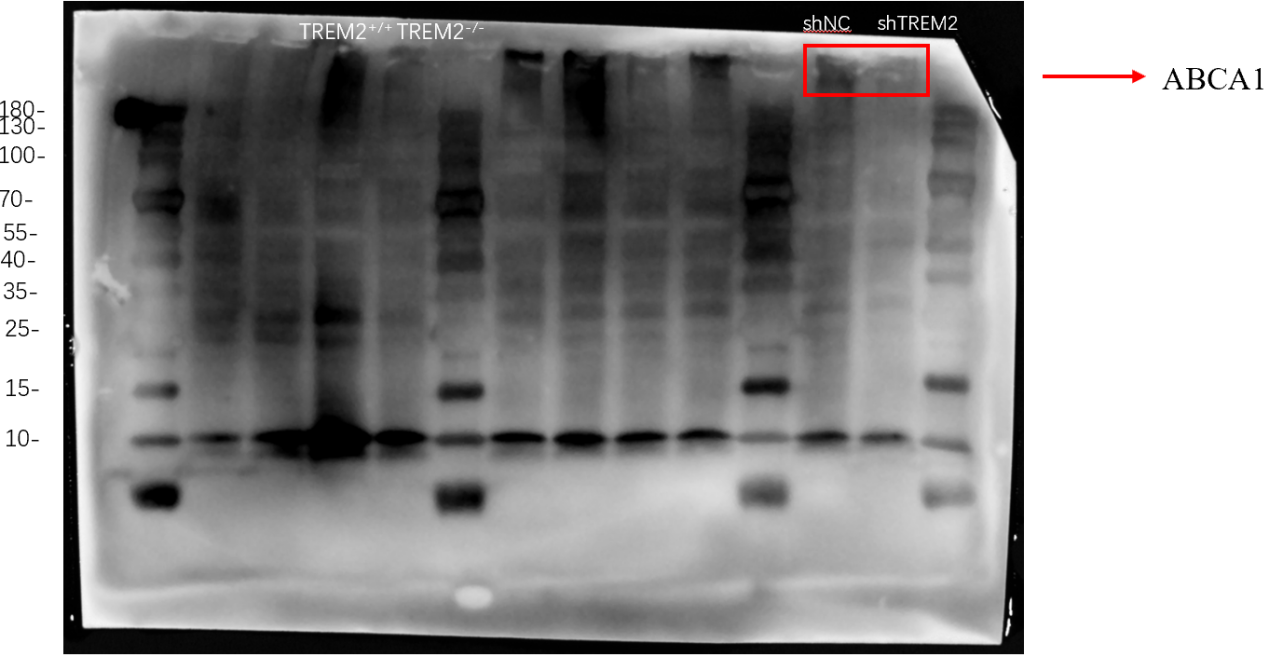


**Figure 6J:**

**①**


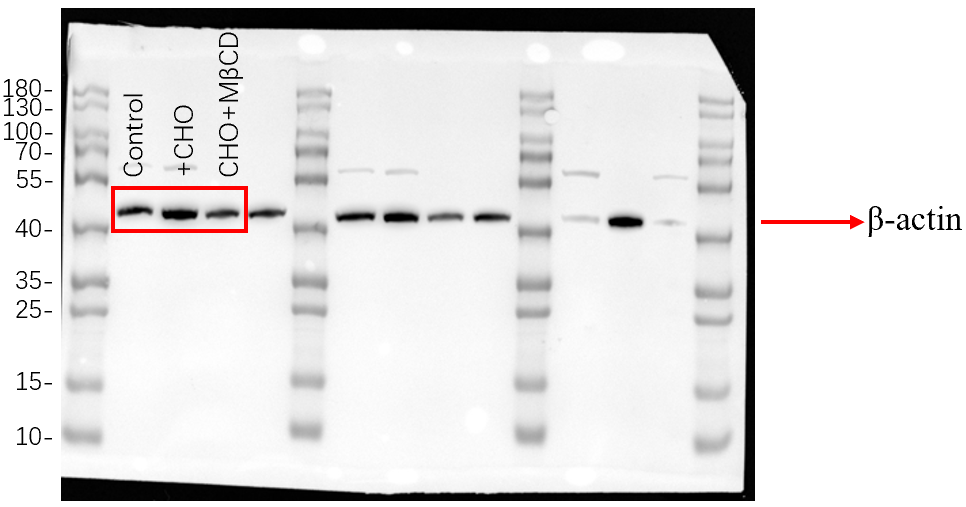


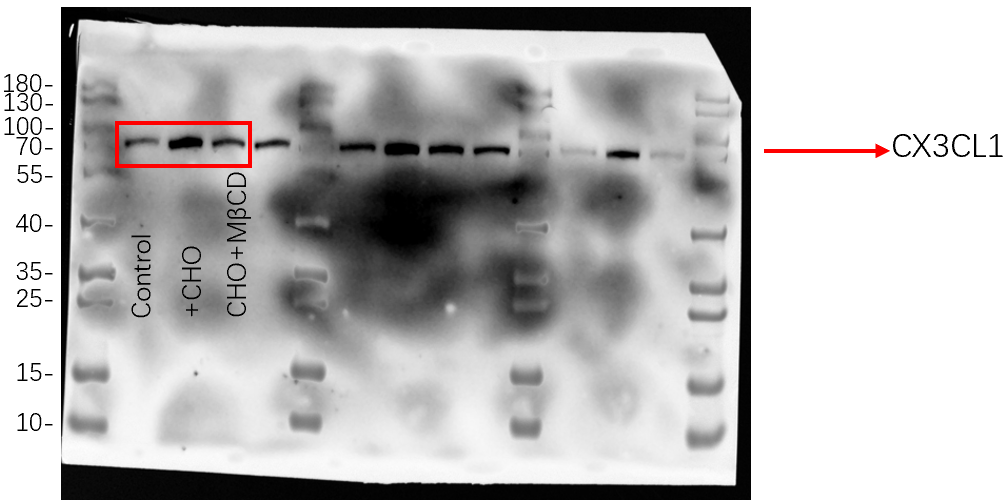


**②**

**
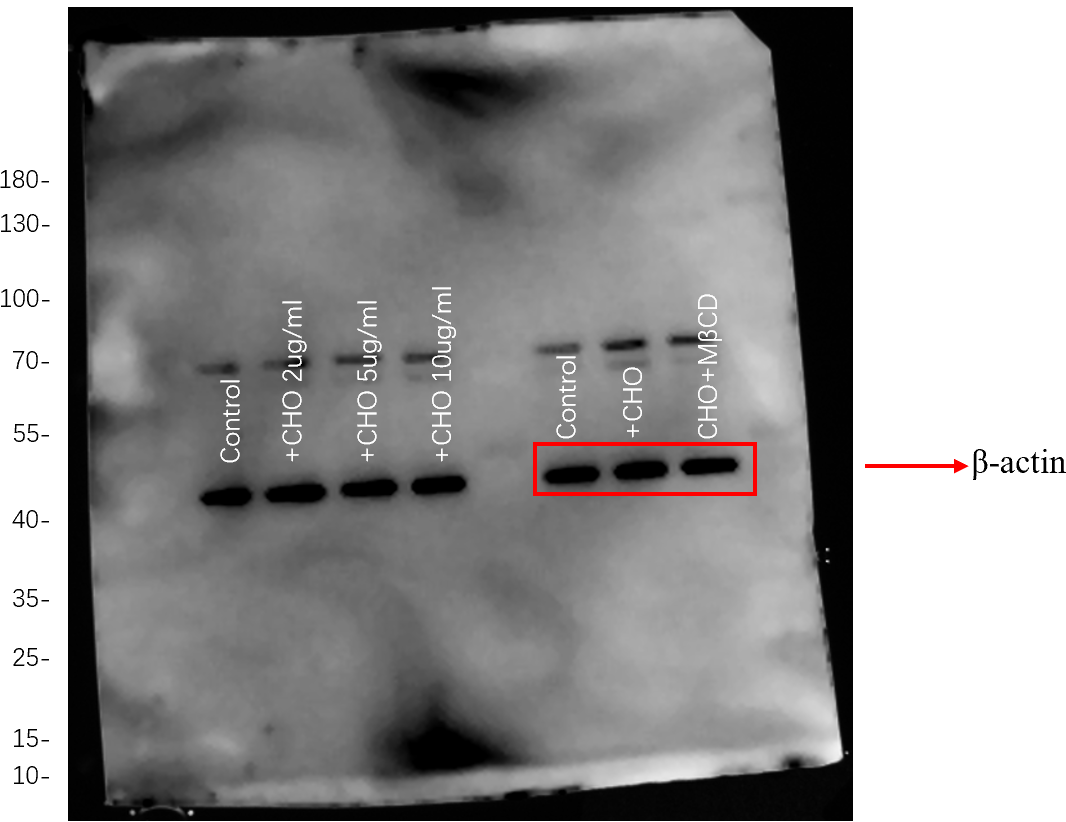
**

**
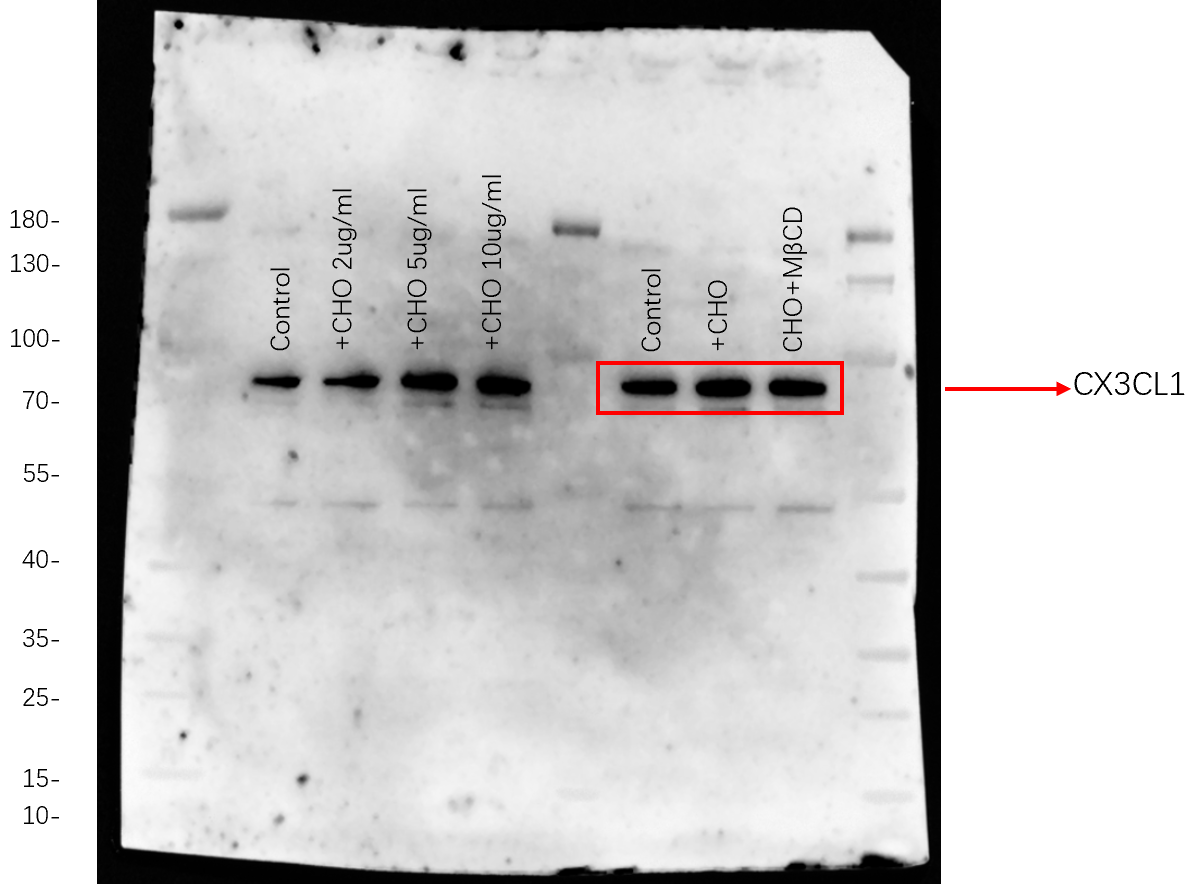
**

**③**


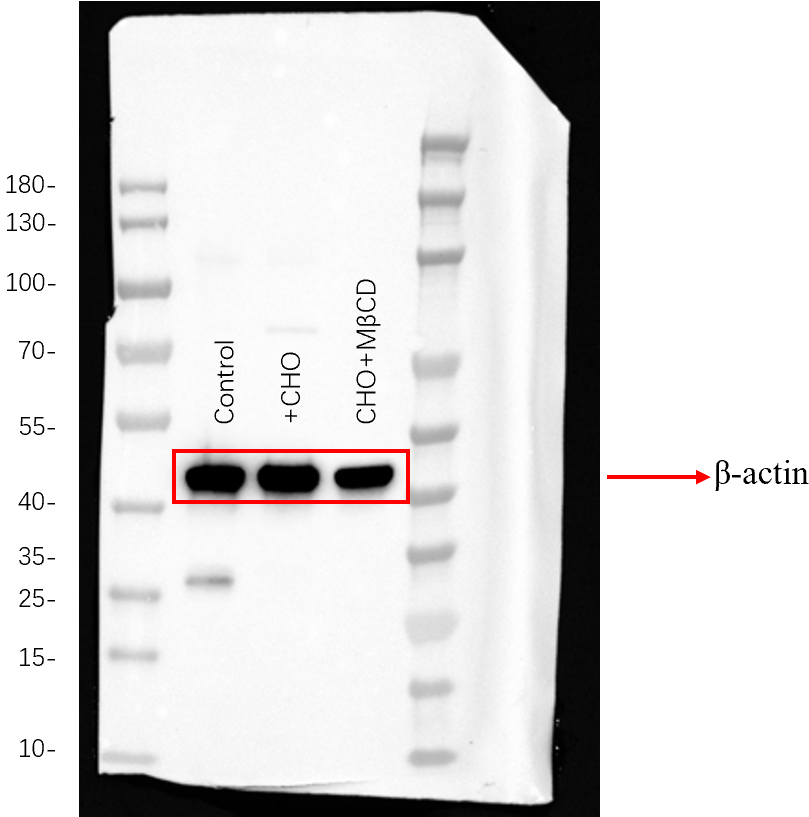


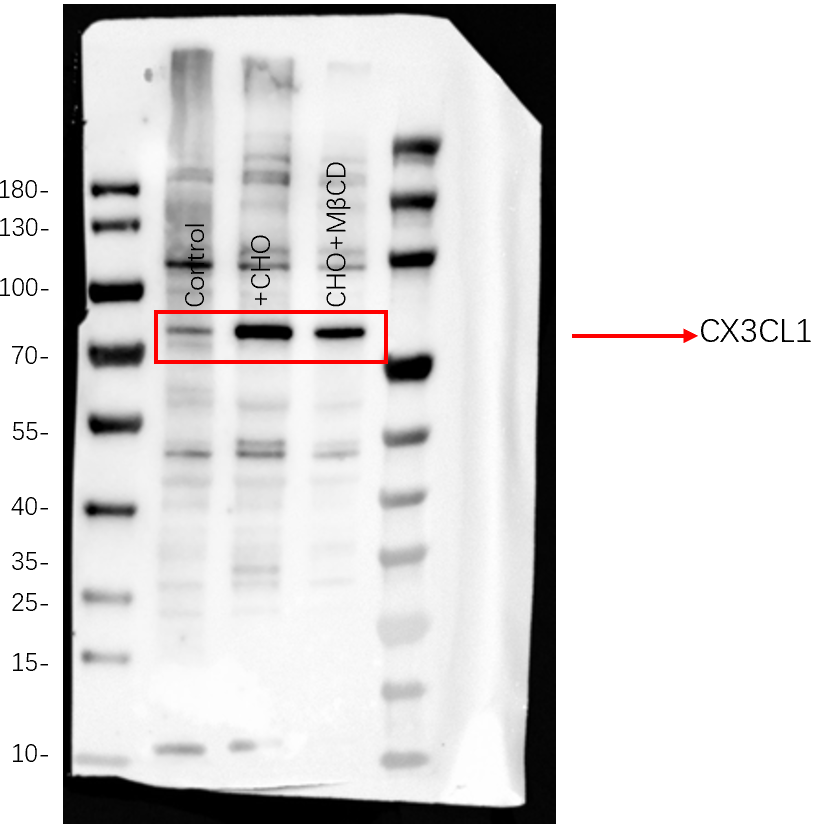


**Figure 6M:**

**①**


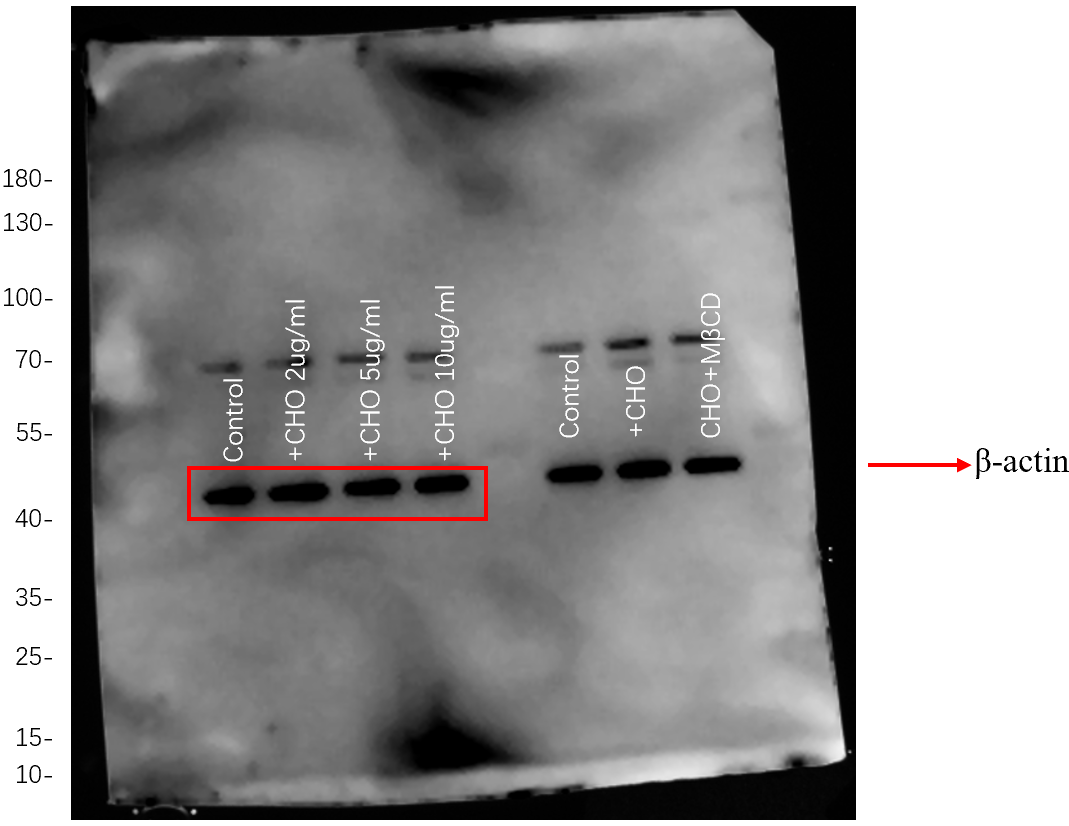


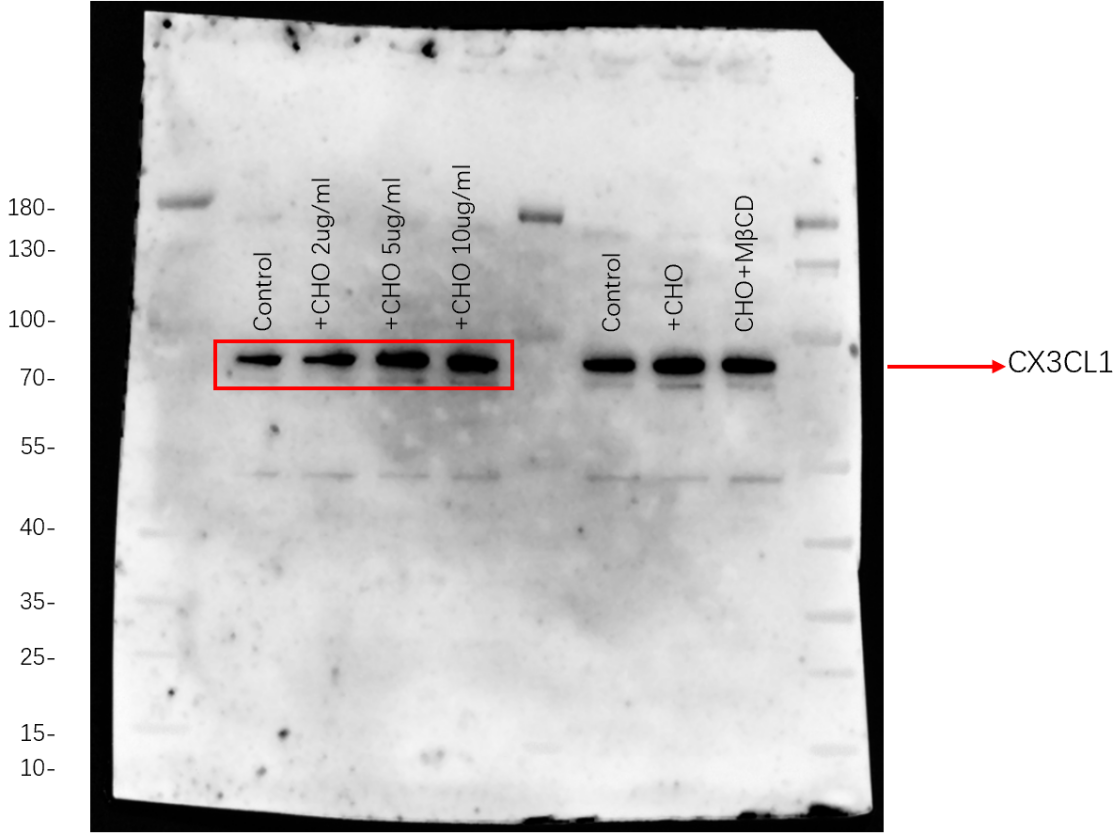


**②③**

**
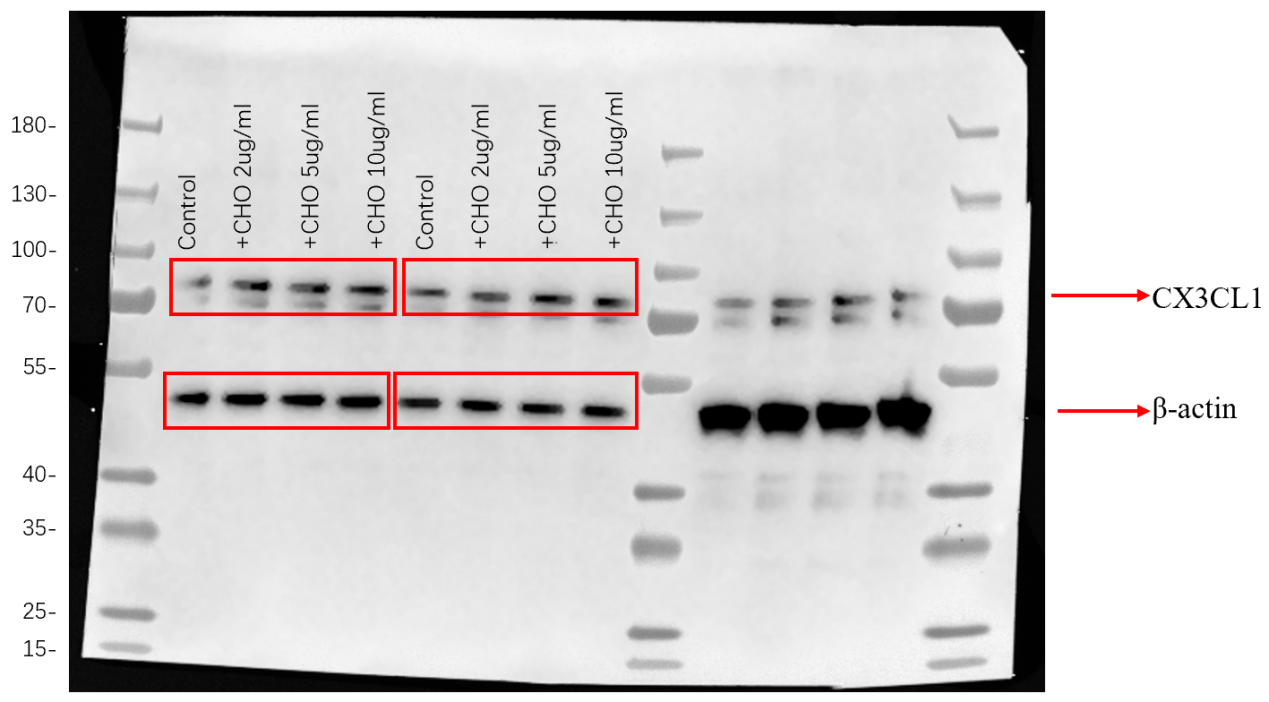
**

**sFigure 6C:**

**①**


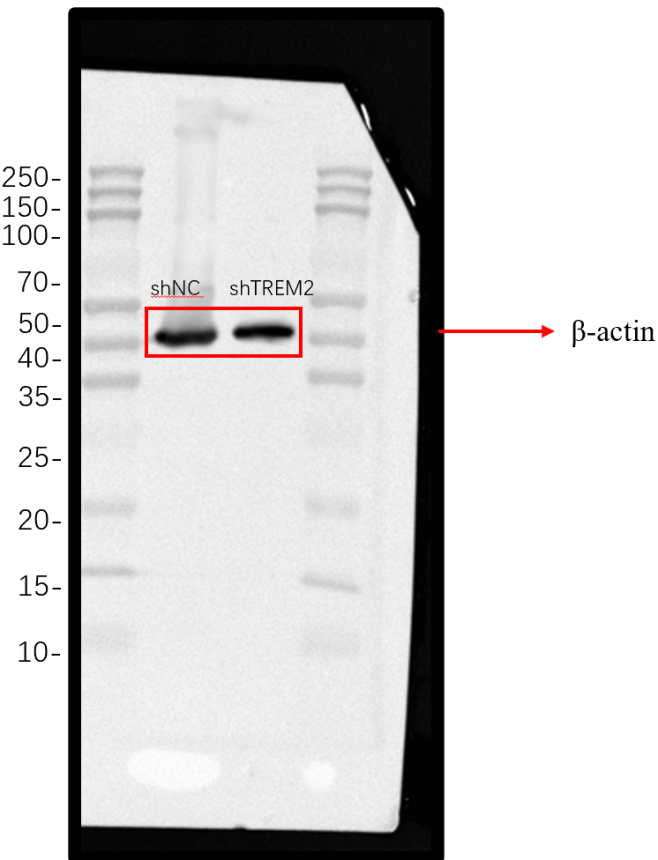

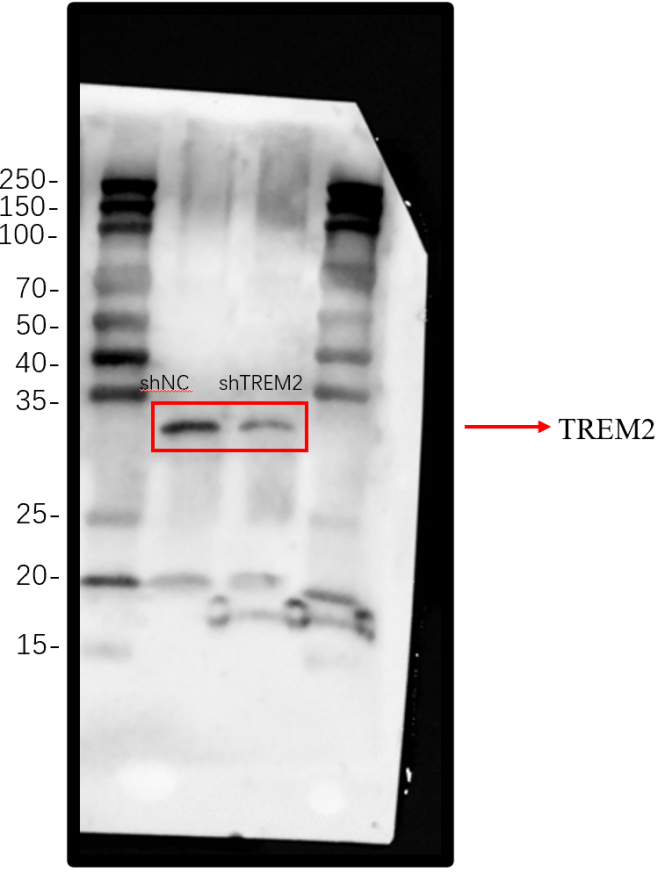


**②③**


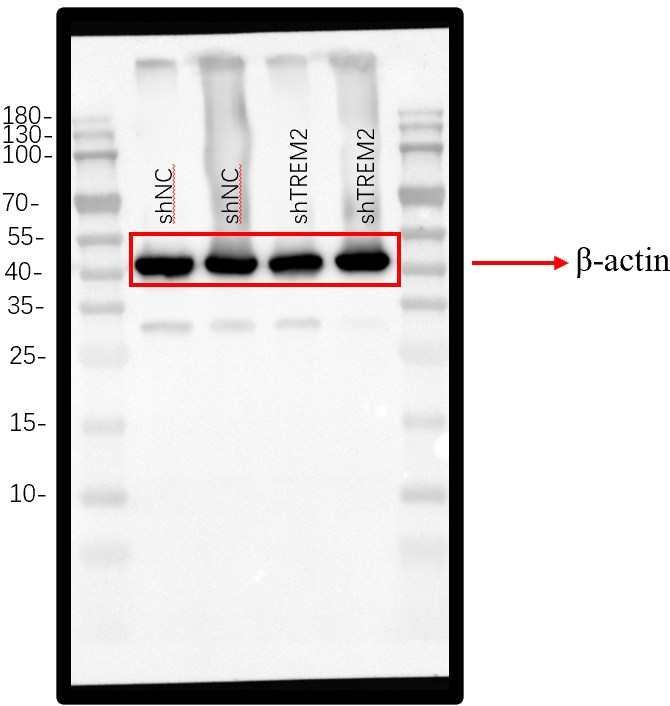


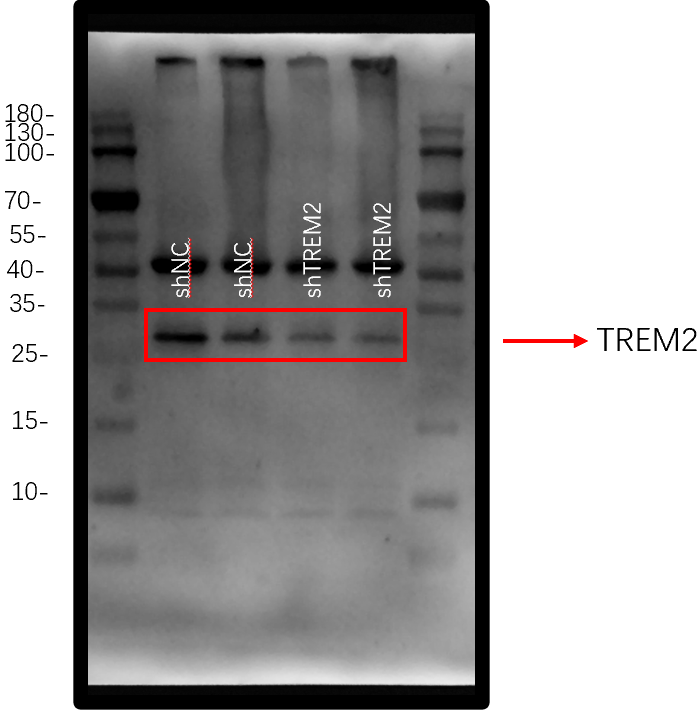


**sFigure 6E:**

**①**


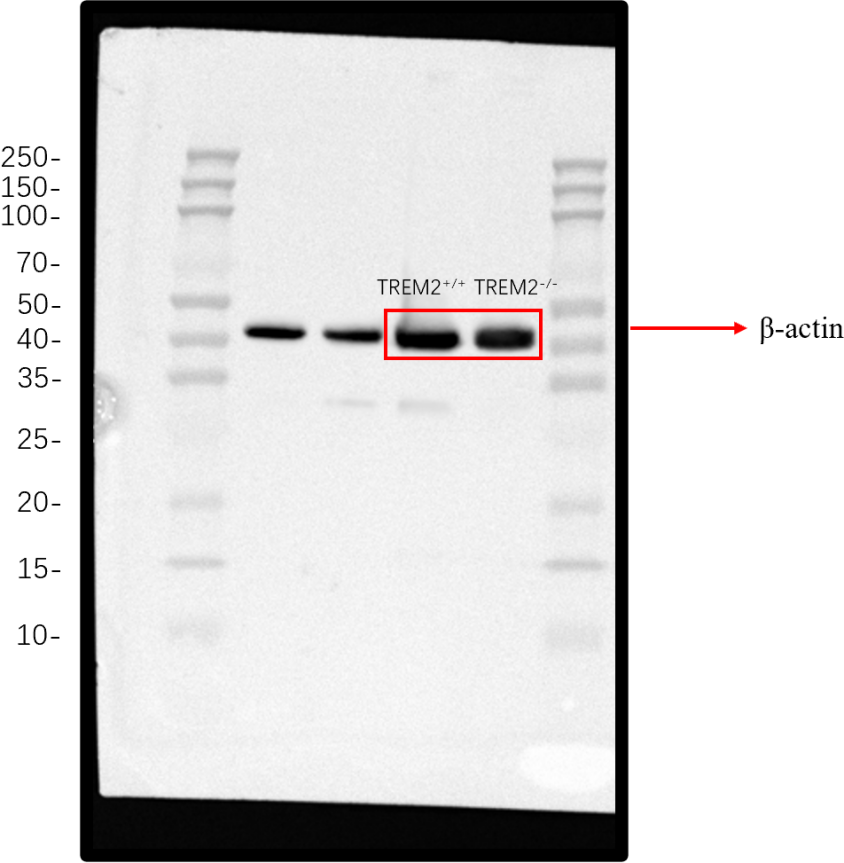


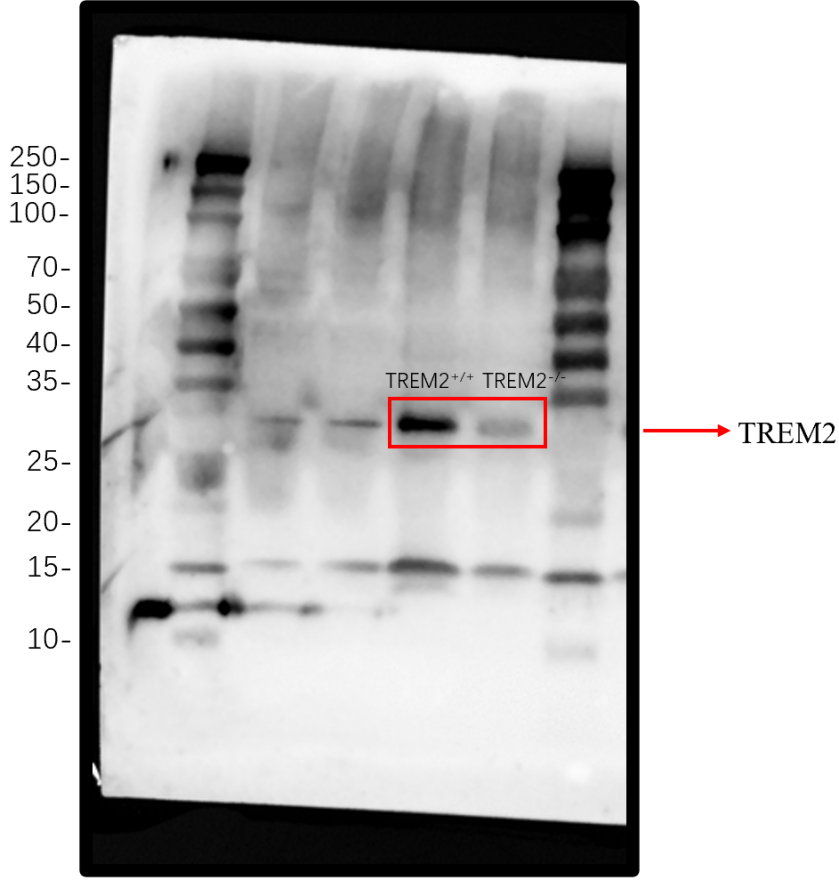


**②**


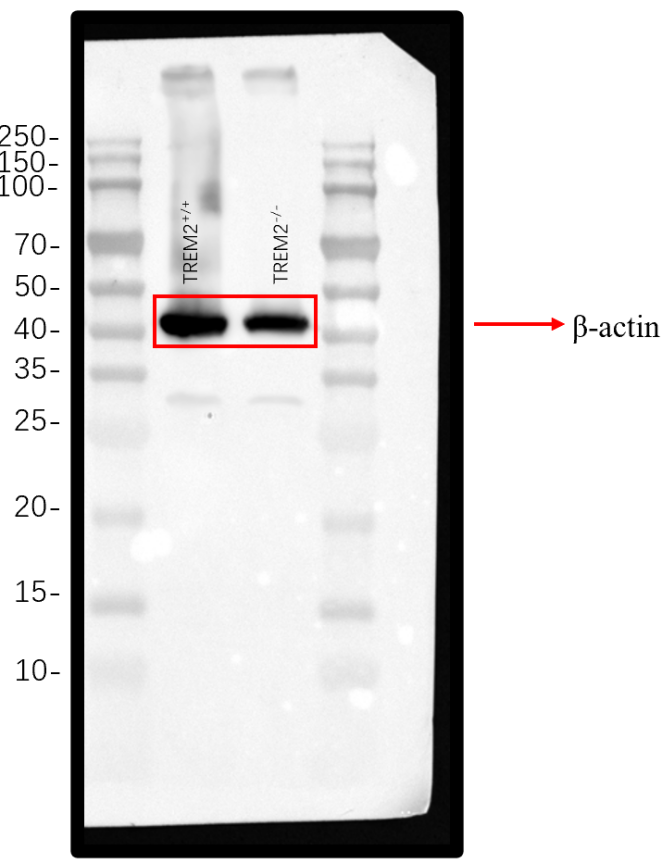


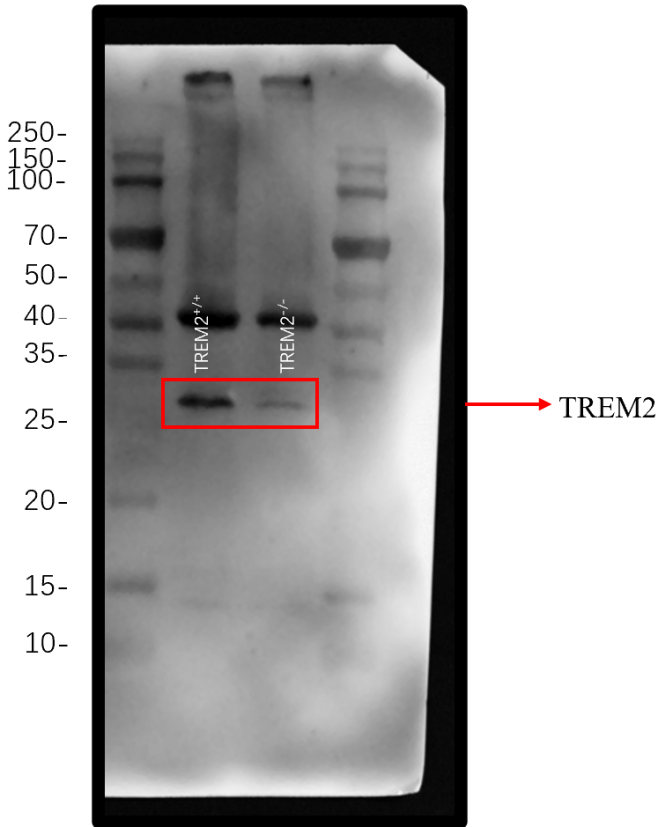


**③**


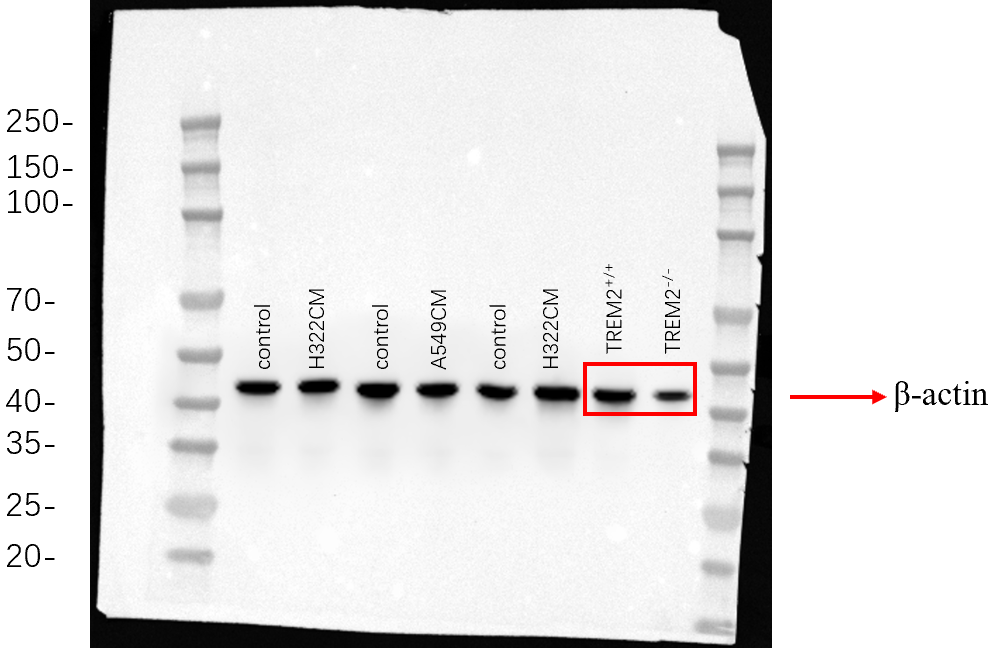


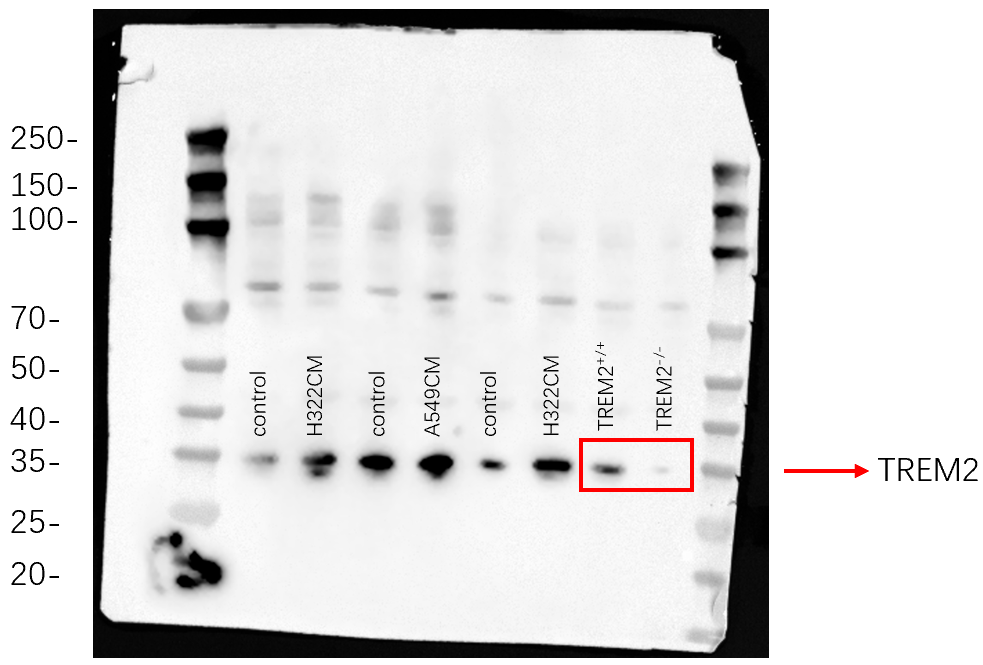

Supplement: Supplementary file 4 — Supporting Information [file ADVS-13-e06995-s001.docx]
